# Mitochondrial Damage‐Induced Innate Immune Activation in Vascular Smooth Muscle Cells Promotes Chronic Kidney Disease‐Associated Plaque Vulnerability

**DOI:** 10.1002/advs.202002738

**Published:** 2021-01-06

**Authors:** Xianjin Bi, Changhong Du, Xinmiao Wang, Xue‐Yue Wang, Wenhao Han, Yue Wang, Yu Qiao, Yingguo Zhu, Li Ran, Yong Liu, Jiachuan Xiong, Yinghui Huang, Mingying Liu, Chi Liu, Chunyu Zeng, Junping Wang, Ke Yang, Jinghong Zhao

**Affiliations:** ^1^ Department of Nephrology the Key Laboratory for the Prevention and Treatment of Chronic Kidney Disease of Chongqing Kidney Center of PLA Xinqiao Hospital Army Medical University (Third Military Medical University) Chongqing 400037 China; ^2^ State Key Laboratory of Trauma Burns and Combined Injury Institute of Combined Injury Chongqing Engineering Research Center for Nanomedicine College of Preventive Medicine Army Medical University (Third Military Medical University) Chongqing 400038 China; ^3^ Laboratory of Stem Cell & Developmental Biology Department of Histology and Embryology College of Basic Medical Sciences Army Medical University (Third Military Medical University) Chongqing 400038 China; ^4^ Department of Cardiology Daping Hospital Army Medical University (Third Military Medical University) Chongqing 400042 China

**Keywords:** atherosclerosis, chronic kidney disease, cyclic GMP‐AMP synthase‐stimulator of interferon genes pathway, plaque vulnerability, vascular smooth muscle cell

## Abstract

Chronic kidney disease (CKD) is associated with accelerated atherosclerosis progression and high incidence of cardiovascular events, hinting that atherosclerotic plaques in CKD may be vulnerable. However, its cause and mechanism remain obscure. Here, it is shown that apolipoprotein E‐deficient (ApoE^−/−^) mouse with CKD (CKD/ApoE^−/−^ mouse) is a useful model for investigating the pathogenesis of plaque vulnerability, and premature senescence and phenotypic switching of vascular smooth muscle cells (VSMCs) contributes to CKD‐associated plaque vulnerability. Subsequently, VSMC phenotypes in patients with CKD and CKD/ApoE^−/−^ mice are comprehensively investigated. Using multi‐omics analysis and targeted and VSMC‐specific gene knockout mice, VSMCs are identified as both type‐I‐interferon (IFN‐I)‐responsive and IFN‐I‐productive cells. Mechanistically, mitochondrial damage resulting from CKD‐induced oxidative stress primes the cyclic GMP‐AMP synthase‐stimulator of interferon genes (cGAS‐STING) pathway to trigger IFN‐I response in VSMCs. Enhanced IFN‐I response then induces VSMC premature senescence and phenotypic switching in an autocrine/paracrine manner, resulting in the loss of fibrous cap VSMCs and fibrous cap thinning. Conversely, blocking IFN‐I response remarkably attenuates CKD‐associated plaque vulnerability. These findings reveal that IFN‐I response in VSMCs through immune sensing of mitochondrial damage is essential for the pathogenesis of CKD‐associated plaque vulnerability. Mitigating IFN‐I response may hold promise for the treatment of CKD‐associated cardiovascular diseases.

## Graphical abstract

Oxidative stress‐induced mitochondrial damage under chronic kidney disease (CKD) milieu primes the cyclic GMP‐AMP synthase‐stimulator of interferon genes (cGAS‐STING) pathway in vascular smooth muscle cells (VSMCs) to trigger type‐I‐interferon response, which induces VSMC premature senescence and phenotypic switching in an autocrine/paracrine manner and finally results in plaque vulnerability.

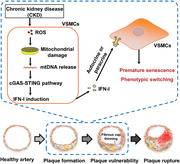

## 1 Introduction

Chronic kidney disease (CKD) is a serious health problem and is increasing rapidly worldwide. The lifespan of CKD patients is shortened; cardiovascular diseases (CVD) account for premature death in more than 50% of patients with CKD.^[^ ^1^ ^]^ Atherosclerosis (AS) is the leading cause of cardiovascular morbidity and mortality in patients with CKD, and its progression is often accelerated.^[^ ^2^, ^3^, ^4^ ^]^ Notably, thrombotic events are more likely to occur in patients with CKD, as well as in apolipoprotein E‐deficient (ApoE^−/−^) mice with CKD (CKD/ApoE^−/−^ mice),^[^ ^4^ ^]^ hinting that the plaques in CKD may exhibit some characters of vulnerability and be susceptible to rupture.

AS is a common aging‐related vascular disease with unfavorable outcomes. The major clinical consequences of AS, such as, myocardial infarction or stroke, are not a result of the gradual narrowing of the lumen, but rather that of thrombotic events associated with the rupture of a vulnerable plaque or endothelial erosion.^[^ ^5^ ^]^ Over the past decades, considerable efforts have been devoted to investigating the pathogenesis of plaque rupture and searching for a therapeutic strategy. However, the factors controlling plaque stability, as well as, the underlying mechanisms are still far from being elucidated.

Plaque stability and the risk of plaque rupture are primarily related to the composition of the plaques.^[^ ^6^, ^7^ ^]^ Postmortem and clinical imaging studies have identified several features of vulnerable plaques, such as thin fibrous cap and large necrotic core.^[^ ^8^ ^]^ Among these features, fibrous cap, which separates the thrombogenic lesion contents from the blood, is a critical predictor of plaque stability and plaque rupture.^[^ ^7^, ^9^, ^10^ ^]^ Fibrous cap primarily consists of vascular smooth muscle cells (VSMCs) and VSMC‐secreted matrix. Pathologically, vulnerable plaque is characterized by a thin fibrous cap with deficient VSMCs,^[^ ^11^ ^]^ highlighting a central role for VSMC loss in the pathogenesis of plaque vulnerability.

VSMC contents, which are closely associated with atherogenesis and fibrous cap thickness, are determined by cell activities, including proliferation, apoptosis, migration, phenotypic switching, and senescence. Of note, at the late stage of AS development, fibrous cap formation and repair require VSMC proliferation; thus, VSMC viability maintenance is thus beneficial for plaque stability.^[^ ^6^ ^]^ However, VSMCs from late‐stage plaques show poor proliferation, apoptosis, and premature senescence.^[^ ^6^, ^11^ ^]^ It has been reported that VSMC apoptosis and premature senescence can induce plaque vulnerability and rupture;^[^ ^11^, ^12^, ^13^ ^]^ however, the underlying mechanisms are not well known.

Excessive inflammation or failed inflammation resolution is a major contributor to plaque development and plaque vulnerability. Various inflammatory cytokines, such as interleukins can affect VSMC viability.^[^ ^14^, ^15^ ^]^ As reported, inflammatory cells are the main sources of vascular inflammation. Nevertheless, vascular‐resident cells including VSMCs can also switch to an inflammatory secretory phenotype upon vascular injury.^[^ ^6^ ^]^ However, the specific role of VSMCs in modulating local vascular inflammation was rarely explored.

In this study, we demonstrated that CKD/ApoE^−/−^ mouse was a useful animal model for investigating the pathogenesis of plaque vulnerability. Interestingly, we identified VSMCs as being both type‐I‐interferon (IFN‐I)‐responsive and IFN‐I‐productive. We then showed that VSMCs could sense oxidative stress‐induced mitochondrial damage through the cGAS‐STING pathway, thereby triggering IFN‐I response to induce themselves premature senescence and phenotypic switching and consequently promoting plaque vulnerability. In contrast, attenuation of IFN‐I response remarkably mitigated plaque vulnerability. Our results uncover a common mechanism for the pathogenesis of plaque vulnerability, and afford a promising strategy for the treatment of CKD‐associated CVD.

## 2 Results

### 2.1 Chronic Kidney Disease/ApoE^−/−^ Mouse is a Useful Model for Investigating the Pathogenesis of Plaque Vulnerability

**Figure 1:**
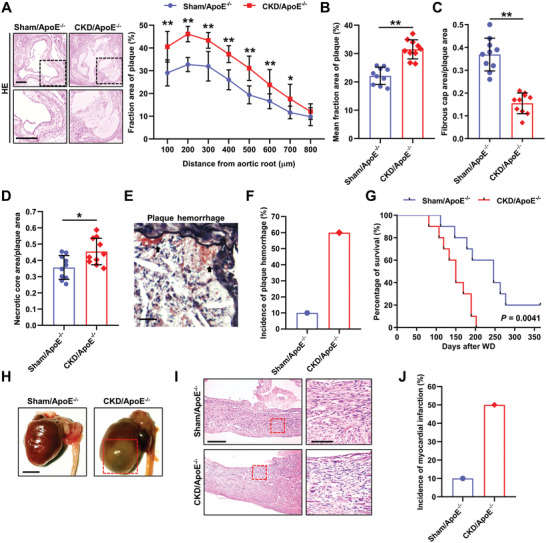
CKD/ApoE^−/−^ mouse is a useful model for investigating the pathogenesis of plaque vulnerability. A) Representative hematoxylin and eosin (HE) staining and the fraction area of multiple sections of aortic root plaques in Sham/ApoE^−/−^ and CKD/ApoE^−/−^ mice (*n* = 10). Scale bars, 200 µm. The box indicates the region magnified in the lower panels. B) Mean fraction area of aortic root plaques in Sham/ApoE^−/−^ and CKD/ApoE^−/−^ mice (*n* = 10). C,D) Relative areas of fibrous cap and necrotic core in aortic root plaques of Sham/ApoE^−/−^ and CKD/ApoE^−/−^ mice (*n* = 10). E) Representative Movat's stain in BCA plaque. The arrow indicates plaque hemorrhage. Scale bar, 25 µm. F) Incidence of plaque hemorrhage in BCA plaques of Sham/ApoE^−/−^ and CKD/ApoE^−/−^ mice. G) Kaplan–Meier survival analysis of Sham/ApoE^−/−^ and CKD/ApoE^−/−^ mice (*n* = 10). H) Representative hearts at postmortem examination of Sham/ApoE^−/−^ and CKD/ApoE^−/−^ mice. The red box indicates the myocardial infarction region manifested by pale and discolored patches, accompanied by enlarged heart. Scale bar, 5 mm. I) Representative HE staining of the hearts from Sham/ApoE^−/−^ and CKD/ApoE^−/−^ mice. The box indicates the myocardial infarction region magnified in the right panels. Scale bar (left panels), 250 µm. Scale bar (right panels), 100 µm. J) Incidence of myocardial infarction in Sham/ApoE^−/−^ and CKD/ApoE^−/−^ mice. Data represent mean ± SD. *p < 0.05, **p < 0.01, two‐tailed Student's *t*‐test was applied to (A–D). Log‐rank (Mantel‐Cox) test was applied to (G).

To better investigate the pathogenesis of AS and plaque vulnerability in mice, we created a CKD/ApoE^−/−^ mouse model by performing a 5/6 nephrectomy in ApoE^−/‐^ mice (Figure S1A and Table S1, Supporting Information). Consistently, CKD/ApoE^−/−^ mice exhibited dramatically accelerated plaque growth in comparison to sham‐operated ApoE^−/−^ (Sham/ApoE^−/−^) mice, especially after 12 weeks of a high‐cholesterol Western diet (WD) feeding (**Figure** **1**A,B; Figure S1B–F, Supporting Information). Interestingly, plaque composition in the aortic root and brachiocephalic artery (BCA) was dramatically altered in CKD/ApoE^−/−^ mice, manifested by reduced fibrous cap areas and increased necrotic core areas (Figure 1C,D; Figure S1G, Supporting Information). These characteristics are associated with reduced mechanical stability of plaques, which may render plaques susceptible to rupture. In humans, plaque rupture implies fibrous cap disruption, accompanied by either atherothrombosis or plaque hemorrhage.^[^ ^8^ ^]^ Although obvious fibrous cap disruption or atherothrombosis was hardly detected in the mouse model, a higher frequency of plaque hemorrhage was observed in BCA plaques of CKD/ApoE^−/−^ mice (Figure 1E,F). These results indicated that CKD/ApoE^−/−^ mice had the characteristics of plaque vulnerability, and higher susceptibility to plaque disruption. In addition, survival analysis revealed that CKD/ApoE^−/−^ mice tended to die earlier, and myocardial infarction was frequently observed at postmortem examination (Figure 1G–J, and Table S2, Supporting Information). All these findings demonstrated that CKD/ApoE^−/−^ mouse was a useful mouse model for the investigation of plaque vulnerability.

### 2.2 Vascular Smooth Muscle Cell Premature Senescence and Phenotypic Switching Contribute to Plaque Vulnerability in Chronic Kidney Disease/ApoE^−/−^ Mice

**Figure 2:**
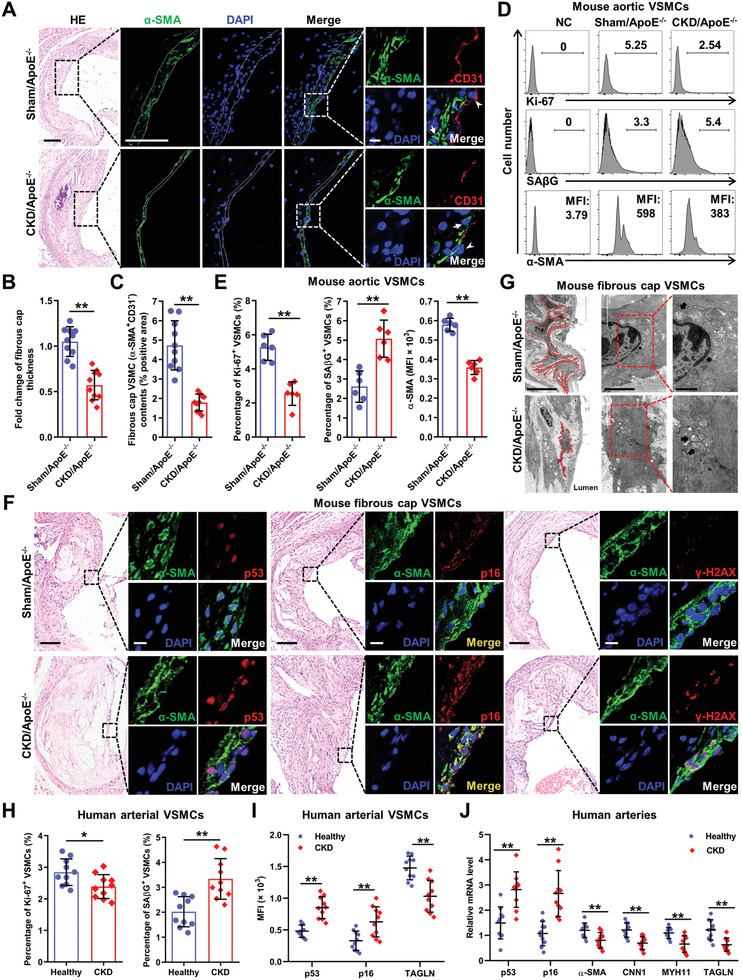
VSMC premature senescence and phenotypic switching contribute to plaque vulnerability in CKD/ApoE^−/−^ mice. A) Representative images of HE, *α*‐SMA, and CD31 staining in aortic root plaques of Sham/ApoE^−/−^ and CKD/ApoE^−/−^ mice. The dashed lines delineate the fibrous cap area. The box indicates the region magnified in the right panels. The arrow indicates fibrous cap VSMCs (*α*‐SMA^+^CD31^−^). The arrowhead indicates endothelial cells (*α*‐SMA^−^CD31^+^). Scale bar (HE), 100 µm. Scale bar (*α*‐SMA staining), 40 µm. Scale bar (*α*‐SMA and CD31 double staining), 10 µm. B,C) Quantification of the fold change of fibrous cap thickness and fibrous cap VSMC (*α*‐SMA^+^CD31^−^) contents in aortic root plaques of Sham/ApoE^−/−^ and CKD/ApoE^−/−^ mice (*n* = 10). D) Representative FC analysis of Ki‐67, SA*β*G, and *α*‐SMA expression levels in aortic VSMCs of Sham/ApoE^−/−^ and CKD/ApoE^−/−^ mice. E) The percentages of Ki‐67^+^ and SA*β*G^+^ VSMCs, as well as, the mean fluorescence intensity (MFI) of *α*‐SMA in aortic VSMCs of Sham/ApoE^−/−^ and CKD/ApoE^−/−^ mice (*n* = 6). F) Representative images of HE, p53, p16, and *γ*‐H2AX staining in fibrous cap VSMCs of Sham/ApoE^−/−^ and CKD/ApoE^−/−^ mice. Scale bar (HE), 100 µm. Scale bar (IF), 10 µm. G) Representative transmission electron microscopy images of fibrous cap VSMCs in aortic root of Sham/ApoE^−/−^ and CKD/ApoE^−/−^ mice. The dashed lines delineate the fibrous cap VSMCs. The box indicates the region magnified in the right panels. The arrow indicates the swollen and vacuolated mitochondria in fibrous cap VSMCs. Scale bar (left panels), 10 µm. Scale bar (middle panels), 2 µm. Scale bar (right panels), 1 µm. H,I) The percentages of Ki‐67^+^ and SA*β*G^+^ VSMCs, as well as, the MFIs of p53, p16, and TAGLN in arterial VSMCs of healthy people and CKD patients (*n* = 10). J) Relative mRNA expression levels of p53, p16, *α*‐SMA, CNN1, MYH11, and TAGLN in arteries from healthy people and CKD patients (*n* = 10). Data represent mean ± SD. **p < 0.05, **p < 0.01, two‐tailed Student's *t*‐test. DAPI, 4,6‐diamidino‐2‐phenylindole; NC, negative control.

The fibrous cap is a strong determinant of plaque stability and the likelihood of plaque rupture. We next stained alpha‐smooth muscle actin (*α*‐SMA), a VSMC marker, to further analyze the morphometry of fibrous cap. A significantly decrease in fibrous cap thickness was observed in CKD/ApoE^−/−^ mice relative to Sham/ApoE^−/−^ mice (**Figure** **2**A,B; Figure S1H,I, Supporting Information). Concurrently, the fibrous cap VSMC (*α*‐SMA^+^CD31^−^) contents in CKD/ApoE^−/−^ mice were much less than those in Sham/ApoE^−/−^ mice (Figure 2A,C; Figure S1H,I, Supporting Information), suggesting that VSMC loss might contribute to plaque vulnerability in CKD/ApoE^−/−^ mice.

Then, we searched for the reason responsible for VSMC loss. First, we analyzed the cell‐cycle state of VSMCs using flow cytometry (FC; Figure S2A, Supporting Information), and apoptosis using immunofluorescence (IF). We found that VSMCs from CKD/ApoE^−/−^ mice displayed markedly reduced proliferation compared with those from Sham/ApoE^−/−^ mice (Figure 2D,E), while the apoptosis level in the fibrous cap was comparable between Sham/ApoE^−/−^ and CKD/ApoE^−/−^ mice (Figure S2B, Supporting Information). Cell‐cycle arrest without undergoing cell death process is a hallmark of cellular senescence, which usually contributes to aging‐related diseases.^[^ ^16^ ^]^ As expected, the expressions of senescence markers, including senescence‐associated *β*‐galactosidase activity (SA*β*G), phosphorylated histone H2AX (*γ*‐H2AX), p53, and p16, were significantly increased in the aortas and fibrous cap VSMCs of CKD/ApoE^−/−^ mice (Figure 2D,F; Figure S2C–H, Supporting Information). Interestingly, p53 was mainly expressed in the nucleus, while p16 was mainly expressed in the cytoplasm in senescent VSMCs (Figure 2F). Meanwhile, fibrous cap VSMCs of CKD/ApoE^−/−^ mice exhibited aging‐related abnormal cell morphology,^[^ ^17^ ^]^ such as, elongation and disconnections between VSMCs in vivo (Figure 2G). Since various cell types other than VSMCs within lesions have been reported to express *α*‐SMA,^[^ ^18^ ^]^ we further verified these findings in CKD mice without WD, arteries from CKD patients, as well as, maturely differentiated human VSMCs. Similar to that in CKD mice, increased VSMC premature senescence was also observed in patients with CKD (Figure 2H–J). In vitro, Human aortic smooth muscle cells (hVSMCs) incubated with serum from CKD stage 5 patients showed increased senescence in a time‐dependent manner, without affecting the apoptosis level (Figure S3, Supporting Information), confirming the findings in mouse model and patients with CKD.

We also found that the expressions of *α*‐SMA and other VSMC markers, including myosin heavy chain 11 (MYH11), calponin 1 (CNN1), transgelin (TAGLN) were significantly reduced in the aortas and fibrous cap VSMCs of CKD/ApoE^−/−^ mice comparing to Sham/ApoE^−/−^ mice (Figure 2D,E; Figure S4A, Supporting Information). All these data suggested a state of VSMC phenotypic switching. Indeed, downregulation of VSMC markers in VSMCs under CKD conditions was confirmed both in vivo and in vitro (Figure 2I,J; Figure S4B–G, Supporting Information). Although enhanced VSMC migration was also observed (Figure S4H, Supporting Information), it might not contribute to VSMC loss as VSMC migration usually has a positive effect on VSMC contents.^[^ ^6^ ^]^

Collectively, these results suggested that the induction of VSMC premature senescence and phenotypic switching might be the main cause for VSMC loss and plaque vulnerability in CKD/ApoE^−/−^ mice.

### 2.3 Type‐I‐Interferon Response Promotes Vascular Smooth Muscle Cell Premature Senescence and Phenotypic Switching in Chronic Kidney Disease/ApoE^−/−^ Mice

**Figure 3:**
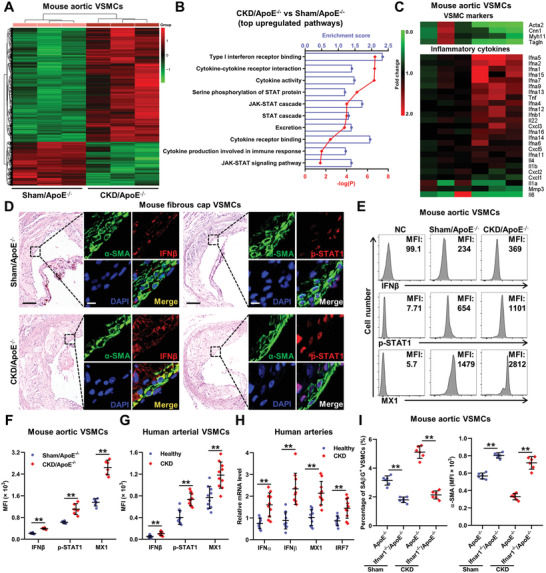
IFN‐I response promotes VSMC premature senescence and phenotypic switching in CKD/ApoE^−/−^ mice. A) The heat maps show genes that differently expressed in aortic VSMCs of Sham/ApoE^−/−^ and CKD/ApoE^−/−^ mice. B) KEGG and GO enrichment analysis of top upregulated pathways in aortic VSMCs of CKD/ApoE^−/−^ mice. C) The heat maps show the relative expression levels of VSMC markers and inflammation cytokines based on the integrated microarray analysis of aortic VSMCs of Sham/ApoE^−/−^ and CKD/ApoE^−/−^ mice. D) Representative images of HE, IFN*β* and p‐STAT1 staining in fibrous cap VSMCs of Sham/ApoE^−/−^ and CKD/ApoE^−/−^ mice. Scale bar (HE), 100 µm. Scale bar (IF), 10 µm. E) Representative FC analysis of IFN*β*, p‐STAT1, and MX1 expressions in aortic VSMCs of Sham/ApoE^−/−^ and CKD/ApoE^−/−^ mice. F) The MFIs of IFN*β*, p‐STAT1, and MX1 in aortic VSMCs of Sham/ApoE^−/−^ and CKD/ApoE^−/−^ mice (*n* = 6). G) The MFIs of IFN*β*, p‐STAT1, and MX1 in arterial VSMCs of healthy people and CKD patients (*n* = 10). H) Relative mRNA expression levels of IFN*α*, IFN*β*, IRF7, and MX1 in arteries from healthy people and CKD patients (*n* = 10). I) The percentage of SA*β*G^+^ VSMCs and the MFI of *α*‐SMA in VSMCs of Sham/ApoE^−/−^, Sham/Ifnar1^−/−^/ApoE^−/−^, CKD/ApoE^−/−^, and CKD/Ifnar1^−/−^/ApoE^−/−^ mice (*n* = 6). Data represent mean ± SD. **p < 0.01, two‐tailed Student's *t*‐test was applied to (F–H); one‐way ANOVA was applied to (I).

To identify the underlying mechanisms, we performed an integrated microarray analysis of aortic VSMCs from Sham/ApoE^−/−^ and CKD/ApoE^−/−^ mice and observed dramatically different gene expression patterns between them (**Figure** **3**A,C). Consistently, gene set enrichment analysis (GSEA) showed significant downregulation of G1‐S transition genes and VSMC markers (Figure S5A, Supporting Information). Notably, Kyoto Encyclopedia of Genes and Genomes (KEGG) and Gene Ontology (GO) enrichment analysis revealed obvious upregulation of genes related to inflammatory cytokine production, signaling and regulation (Figure 3C), suggesting the switching from a contractile phenotype to an inflammatory secretory phenotype of VSMCs in CKD/ApoE^−/−^ mice. In fact, inflammatory cytokines play essential roles in the initiation and maintenance of cellular senescence.^[^ ^19^ ^]^ Based on the analysis of inflammatory cytokines involved in senescence, robust upregulation of IFN‐I and activation of its downstream Janus kinase‐signal transducers and activators of transcription (JAK‐STAT) signaling were observed in VSMCs of CKD/ApoE^−/−^ mice (Figure 3B). IFN‐I family consists of more than 10 IFN*α* subtypes and a single IFN*β*. However, IFN‐I production occurs in two phases and IFN*α* production is mediated by the initial induction of IFN*β*.^[^ ^20^ ^]^ Therefore, we designed primers amplifying all of the IFN*α* subtypes and the single IFN*β* to detect IFN*α* and IFN*β* at the mRNA level, whereas IFN*β* at the protein level was detected using FC, IF, and enzyme‐linked immunosorbent assay (ELISA). STAT1 is known as the main effector of IFN‐I signaling and mediates the transcriptional of IFN‐stimulated genes (ISGs) including MX dynamin like GTPase 1 (MX1) and interferon regulatory factor 7 (IRF7).^[^ ^21^ ^]^ So, the changes of STAT1 and ISGs were also analyzed. As expected, significant increases in the expression levels of IFN*α*, IFN*β*, and ISGs, as well as, STAT1 activation were observed in the aortas and fibrous cap VSMCs of CKD/ApoE^−/−^ mice (Figure 3D; Figure S5B, Supporting Information). Similar changes were also observed in mouse aortas, human arteries, and VSMCs under CKD condition (Figure 3E–I; Figures S5C–G and S6, Supporting Information).

We next explored the influence of IFN‐I signaling activation on VSMCs. As shown, IFN*β* treatment induced mouse and human VSMC senescence in a dose‐dependent manner, accompanied by the decreased expression of *α*‐SMA (Figure S7, Supporting Information). Knockout or knockdown of IFN‐I receptor (IFNAR1), and its downstream STAT1, significantly abrogated CKD‐induced VSMC senescence and *α*‐SMA downregulation (Figure 3I; Figure S8, Supporting Information). These data hinted that IFN‐I signaling might play an important role in VSMC premature senescence and phenotypic switching in CKD milieu.

### 2.4 Cyclic GMP‐AMP Synthase‐Stimulator of Interferon Genes Pathway Activation is Responsible for Chronic Kidney Disease‐Induced Type‐I‐Interferon Response in Vascular Smooth Muscle Cells

**Figure 4:**
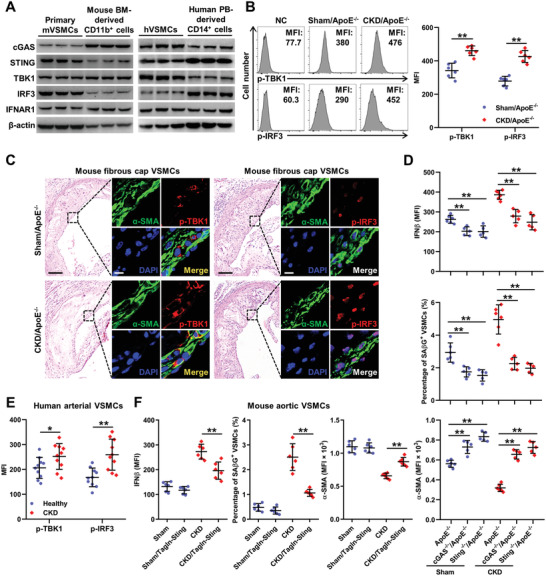
cGAS‐STING pathway activation is responsible for CKD‐induced IFN‐I response in VSMCs. A) Representative WB analysis of typical genes in the cGAS‐STING pathway including cGAS, STING, TBK1, IRF3, and IFNAR1 in indicated cells. B) Representative FC analysis and the quantification of p‐TBK1 and p‐IRF3 expression levels in aortic VSMCs of Sham/ApoE^−/−^ and CKD/ApoE^−/−^ mice (*n* = 6). C) Representative images of HE, p‐TBK1 and p‐IRF3 staining in fibrous cap VSMCs of Sham/ApoE^−/−^ and CKD/ApoE^−/−^ mice. Scale bar (HE), 100 µm. Scale bar (IF), 10 µm. D) The percentage of SA*β*G^+^ VSMCs, as well as the MFIs of IFN*β* and *α*‐SMA in aortic VSMCs from Sham/ApoE^−/−^ (*n* = 6), Sham/cGAS^−/−^/ApoE^−/−^ (*n* = 5), Sham/Sting^−/−^/ApoE^−/−^ (*n* = 5), CKD/ApoE^−/−^ (*n* = 6), CKD/cGAS^−/−^/ApoE^−/−^ (*n* = 5), and CKD/Sting^−/−^/ApoE^−/−^ (*n* = 5) mice. E) The MFIs of p‐TBK1 and p‐IRF3 in arterial VSMCs of healthy people and CKD patients (*n* = 10). F) The percentage of SA*β*G^+^ VSMCs, as well as, the MFIs of IFN*β* and *α*‐SMA in VSMCs from Sham, Sham/Tagln‐Sting, CKD, CKD/Tagln‐Sting mice (*n* = 6). Data represent mean ± SD. *p < 0.05, **p < 0.01, two‐tailed Student's *t*‐test was applied to (B,E); one‐way ANOVA was applied to (D,F).

IFN‐I induction is mainly mediated by the innate immune response. Recently, an innate immune pathway responsible for cytosolic DNA sensing, cGAS‐STING pathway, has been shown to be activated in tissue inflammation and aging.^[^ ^22^ ^]^ Interestingly, the expressions of typical genes involved in cGAS‐STING pathway could be readily detected in VSMCs, comparable to the expressions in myeloid cells (**Figure** **4**A), indicating that VSMCs were both IFN‐I‐responsive and IFN‐I‐productive. Moreover, significantly increased phosphorylation of TANK‐binding kinase 1 (TBK1) and IRF3, the key kinases of the cGAS‐STING pathway, was detected in the aortas and fibrous cap VSMCs of CKD/ApoE^−/−^ mice (Figure 4B,C). Similar changes were observed in the mouse aortas, human arteries, and VSMCs under CKD condition (Figure 4E; Figure S9, Supporting Information). Conversely, knockout or knockdown of cGAS and STING, the key molecules in the cGAS‐STING pathway, significantly attenuated CKD‐induced IFN‐I response, premature senescence, and phenotypic switching in VSMCs (Figure 4D; Figure S10, Supporting Information). To further evaluate the specific role of cGAS‐STING pathway activation in VSMCs, we created a CKD mouse model using a VSMC‐specific knockout of Sting (CKD/Tagln‐Sting) mice. We found that IFN‐I response, premature senescence, and phenotypic switching in VSMCs were significantly attenuated in CKD/Tagln‐Sting mice (Figure 4F). Above all, these data suggested that cGAS‐STING pathway‐dependent IFN‐I response in VSMCs might promote VSMC senescence and phenotypic switching in an autocrine/paracrine manner.

### 2.5 Defect in the Cyclic GMP‐AMP Synthase‐Stimulator of Interferon Genes Pathway or Type‐I‐Interferon Signaling Mitigates Atherosclerosis Progression and Plaque Vulnerability

**Figure 5:**
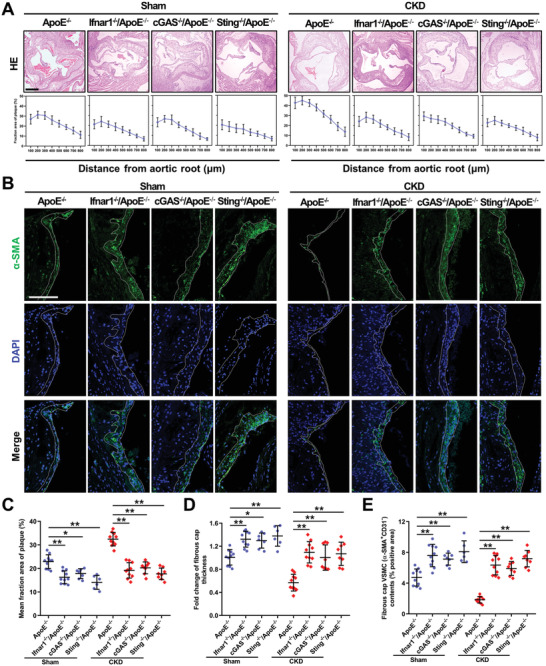
Defect in the cGAS‐STING pathway or IFN‐I signaling mitigates AS progression and plaque vulnerability. A) Representative images of HE and the fraction area of multiple sections of aortic root plaques of Sham/ApoE^−/−^ (*n* = 10), Sham/Ifnar1^−/−^/ApoE^−/−^ (*n* = 10), Sham/cGAS^−/−^/ApoE^−/−^ (*n* = 7), Sham/Sting^−/−^/ApoE^−/−^ (*n* = 6), CKD/ApoE^−/−^ (*n* = 10), CKD/Ifnar1^−/−^/ApoE^−/−^ (*n* = 10), CKD/cGAS^−/−^/ApoE^−/−^ (*n* = 8), and CKD/Sting^−/−^/ApoE^−/−^ (*n* = 8). B) Representative images of *α*‐SMA staining in aortic root plaques of Sham/ApoE^−/−^, Sham/Ifnar1^−/−^/ApoE^−/−^, Sham/cGAS^−/−^/ApoE^−/−^, Sham/Sting^−/−^/ApoE^−/−^, CKD/ApoE^−/−^, CKD/Ifnar1^−/−^/ApoE^−/−^, CKD/cGAS^−/−^/ApoE^−/−^, and Sting^−/−^/CKD/ApoE^−/−^ mice. Scale bar (HE), 200 µm. Scale bar (IF), 100 µm. C–E) The mean fraction area of plaque, fold change of fibrous cap thickness and fibrous cap VSMC contents in the aortic root of Sham/ApoE^−/−^ (*n* = 10), Sham/Ifnar1^−/−^/ApoE^−/−^ (*n* = 10), Sham/cGAS^−/−^/ApoE^−/−^ (*n* = 7), Sham/Sting^−/−^/ApoE^−/−^ (*n* = 6), CKD/ApoE^−/−^ (*n* = 10), CKD/Ifnar1^−/−^/ApoE^−/−^ (*n* = 10), CKD/cGAS^−/−^/ApoE^−/−^ (*n* = 8), and CKD/Sting^−/−^/ApoE^−/−^ (*n* = 8). Data represent mean ± SD. *p < 0.05, **p < 0.01, one‐way ANOVA.

To investigate whether IFN‐I response was involved in VSMC loss and plaque vulnerability, we crossed cGAS^−/−^, Sting^−/−^, and Ifnar1^−/‐^ mice in an ApoE^−/‐^ background. The defect of IFN‐I response significantly attenuated AS progression and plaque vulnerability in CKD/ApoE^−/−^ mice (**Figure** **5**). Interestingly, Sham/ApoE^−/−^ mice also displayed reduced plaque areas and increased plaque stability, manifested by increased VSMC contents and thick fibrous cap (Figure 5). These results suggested that IFN‐I response‐induced pathological changes in VSMCs might be an important contributor to plaque vulnerability and AS progression in mice, especially in CKD/ApoE^−/−^ mice.

### 2.6 Mitochondrial Damage‐Induced Mitochondrial DNA Release is Central to Chronic Kidney Disease‐Induced Type‐I‐Interferon Response in Vascular Smooth Muscle Cells

**Figure 6:**
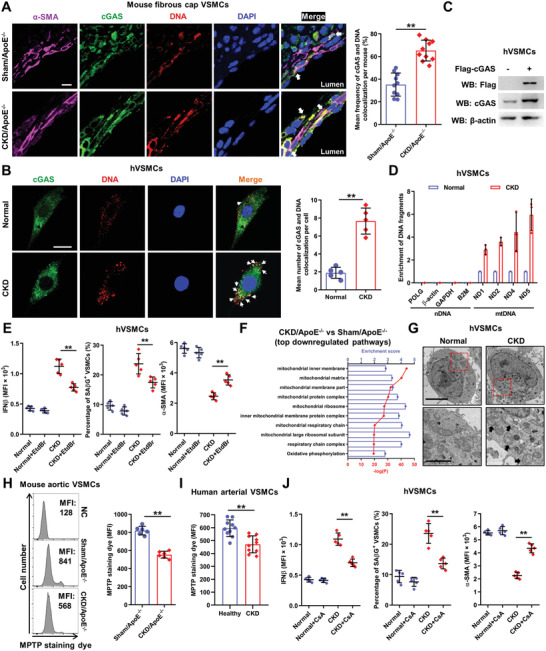
Mitochondrial damage‐induced mitochondrial DNA (mtDNA) release is central to CKD‐induced IFN‐I response in VSMCs. A) Representative images and quantification of cGAS and DNA colocalization in fibrous cap VSMCs of Sham/ApoE^−/−^ and CKD/ApoE^−/−^ mice (*n* = 10 mice). The arrow indicates cGAS and DNA colocalization in fibrous cap VSMCs. Scale bar, 10 µm. B) Representative images and quantification of cGAS and DNA colocalization in hVSMCs incubated with normal or CKD serum (*n* = 5 independent experiments). The arrow indicates cGAS and DNA colocalization. Scale bar, 10 µm. C) Representative WB analysis of hVSMCs transduced with an expression lentivirus encoding Flag‐cGAS. D) Relative enrichment of DNA fragments as indicated using an anti‐Flag antibody to coprecipitate DNA in hVSMCs incubated with normal or CKD serum (*n* = 3). E) The percentage of SA*β*G^+^ hVSMCs, as well as, the MFIs of IFN*β* and *α*‐SMA in hVSMCs incubated with normal or CKD serum after EtdBr pretreatment (*n* = 5). F) KEGG and GO enrichment analysis of top downregulated pathways in aortic VSMCs of CKD/ApoE^−/−^ mice based on the microarray data. G) Representative TEM images of mitochondria in hVSMCs incubated with normal or CKD serum. The box indicates the region magnified in the down panels. The arrow indicates the defect mitochondria. Scale bar (upper panels), 5 µm. Scale bar (down panels), 2 µm. H) Representative FC analysis and quantification of MPTP permeability in aortic VSMCs of Sham/ApoE^−/−^ and CKD/ApoE^−/−^ mice (*n* = 6). I) MPTP permeability in arterial VSMCs of healthy people and CKD patients (*n* = 10). J) The percentage of SA*β*G^+^ hVSMCs, as well as, the MFIs of IFN*β* and *α*‐SMA in hVSMCs incubated with normal or CKD serum after CsA pretreatment (*n* = 5). **p < 0.01, two‐tailed Student's *t*‐test was applied to (A,B,H,I); one‐way ANOVA was applied to (E,J). Oligo, oligomycin; FCCP, fluoro‐carbonyl cyanide phenylhydrazone; Rot, rotenone; Ant, antimycin A.

DNA released from nuclear or mitochondria is the main source of endogenous DNA that activates cGAS‐STING pathway.^[^ ^22^ ^]^ We observed obvious colocalization of DNA with cGAS in the fibrous cap VSMCs of Sham/ApoE^−/−^ mice and the colocalization was much more frequent in CKD/ApoE^−/−^ mice (**Figure** **6**A). Moreover, increased colocalization of DNA with cGAS was confirmed in CKD serum‐incubated hVSMCs in vitro (Figure 6B). Next, we sought to identify the DNA source responsible for the activation of the cGAS‐STING pathway. We first immunoprecipitated Flag‐tagged cGAS in CKD serum‐incubated hVSMCs to detect coprecipitated DNA fragments, and observed significant enrichment for mitochondrial DNA (mtDNA), such as NADH dehydrogenases (ND1, ND2, ND4, ND5), but not nuclear DNA (nDNA), including DNA polymerase gamma, *β*‐actin, glyceraldehyde‐3‐phosphate dehydrogenase, and *β*2 microglobulin (B2M) (Figure 6C,D). To further verify this finding, we depleted mtDNA but not nDNA using ethidium bromide (EtdBr). EtdBr pretreatment induced drastic reduction in mtDNA without affecting the nDNA and significantly inhibited CKD‐induced IFN‐I response, premature senescence, and phenotypic switching in hVSMCs (Figure 6E; Figure S11A,B, Supporting Information). Together, these data suggested that endogenous mtDNA release might be responsible for cGAS‐STING activation, and subsequent IFN‐I response in VSMCs.

mtDNA release is a marker of mitochondrial damage that was well‐established in AS pathogenesis.^[^ ^23^ ^]^ Consistently, KEGG and GO enrichment analysis revealed robust downregulation of genes associated with mitochondrial structure and function in VSMCs from CKD/ApoE^−/−^ mice (Figure 6F), suggesting that enhanced mitochondrial damage in VSMCs might be responsible for accelerated AS progression and plaque vulnerability. Furthermore, quantitative proteomics confirmed enhanced mitochondrial damage in endothelium‐removed aortas of CKD/ApoE^−/−^ mice (Figure S11C,D, Supporting Information). Meanwhile, mitochondrial damage, manifested by swelling and vacuolation, was frequently observed in fibrous cap VSMCs of CKD/ApoE^−/−^ mice (Figure 2G), as well as in CKD serum‐incubated hVSMCs (Figure 6G). Concordantly, functional analysis revealed remarkable mitochondrial dysfunction in CKD serum‐incubated hVSMCs, including declined aerobic respiration capacity, mitochondrial membrane potential, and ATP production (Figure S11E–H, Supporting Information).

Mitochondrial dysfunction may favor the opening of mitochondrial permeability transition pore (MPTP) and lead to increased mitochondrial permeability and the release of mitochondrial factors including mtDNA into the cytosol.^[^ ^23^ ^]^ Indeed, significantly increased mitochondrial permeability was detected in the VSMCs of CKD/ApoE^−/−^ mice (Figure 6H). CKD patients‐derived arteries and CKD serum‐incubated hVSMCs also exhibited increased MPTP permeability (Figure 6I; Figure S11I, Supporting Information), while pretreatment with cyclosporin a (CsA), a potent inhibitor of MPTP, significantly abrogated CKD‐induced IFN‐I response, premature senescence, and phenotypic switching in hVSMCs (Figure 6J; Figure S11J, Supporting Information). These data indicated that endogenous mtDNA release resulting from mitochondrial damage might be central to IFN‐I response in VSMCs of CKD/ApoE^−/−^ mice.

### 2.7 Oxidative Stress Plays a Key Role in Chronic Kidney Disease‐Induced Mitochondrial Damage and Type‐I‐Interferon Response in Vascular Smooth Muscle Cells

**Figure 7:**
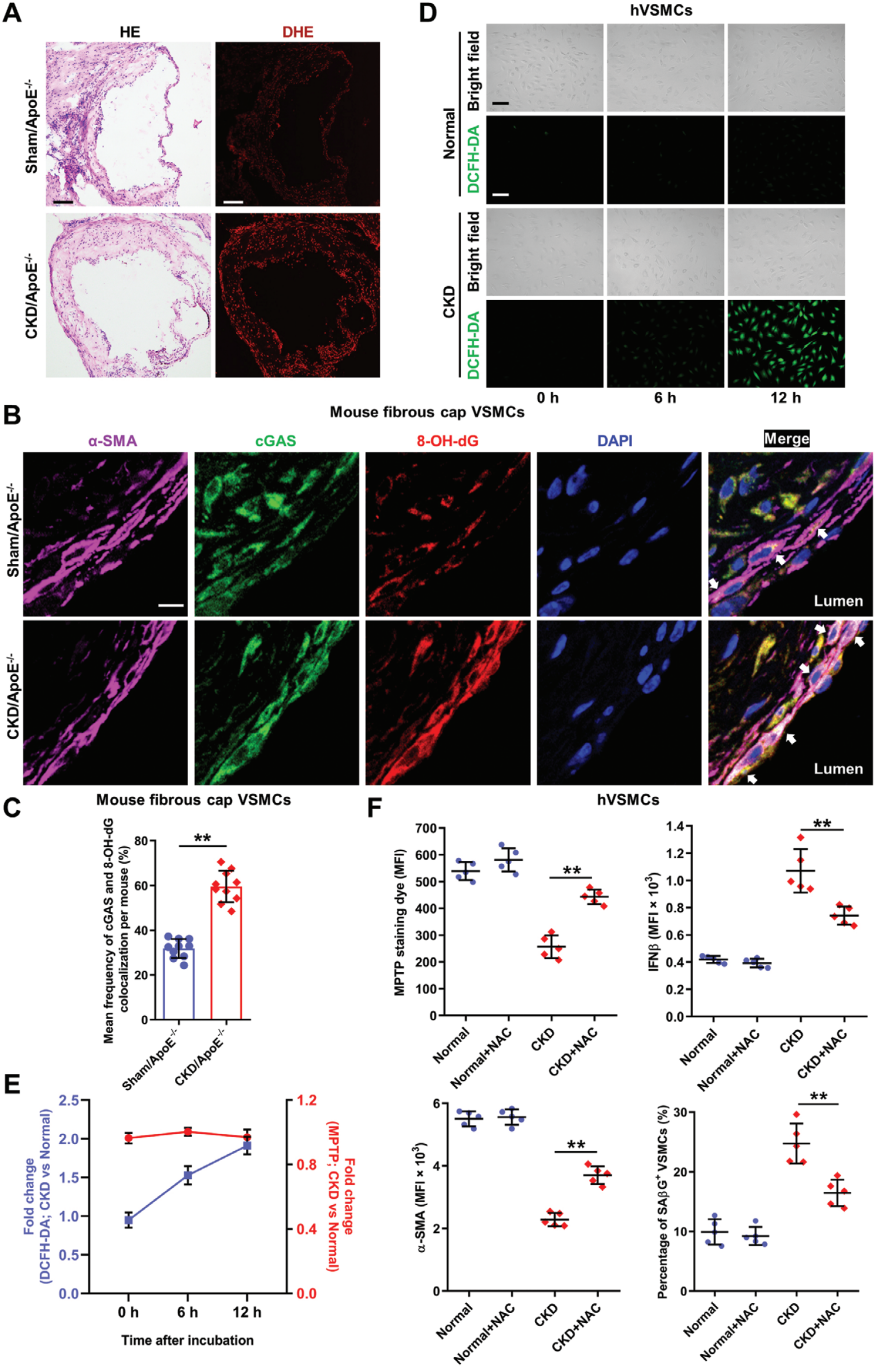
Oxidative stress plays a key role in CKD‐induced mitochondrial damage and IFN‐I response in VSMCs. A) Representative images of HE and dihydroethidium (DHE) staining in aortic root plaques of Sham/ApoE^−/−^ and CKD/ApoE^−/−^ mice. Scale bar (HE), 100 µm. Scale bar (DHE), 100 µm. B) Representative images of cGAS and 8‐hydroxy‐2'‐deoxyguanosine (8‐OH‐dG) colocalization in fibrous cap VSMCs of Sham/ApoE^−/−^ and CKD/ApoE^−/−^ mice. The arrow indicates cGAS and 8‐OH‐dG colocalization in fibrous cap VSMCs. Scale bar, 10 µm. C) Frequency of cGAS and 8‐OH‐dG colocalization in the fibrous cap VSMCs of Sham/ApoE^−/−^ and CKD/ApoE^−/−^ mice (*n* = 10 mice). D) Representative images of 2', 7'‐dichlorodihydrofluorescein diacetate (DCFH‐DA) staining in hVSMCs incubated with normal or CKD serum at indicated time. Scale bar, 50 µm. E) Fold changes of the MFIs of DCFH‐DA and MPTP in CKD serum‐incubated hVSMCs after normalization to the Normal group (*n* = 5). F) The MPTP permeability in hVSMCs, the percentage of SA*β*G^+^ hVSMCs, as well as, the MFIs of IFN*β* and *α*‐SMA in hVSMCs incubated with normal or CKD serum after NAC pretreatment (*n* = 5). **p < 0.01, two‐tailed Student's *t*‐test was applied to (C); one‐way ANOVA was applied to (F).

Mitochondrion is sensitive to oxidative stress and persistent mitochondrial damage is often coupled with chronic reactive oxygen species (ROS) exposure found in age‐related diseases including AS and CKD.^[^ ^24^ ^]^ As expected, plaque ROS levels were markedly increased in CKD/ApoE^−/−^ mice (**Figure** **7**A). Meanwhile, oxidative DNA was frequently observed and colocalized with cGAS in the fibrous cap VSMCs of AS and CKD/ApoE^−/−^ mice (Figure 7B,C). In vitro, ROS production was immediately and significantly increased in CKD serum‐incubated hVSMCs, preceding the appearance of mitochondrial damage (Figure 7D,E). Similarly, CKD serum‐incubated hVSMCs also exhibited obvious colocalization of oxidative DNA with cGAS (Figure S12A,B, Supporting Information), further confirming oxidative damage to mitochondria. Scavenging ROS by an ROS scavenger, N‐acetylcysteine (NAC), significantly alleviated CKD‐induced mitochondrial damage and subsequent IFN‐I response, premature senescence, and phenotypic switching in hVSMCs (Figure 7F; Figure S12C,D, Supporting Information). Therefore, oxidative stress played a key role in mitochondrial damage and IFN‐I response in VSMCs during the AS progression.

### 2.8 Restraining Type‐I‐Interferon Response is a Potential Therapeutic Avenue for Mitigating Atherosclerosis Progression and Plaque Vulnerability

**Figure 8:**
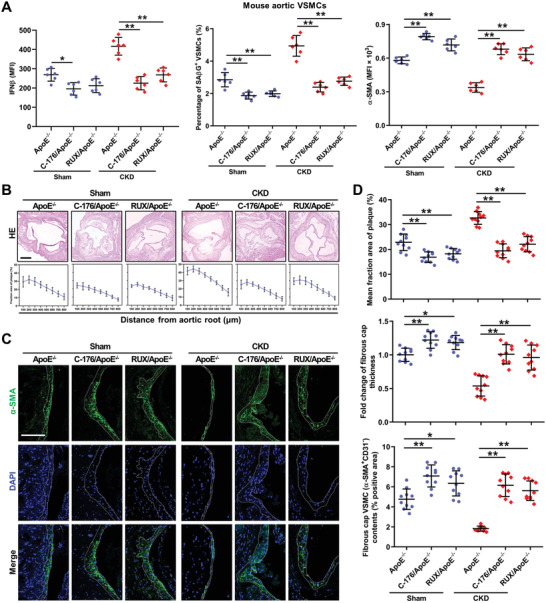
Restraining IFN‐I response is a potential therapeutic avenue for mitigating AS progression and plaque vulnerability. A) The percentage of SA*β*G^+^ VSMCs, as well as, the MFIs of IFN*β* and *α*‐SMA in aortic VSMCs from Sham/ApoE^−/−^ and CKD/ApoE^−/−^ mice after C‐176 or RUX treatment (*n* = 6). B) Representative images of HE and the fraction area of multiple sections of aortic root plaques of Sham/ApoE^−/−^ and CKD/ApoE^−/−^ mice after C‐176 or RUX treatment (*n* = 10). Scale bar, 200 µm. C) Representative images of *α*‐SMA staining in aortic root plaques of Sham/ApoE^−/−^ and CKD/ApoE^−/−^ mice treated with or without C‐176 or RUX. Scale bar, 100 µm. D) The mean fraction area of plaque, fold change of fibrous cap thickness, and fibrous cap VSMC contents in the aortic root of Sham/ApoE^−/−^ and CKD/ApoE^−/−^ mice after C‐176 or RUX treatment (*n* = 10).

Finally, we evaluated the translational potential of our study in treating CKD‐associated plaque vulnerability using newly identified STING inhibitors, C‐176 and its derivate H‐151^[^ ^25^ ^]^ or a JAK‐STAT inhibitor, ruxolitinib (RUX). Pretreatment of hVSMCs with H‐151 or RUX significantly inhibited CKD serum‐induced IFN‐I response, premature senescence, and phenotypic switching in VSMCs (Figure S13A–C, Supporting Information). Mitigation of IFN‐I response by C‐176 or RUX treatment also remarkably alleviated VSMC premature senescence and phenotypic switching both in Sham/ApoE^−/−^ and CKD/ApoE^−/−^ mice (**Figure** **8**A). Finally, C‐176 or RUX treatment significantly retarded AS progression and improved plaque vulnerability both in Sham/ApoE^−/−^ and CKD/ApoE^−/−^ mice (Figure 8B–D). Above all, restraining IFN‐I response might represent a promising strategy for the treatment of plaque vulnerability, especially in both in CKD/ApoE^−/−^ mice.

## 3 Discussion

AS has been the leading cause of cardiovascular morbidity and mortality worldwide. Major clinical consequences of AS, such as, myocardial infarction and stroke are mainly associated with thrombotic events caused by the acute rupture of a vulnerable plaque.^[^ ^5^ ^]^ So far, there is still a lack of deep understanding of AS progression and plaque vulnerability. Presently, most of the biological understanding of AS pathogenesis depends on mouse model studies. Although human and AS mouse models share many similarities in plaque development, there are still some limitations impeding a thorough investigation of AS pathogenesis. Particularly, mouse models rarely show evidence of plaque rupture.^[^ ^8^ ^]^ Given that thrombotic events are more likely to occur in CKD patients and CKD/ApoE^−/−^ mice,^[^ ^4^ ^]^ we speculated that the plaques in CKD/ApoE^−/‐^ mice might exhibit some characters of plaque vulnerability. Indeed, after 12 weeks of WD feeding, the plaque and necrotic core were larger, the fibrous cap was thinner, and the incidences of plaque hemorrhage and myocardial infarction were higher in CKD/ApoE^−/‐^ mice, indicating that CKD/ApoE^−/‐^ mouse was a useful mouse model for the investigation of the plaque vulnerability.

It is well‐recognized that inflammation is a key initiator and driver of AS. Infiltrated immune cells and vascular‐resident cells are the main contributors to vascular inflammation.^[^ ^14^ ^]^ Although vascular‐resident cells are the first line of cells against vascular damage, including cholesterol deposition, the mechanism by which they respond to the damage, and initiate and sustain vascular inflammation remains largely unknown. Especially, compared to endothelial cells (ECs), the roles of VSMCs in modulating vascular inflammation are rarely focused upon. In fact, VSMCs are not only responsive cells to inflammatory cytokines, but also actively promote vascular inflammation.^[^ ^26^ ^]^ VSMCs express pattern recognition receptors that mediate innate immunity, such as Toll‐like receptors and Nod‐like receptors.^[^ ^27^, ^28^ ^]^ Once activated, VSMCs secrete multiple inflammatory cytokines. Recently, Luo et al. reported the critical role of an innate immune sensor, STING, in VSMC death and the pathogenesis of aortic aneurysm and dissection.^[^ ^29^ ^]^ In the current study, we further demonstrated a distinct role of the cGAS‐STING pathway in vascular inflammation. We showed that cGAS‐STING pathway was active in VSMCs and could sense released mtDNA in the cytoplasm. Consequently, the enhanced IFN‐I response induced VSMC premature senescence and phenotypic switching in an autocrine/paracrine manner.

Apart from plaque rupture, endothelial erosion is also a main cause of cardiovascular events. Contemporarily, in an era of improved control of traditional risk factors, endothelial erosion appears to account for as high as one‐third of acute coronary syndromes.^[^ ^30^ ^]^ As reported, IFN‐I and the downstream JAK‐STAT1 signaling directly induce EC dysfunction.^[^ ^31^, ^32^ ^]^ Moreover, CKD is closely associated with EC dysfunction.^[^ ^33^ ^]^ In the uremic milieu, IFN‐I response may also induce endothelial erosion and contribute to the increased risk of cardiovascular events in patients with CKD. On the other hand, enhanced IFN‐I response and JAK‐STAT1 signaling activation were reported to be able to promote the recruitment and activation of immune cells including macrophages and T cells as well.^[^ ^34^, ^35^, ^36^ ^]^ The enhanced IFN‐I response in VSMCs may also have the potential to promote macrophage recruitment and proinflammatory activation in plaques. Meanwhile, the proinflammatory activation of macrophages may, in turn, exacerbate IFN‐I response in VSMCs and the consequent loss of fibrous cap VSMCs. Therefore, the harmful effect of enhanced IFN‐I response in VSMCs on the progression of AS may be comprehensive and the alterations of endothelial and macrophage functions may collectively contribute to the pathogenesis of plaque vulnerability.

Mitochondrial damage is observed in all stages of AS and correlates with plaque vulnerability in human.^[^ ^37^ ^]^ However, the causal relation, the cells involved, and the underlying mechanisms still remain elusive. Here, using integrated microarray and proteomics analysis, we revealed that mitochondrial damage was an outstanding event in the VSMCs of CKD/ApoE^−/‐^ mice. As a marker of mitochondrial damage, MPTP opening, which is responsible for mtDNA release, was markedly increased in the VSMCs of CKD/ApoE^−/‐^ mice. mtDNA depletion or MPTP blockade significantly alleviated IFN‐I response, premature senescence and phenotypic switching in VSMCs. Therefore, mitochondrial damage in VSMCs had a causative role in AS progression and plaque vulnerability through priming IFN‐I response, rather than being just a consequence of tissue damage, especially in CKD milieu.

Oxidative stress is closely associated with AS progression and plaque vulnerability.^[^ ^38^ ^]^ Growing evidence demonstrates that various traditional risk factors, including hypercholesteremia, smoking, diabetes mellitus, hypertension, hyperhomocysteinemia, and increased age, can translate into oxidative stress and contribute to atherosclerosis.^[^ ^38^ ^]^ Although it is well‐known that oxidative stress is associated with the enhanced inflammatory burden in AS pathogenesis, the precise mechanisms have yet to be fully elucidated. Mitochondria are highly sensitive to oxidative stress.^[^ ^24^ ^]^ Indeed, we found that oxidative mitochondrial damage was readily observed in VSMCs, while scavenging ROS could reduce mitochondrial damage and subsequent IFN‐I response, indicating that persistent oxidative stress plays a key role in mitochondrial damage and IFN‐I response in VSMCs. Interestingly, inhibition of IFN‐I response also mitigated ROS overproduction and mitochondrial damage in the VSMCs of both Sham/ApoE^−/‐^ and CKD/ApoE^−/‐^ mice (Figure S13D,E, Supporting Information), but to a lesser extent. It is reasonable to presume that a vicious circle of “oxidative stress‐mitochondrial damage‐IFN‐I response” might exist in AS progression and plaque vulnerability. Therefore, our data provide new insights into the causal link between oxidative stress and inflammation.

Besides, as a typical oxidative stress‐associated disease, CKD has emerged as a particularly strong risk factor for CVD.^[^ ^2^ ^]^ Previously, we and others have shown that uremic toxins, especially indoxyl sulfate, *p*‐cresol, indole‐3‐acetic acid, uric acid, and advanced glycosylation end products, are strong pro‐oxidants to various cells, including cardiomyocytes, platelets, and VSMCs.^[^ ^4^, ^39^, ^40^ ^]^ Therefore, the oxidative uremic milieu may aggravate the oxidative burden in plaque VSMCs as well. Moreover, through exacerbating mitochondrial damage‐induced IFN‐I response and the subsequent VSMC premature senescence and phenotypic switching, CKD can synergize with other traditional risk factors to further promote AS progression and plaque vulnerability.

In this study, we found that there were no significant differences in serum cholesterol levels between AS and CKD/ApoE^−/−^ mice, even after genetic invalidation or pharmacological inhibition of IFN‐I response (Figures S10G and S13F, Supporting Information), indicating that abnormal cholesterol metabolism might not be the main contributor to CKD‐associated aggravation of VSMC senescence and phenotypic switching. However, the contributions of other risk factors to VSMC senescence and phenotypic switching cannot be excluded. As reported, defective mitochondrial fission or mitophagy‐induced accumulation of damaged mitochondria and other inflammatory responses can also affect VSMC senescence and phenotypic plasticity.^[^ ^41^, ^42^, ^43^ ^]^ Of note, in our microarray data, we found that other inflammatory cytokines, including tumor necrosis factor and interleukin 1*β*, were also upregulated, but to a lesser extent, in the VSMCs of CKD/ApoE^−/‐^ mice (Figure 3C). Meanwhile, the key molecules related to mitochondrial fission, such as dynamin 1‐like, as well as, mitophagy, such as PTEN‐induced putative kinase protein 1, voltage‐dependent anion channel 1, and sequestosome 1, were also downregulated in the VSMCs of CKD/ApoE^−/‐^ mice (GEO accession GSE135626). The distinct influence of the alterations of these factors on VSMCs deserves further research. Nevertheless, these findings reflect that IFN‐I response may play an important, but not exclusive, role in inducing VSMC senescence and phenotypic switching in CKD milieu.

## 4 Conclusion

In summary, our data demonstrate that mtDNA release resulting from oxidative stress‐induced mitochondrial damage activates the cGAS‐STING pathway and subsequently induces IFN‐I response in VSMCs. Through promoting VSMC premature senescence and phenotypic switching, IFN‐I response in VSMCs accelerates AS progression and promotes plaque vulnerability. Caution should be taken for patients with CVD complicated with IFN‐I‐related diseases, such as CKD and virus infection. Conversely, mitigation of IFN‐I response may represent a promising avenue for treating AS and plaque vulnerability, especially, CKD‐associated CVD.

## 5 Experimental Section

##### Animals

C57BL/6N‐Mb21d1^tm1cyagen^ (cGAS^−/−^), C57BL/6N‐Tmem173^tm1cyagen^ (Sting^−/−^), and C57BL/6N‐Ifnar1^tm1cyagen^ (Ifnar1^−/−^) mice were created using CRISPR/Cas9 technique by Cyagen Biosciences (Guangzhou, China) and gene knockout in aortas of each mouse was verified using western blot (Figures S8A and S10A, Supporting Information). B6;129‐Tmem173^tm1(flox)Smoc^ (Sting^loxp/loxp^; C57BL/6 background) mice were purchased from Shanghai Model Organisms Center, Inc. (Shanghai, China). B6.129P2‐apoE^tm1Unc^/J (ApoE^−/−^; C57BL/6 background) mice and B6.Cg‐Tg(Tagln‐cre)1Her/J (Tagln‐Cre; C57BL/6 background) mice were purchased from The Jackson Laboratory (Bar Harbor, ME, USA). The atherosclerosis susceptibility of C57BL/6J and C57BL/6N strains is comparable as reported.^[^ ^44^ ^]^ To avoid the potential differences in atherosclerosis susceptibility between the two sub‐strains of C57BL/6 mice, CRISPR/Cas9 mice were crossed onto the ApoE^−/−^ background for more than six generations as described (Figure S8B, Supporting Information). The control and experimental littermates were used for the comparison of plaque morphology. The CKD/ApoE^−/−^ mouse model was created as previously described.^[^ ^4^ ^]^ Briefly, male ApoE^−/−^ mice at 8 weeks of age were first inflicted with 2/3 electrocoagulation of the right renal cortex and then received left total nephrectomy 2 weeks later. Normal C57BL/6 mice were purchased from the Institute of Zoology (Chinese Academy of Sciences, Beijing, China). ApoE^−/−^ mice were fed a high‐cholesterol WD according to the formula of D12108C (Research Diets Inc., New Brunswick, NJ, USA). For RUX (MedChemExpress, Monmouth Junction, NJ, USA) treatment, each mouse was daily fed a small amount of atherogenic food (0.5 g) mixed with RUX (60 mg kg^−1^ body weight), in addition to regular atherogenic diet. During the 12 weeks of treatment, all mice consumed the drug‐containing food completely every day. C‐176 (MedChemExpress) was reconstituted in DMSO at 10 mg mL^−1^ and diluted in 5% Tween‐80 (Sigma‐Aldrich, St. Louis, MO, USA) and 5% PEG‐400 (Sangon Biotech, Shanghai, China). For C‐176 administration, mice were treated with a dose of 4 mg kg^−1^ intraperitoneally every other day. Serum levels of total cholesterol, LDL/VLDL cholesterol and HDL cholesterol were measured using an HDL and LDL/VLDL cholesterol Assay Kit (Abcam, Cambrige, England, UK) according to the manufacturer's instructions. All mice used were male, age‐matched, and syngeneic in the C57BL/6 background. All mice used were housed in specific pathogen‐free conditions and fed with autoclaved food. All the experimental procedures were approved by the Animal Care Committee of the Army Medical University (Third Military Medical University).

##### Human Samples

Uremic serum and healthy serum were collected from CKD stage 5 patients undergoing conservative treatment and Physical Examination Department of Xinqiao Hospital for sex‐ and age‐matched subjects, respectively. 10 mL whole blood was collected and serum was separated by centrifuging at 1000 × *g* for 10 min at room temperature. The exclusion criteria included inflammatory and autoimmune associated‐disease, hypohepatia, polycystic kidney disease, renal cancer, and diabetes. Blood urea nitrogen, creatinine, and glucose were assayed in all patients, and normal sera were excluded if creatinine was >1.0 mg dL^−1^. Human arteries were collected from healthy people donating a kidney and CKD patients undergoing a renal transplant in Department of Urology of Xinqiao Hospital and Center of Urology of Southwest Hospital and informed consent was obtained. The study protocol was approved by the Ethics Committee of Xinqiao Hospital, and the study was carried out in accordance with the Declaration of Helsinki.

##### Cell Culture

hVSMCs were purchased from the American Type Culture Collection (ATCC; Rockville, MD, USA). Cells were cultured in 5% CO_2_/37 °C incubator and grown with Dulbecco's modified Eagle's medium (DMEM; Gibco, Grand Island, NY, USA) supplemented with 10% fetal bovine serum (FBS; Gibco) and 1% penicillin/ streptomycin. Cells from passage 2–5 were used for experiments. Addition of 0.1% FBS to DMEM was used as starvation medium. To deplete mtDNA, hVSMCs were pretreated with 150 ng mL^−1^ EtdBr (Sigma‐Aldrich) for 4 days and mtDNA copy numbers were determined by quantitative PCR (qPCR) as described below. To inhibit MPTP opening, hVSMCs were pretreated with 5 µM CsA (MedChemExpress) for 30 min. To scavenge ROS, hVSMCs were pretreated with 5 mm NAC (Sigma‐Aldrich) for 1 h. To inhibit STING or JAK‐STAT, hVSMCs were pretreated with 1 µM H‐151 or 1 µM RUX (both MedChemExpress) for 1 h, respectively.

For primary mVSMC culture, mouse aortas were dissected and ECs were removed manually using a cotton bud. The endothelium‐removed aortas from 1 to 10 mice were pooled and cut into pieces of about 1–2 mm. Single‐cell suspension was obtained using a mixed enzymatic digestion solution containing 450 U mL^−1^ Collagenase I, 250 U mL^−1^ Collagenase XI, 120 U mL^−1^ Hyaluronidase (all Sigma‐Aldrich), and 120 U mL^−1^ DNase I (Roche, Indianapolis, IN, USA) for 90 min at 37 °C. Then, single‐cell suspension of primary mVSMCs was obtained by filtering through a 70‐µm cell strainer and cultured with DMEM supplemented with 10% FBS and 1% penicillin/streptomycin in 24‐well plate. For IFN*β* treatment, hVSMCs or primary mVSMCs were incubated with indicated concentrations of recombinant human IFN*β* (PeproTech, Rocky Hill, NJ, USA) or recombinant mouse IFN*β* (R&D Systems, Minneapolis, MN, USA) for 3 days.

##### Migration Assay

For ex vivo VSMC migration, mouse thoracic aortas from Sham/ApoE^−/−^ and CKD/ApoE^−/−^ mice were stripped of endothelium and adventitia, and the media of the vessel wall was dissected into 1 × 1‐mm fragments. The media fragments were then suspended within Matrigel Matrix High Concentration, Phenol‐Red Free, LDEV‐free (Corning Inc., Corning, NY, USA), and cultured for 7 days in DMEM supplemented with 10% autologous mouse sera. Migratory activity was quantified by measuring the mean distance migrated by the leading front of VSMCs from the media fragments from 5 independent experiments.

##### Flow Cytometry and Cell Sorting

Single‐cell suspension of mVSMCs was prepared as described above. The gating strategy is outlined in Figure S2A, Supporting Information. Briefly, dead cells were excluded using DAPI (Biolegend, San Diego, CA, USA) staining. For VSMC (CD31^−^CD45^−^Ter‐119^−^) analysis and sorting, monoclonal antibodies against CD31, CD45, and Ter‐119 (all eBioscience, San Diego, CA, USA) were used to exclude ECs, leukocytes, and erythrocytes. Further, the purity of mVSMCs was confirmed by *α*‐SMA staining. For macrophage (CD45^+^ CD11b^+^CD68^+^) sorting, monoclonal antibodies against CD45, CD11b, and CD68 (all eBioscience) were used. Single‐cell suspension of VSMCs from human arteries was obtained as described above. For VSMC (CD31^−^CD45^−^CD235a^−^) analysis, monoclonal antibodies against CD31, CD45, and CD235a (all eBioscience) were used to exclude ECs, leukocytes and erythrocytes. To detect the cytoplasmic protein expression, pre‐stained mVSMCs, human arterial VSMCs or hVSMCs were first fixed with IC Fixation buffer (eBioscience) at room temperature for 20 min and then permeabilized with Permeabilization buffer (eBioscience) in the presence of anti‐IFN*β*, anti‐*α*‐SMA, anti‐p16, or anti‐MX1 (all Abcam, Cambridge, UK) antibodies at room temperature for another 30 min. Finally, the cells were incubated with fluorescent dye conjugated secondary antibodies (Thermo Fisher Scientific, Carlsbad, CA, USA) for 30 min and analyzed by FC. To detect the nuclear protein expression, pre‐stained mVSMCs, human arterial VSMCs, or hVSMCs were resuspended with 1 mL Foxp3 fixation/permeabilization working solution (eBioscience) and incubated at room temperature for 30 min. Then, the cells were permeabilized with permeabilization buffer (eBioscience) in the presence of anti‐Ki‐67 (eBioscience) or anti‐p53 (Cell Signaling Technology, Danvers, MA, USA) antibodies at room temperature for another 30 min. Finally, the cells were stained with fluorescent dye conjugated secondary antibodies (Thermo Fisher Scientific) and analyzed by FC. To detect the expression of intracellular phosphorylated protein, pre‐stained mVSMCs, human arterial VSMCs, or hVSMCs were first fixed with IC Fixation buffer (eBioscience) at room temperature for 20 min. Then, the cells were resuspended with 1 mL ice‐cold methanol and incubated on ice for 30 min. After carefully washing, the cells were resuspended with Flow Cytometry Staining Buffer (eBioscience) in the presence of anti‐p‐TBK1, anti‐p‐IRF3 (all Cell Signaling Technology), or anti‐p‐STAT1 (Biolegend) for another 30 min at room temperature, followed by FC analysis.

Apoptosis analysis was performed using an Annexin V‐FITC Apoptosis Detection Kit (eBioscience). Single‐cell suspension of hVSMCs was resuspended in suitable volume of 1 × Binding Buffer. Then, Annexin V‐FITC antibody was added and incubated for 10 min at room temperature. After washing with 1 × Binding Buffer, 7‐amino‐actinomycin D staining solution (eBioscience) was added and the cells were immediately analyzed by FC.

Mouse innate immune cells including monocytes/macrophages and neutrophils were sorted using a myeloid marker (CD11b). Briefly, for CD11b^+^ cell sorting, mouse bone marrow cells were flushed from the femur and tibia. Red blood cells were lysed using red cell lysis buffer. Monoclonal antibody against CD11b (eBioscience) was used to label mouse myeloid cells. Human innate immune cells (monocytes/macrophages) were sorted using a myeloid marker (CD14). Briefly, for CD14^+^ cell sorting, human peripheral blood mononuclear cells were isolated from blood of healthy donors by density gradient centrifugation using Histopaque‐1077 (Sigma‐Aldrich). Then, CD14^+^ cells were isolated using an EasySep CD14^+^ Positive Selection Kit (StemCell Technologies, Vancouver, BC, Canada) following the manufacturer's instructions.

Cells were sorted using an FACSAria III (BD Biosciences, San Jose, CA, USA) or analyzed using an FACSverse (BD Biosciences) flow cytometer. Data analysis was performed using FlowJo software (Treestar Inc., San Carlos, CA, USA). The detailed information of antibodies used in FC is listed in Table S3, Supporting Information.

##### Microarray

RNA was extracted from sorted mVSMCs using an RNeasy Micro Kit (QIAGEN, Hilden, Germany). RNA quality and quantity were examined by mulitimager and Agilent 4200 TapeStation System. cDNA was synthesized using a cDNA synthesis kit (Affymetrix, Inc., Santa Clara, CA, USA) and labeled using a GeneChip WT PLUS Reagent Kit (Affymetrix, Inc.). Then, the labeled cDNA was hybridized to the mouse Gene chip at 45 °C for 16 h. After washing with wash solution A, wash solution B, and deionized water, the gene chip was stained with Cocktail 1 and Cocktail 2. The GeneChip Scanner 3000 7G (Affymetrix Inc.) and the AGCC Scan Control software (Affymetrix Inc.) were used for date analysis. GSEA was performed using Molecular Signatures Database v6.0 (http://software.broadinstitute.org/gsea/msigdb). Microarray hybridization was carried out by Shanghai GMINIX Biotech Limited Company (Shanghai, China) using the GeneChip Mouse Gene 1.0 ST Array (Affymetrix Inc.). The microarray data was deposited in the NCBI Gene Expression Omnibus under accession number GSE135626.

##### Tandem Mass Tag/Isobaric Tags for Relative and Absolute Quantitation Quantitative Proteomics

Protein extraction of endothelium‐removed aortas was conducted as described below. The protein solution was reduced with 5 mm dithiothreitol for 30 min at 56 °C and alkylated with 11 mm iodoacetamide for 15 min at room temperature in darkness. The protein sample was diluted by adding 100 mm TEAB to urea concentration less than 2 m. Then, trypsin was added at 1:50 trypsin‐to‐protein mass ratio for the first digestion overnight and 1:100 trypsin‐to‐protein mass ratio for a second 4‐h digestion. After trypsin digestion, peptides were desalted by Strata X C18 SPE column (Phenomenex, Torrance, CA, USA) and vacuum‐dried. Peptides were reconstituted in 0.5 m TEAB and processed by a tandem mass tag kit/isobaric tags for relative and absolute quantitation kit according to the manufacturer's instructions. Subsequently, the tryptic peptides were fractionated by high pH reverse‐phase high performance liquid chromatography using Betasil C18 column (5 µm particles, 10 mm ID, 250 mm length; Thermo Hypersil‐Keystone, Bellefonte, PA, USA). Finally, the tryptic peptides were analyzed by Liquid chromatography‐tandem mass spectrometry (LC‐MS/MS). The resulting MS/MS data were processed using Maxquant search engine v.1.5.2.8 (www.maxquant.org). Tandem mass spectra were searched against human Uniprot database (http://www.ebi.ac.uk/uniprot/) concatenated with reverse decoy database. The proteomics data are listed in Table S4, Supporting Information.

##### Senescence Analysis

SA*β*G activity in mVSMCs, human arterial VSMCs and hVSMCs was quantified by FC using an ImaGene Green C_12_FDG lacZ gene expression kit (Thermo Fisher Scientific). Pre‐stained mVSMCs or hVSMCs were re‐suspended in pre‐warmed (37 °C) C_12_FDG working solution at a final concentration of 33 µM and incubated for 30 min at 37 °C. Then, the cells were washed and analyzed by FC. SA*β*G staining kit (Cell Signaling Technology) was used to detect SA*β*G activity of hVSMCs. SA*β*G^+^ hVSMCs were photographed using a microscope (Olympus Optical, Tokyo, Japan) and quantified using ImageJ software. The mean frequency of SA*β*G^+^ VSMCs was calculated from 20 fields of each independent experiment.

##### Mitochondrial Permeability Transition Pore Assay

The MPTP opening was determined by FC using an MPTP assay kit (Biovision, Mountain View, CA, USA) according to the manufacturer's instructions. Briefly, pre‐stained mVSMCs, human arterial VSMCs or hVSMCs were resuspended in pre‐warmed MPTP Wash Buffer at a final concentration of 1 × 10^6^ cells mL^−1^. Then, MPTP Staining Dye was added and the cells were incubated at 37 °C for 15 min at the presence of cobalt chloride. After washing, the cells were resuspended in MPTP Wash Buffer and analyzed by FC.

##### Mitochondrial Membrane Potential Analysis

Mitochondrial membrane potential of hVSMCs was determined by FC using a MitoProbe JC‐1 Assay Kit (Thermo Fisher Scientific) according to the manufacturer's instructions. Briefly, hVSMCs were trypsinized, resuspended, and incubated in 2 µM JC‐1 staining solution for 20 min at 37 °C. After washing once with PBS, the cells were resuspended in 500 µL PBS and analyzed immediately by FC.

##### Plaque and Intracellular Reactive Oxygen Species Analysis

Fresh frozen sections of aortic root were incubated with 10 µM DHE (Thermo Fisher Scientific) in a humidified chamber protected from light for 0.5 h at 37 °C, and then photographed under a fluorescence microscope (Olympus Optical). Meanwhile, the adjacent sections of serial sections were stained for HE. hVSMCs were incubated with 10 µM DCFH‐DA (Sigma‐Aldrich) in a humidified chamber protected from light for 0.5 h at 37 °C, and then immediately photographed under a fluorescence microscope (Olympus Optical).

##### ATP Measurement

ATP levels were measured using an Enhanced ATP Assay Kit (Beyotime, Shanghai, China) according to the manufacturer's instructions. First, hVSMCs incubated with normal or CKD serum were rinsed and lysed using ATP lysis buffer on ice. Samples were centrifuged at 12 000 rpm for 10 min at 4 °C to acquire supernatant for further determination. Then, ATP standard curve was prepared, samples and ATP detection working dilution were added, and luminescence was measured using a Tecan Infinite 200 PRO microplate reader. The concentration of ATP was calculated according to the ATP standard curve. Finally, the intracellular ATP content was normalized by the protein concentration in each sample lysed by ATP lysis buffer.

##### Seahorse Assay

The Seahorse XFp mitochondrial stress test was performed using an Agilent Seahorse XFp Cell Mito Stress Test Kit (Agilent Technologies, Palo Alto, CA, USA) according to the manufacturer's instructions. Briefly, hVSMCs incubated with normal or CKD serum were seeded at a density of 1 × 10^4^ cells per well on XFp microplates. Then, the cells were rinsed once and XF assay medium was added. After three measurements of baseline OCR, respiratory chain inhibitors including 1 µM Oligo, 1 µM FCCP and 0.5 µM Rot/Ant were sequentially injected into each well. Three OCR readings were taken after the addition of each inhibitor and before the automated injection of the subsequent inhibitor. OCR was automatically calculated by Wave software version 2.4.0 (Agilent Technologies) and normalized by cell numbers.

##### WB Analysis

Endothelium‐removed aortas and cells were homogenized in RIPA buffer supplemented with cOmplete EDTA‐free Protease Inhibitor Cocktail and PhosSTOP Phosphatase Inhibitor Cocktail (all Roche) for 30 min at 4 °C, and then centrifuged at 10 000 × *g* for 15 min at 4 °C. The protein concentrations were determined by a BCA protein concentration determination kit (Thermo Fisher Scientific). The proteins were separated by sodium dodecyl sulfate polyacrylamide gel electrophoresis. Protein expression levels were detected using anti‐p53, anti‐p‐STAT1, anti‐STAT1, anti‐STING, anti‐IRF3, anti‐p‐TBK1, anti‐p‐IRF3 (all Cell Signaling Technology), anti‐p16, anti‐*β*‐actin, anti‐IFN*β*, anti‐MX1, anti‐*α*‐SMA, anti‐MYH11, anti‐CNN1, anti‐TAGLN, anti‐TBK1 (all Abcam), anti‐IRF7, anti‐IFNΑR1 (Novus Biologicals, Littleton, CO, USA), and anti‐cGAS (Santa Cruz Biotechnology, Santa Cruz, CA, USA) antibodies at 4 °C overnight. Finally, membranes were incubated with appropriate secondary antibodies (Abcam) and scanned by a Bio‐Rad ChemiDoc MP imager (Bio‐Rad, Hercules, CA, USA). WB bands were quantified using Gel‐Pro analyzer4 software (Media Cybernetics, Inc., Rockville, MD, USA) by measuring the band intensity for each group and normalized to *β*‐actin as an internal control. The expression intensity for each phosphorylated protein was normalized by total protein expression. The detailed information of used antibodies is listed in Table S3, Supporting Information.

##### Enzyme‐Linked Immunosorbent Assay

Endothelium‐removed aortas were homogenized in 200 µL of sterile PBS, and then centrifuged at 5000 × *g* for 5 min at 4 °C. The supernatant was obtained and IFN*β* level was measured in duplicate using a mouse IFN*β* ELISA kit (R&D Systems, Minneapolis, MN, USA) according to the manufacturer's instructions. Serum or medium IFN*β* level was measured in duplicate using a Human Interferon *β* ELISA Kit (Cusabio Biotech, Wuhan, China) according to the manufacturer's instructions. The optical density was measured using a Tecan Infinite 200 PRO microplate reader.

##### Quantitative PCR

RNA of endothelium‐removed aortas, human arteries or hVSMCs was extracted using a TRIzol Plus reagent (TaKaRa, Shiga, Japan). Then, mRNA was treated at 42 °C for 2 min with gDNA Eraser (TaKaRa), reverse‐transcribed into cDNA using a PrimeScript RT reagent Kit (TaKaRa) according to the manufacturer's instructions. The mRNA expression levels of indicated genes were examined using a GoTaq qPCR Master Mix (Promega, Madison, WI, USA) on a CFX96 Real‐Time system (Bio‐Rad). Data was normalized relative to *β*‐actin. The primer sequences used are listed in Table S5, Supporting Information. To quantify mtDNA copy numbers, nDNA and mtDNA from hVSMCs were extracted using a DNeasy Blood and Tissue kit (QIAGEN), and mtDNA copy numbers were quantified by qPCR using a CFX96 Real‐Time system. The level of ND1 (mtDNA) was normalized to the level of B2M (nDNA). The primer sequences used are listed in Table S6, Supporting Information.

##### RNA Interference

Small interfering RNA (siRNA) was purchased from Genepharma (Shanghai, China). hVSMCs were seeded until 50% confluence and were transfected with siRNA (Genepharma, Shanghai, China) for IFNAR1, STAT1, cGAS, and STING (siIFNAR1, siSTAT1, sicGAS and siSTING). Lipofectamine 2000 (Thermo Fisher Scientific) was used according to the manufacturer's recommended instructions. The expression levels of IFNAR1, STAT1, cGAS, and STING were examined by WB and qPCR after 48 h. 48 h after transfection, cells were starved for 24 h and then incubated with normal or CKD serum for 3 days. The siRNA sequences used are listed in Table S7, Supporting Information.

##### Flag‐Cyclic GMP‐AMP Synthase Cloning and ChIP

3 × Flag‐tagged human cGAS was sub‐cloned into a pCDH‐CMV‐MCS‐EF1‐GFP+Puro lentiviral vector (Genecreate, Wuhan, China) and the lentivirus was produced by transient transfection of 293T cells with a three‐vector packaging system. Subsequently, stably‐transduced hVSMCs were screened using screening medium containing 0.5 µg mL^−1^ puromycin (MedChemExpress). After treatment with CKD serum for 72 h, transduced hVSMCs were fixed with 1% formaldehyde (Sigma‐Aldrich) for 10 min. After washing three times with ice‐cold PBS, the cells were disrupted by SDS lysis buffer (Millipore, Billerica, MA, USA) and divided into two aliquots. Then, the cross‐linked protein/DNA in one aliquot was immunoprecipitated using anti‐FLAG M2 antibody (Sigma‐Aldrich). The purified DNA from whole‐cell extracts and immunoprecipitated DNA was examined by qPCR using a GoTaq qPCR Master Mix (Promega) on a CFX96 Real‐Time system (Bio‐Rad). The DNA abundance of whole‐cell extracts served as normalization control for the immunoprecipitated DNA abundance. The information of used antibody is listed in Table S3, Supporting Information. Primer sequences for nDNA and mtDNA analysis are listed in Table S6, Supporting Information.

##### Histology and Morphology Analysis of Atherosclerotic Plaques

The atherosclerotic plaques were analyzed as recommended with modifications.^[^ ^45^, ^46^ ^]^ Mouse hearts were perfused and fixed with 4% paraformaldehyde for 24 h, and then dehydrated and embedded with paraffin. The aortic root was sectioned serially in 4‐µm‐thick slices from the aortic valves. The first appearance of aortic valve was set as start, lesion area was quantified from 8 sections at 100 µm intervals per mouse. To analyze plaques in BCA, paraformaldehyde‐fixed, paraffin‐embedded BCA was serially cut in 4‐µm‐thick sections from the aortic arch to the bifurcation of the right subclavian artery. The last appearance of aortic arch was set as start, lesion area was quantified from 5 sections at 100 µm intervals per mouse. The sections were stained with HE and plaque morphology was analyzed using ImagePro Plus software (Media Cybernetics Inc.). The fraction area of plaque was calculated through dividing plaque area by vessel area to avoid the potential errors caused by tilt^4^. The mean fraction area of plaque was calculated from 5–8 serial sections. Plaque hemorrhage was detected using a Movat‐Russell Modified Pentachrome Stain Kit (Solarbio). As reported, aortic plaque is not suitable for detection of plaque hemorrhage due to the interference of breakdown of small intraplaque vessels.^[^ ^8^ ^]^ Therefore, Movat's stain was performed in BCA plaques. Blood components, such as fibrin, were stained intense red.

##### Anatomical and Histological Analysis of Myocardial Infarction

For postmortem examination, hearts were excised and photographed immediately. Myocardial infarction was detected in the dead mice at postmortem examination through anatomical observation. The myocardial infarction area was identified as pale and discolored patches, accompanied by enlarged heart relative to normal heart as reported previously.^[^ ^47^ ^]^ For HE staining, perfused hearts were fixed with 4% paraformaldehyde and embedded with paraffin. The hearts were longitudinally sectioned in 4‐µm‐thick slices. Finally, the sections were stained with HE and photographed under a microscope (Olympus Optical). Myocardial infarction lesion was identified as few remaining cardiomyocytes and numerous mononuclear inflammatory cells. Incidence of myocardial infarction was calculated from anatomical and HE observation as shown in Table S2, Supporting Information.

##### Immunostaining

Frozen sections were sectioned at 4‐µm‐thick in the aortic root and fixed with 4% paraformaldehyde at room temperature for 10 min. Paraffin sections were obtained as described above. For IF staining, both for frozen and paraffin sections, antigen retrieval was achieved by boiling in citrate buffer (Santa Cruz Biotechnology) above 95 °C for 30 min. Then, sections were blocked with goat serum (Santa Cruz Biotechnology) for 40 min at room temperature. For cells, hVSMCs were seeded on coated slides and fixed with 4% paraformaldehyde at room temperature for 10 min. After permeabilization with 0.25% Triton X‐100 (Santa Cruz Biotechnology) for 10 min and blockade with goat serum (Santa Cruz Biotechnology) for 40 min at room temperature, paraffin sections and hVSMCs were stained for p53, p‐STAT1, p‐TBK1, p‐IRF3, *γ*‐H2AX (all Cell Signaling Technology), p16, *α*‐SMA, and IFN*β* (all Abcam) antibodies at 4 °C overnight. Fibrous cap thickness was divided by vessel area from 5–8 serial sections of each mouse. Then, the fold change of fibrous cap thickness was calculated by normalizing to Sham/ApoE^−/−^ group.

To measure fibrous cap VSMC contents, paraffin sections were stained for *α*‐SMA and CD31 (all Abcam) antibodies at 4 °C overnight. As shown in Figure 2A, the specificities of *α*‐SMA and CD31 antibodies are high enough to discriminate VSMCs and ECs. Fibrous cap VSMC (*α*‐SMA^+^CD31^−^) contents were calculated through dividing fibrous cap *α*‐SMA^+^CD31^−^ area by plaque area from 5–8 serial sections of each mouse.

To detect the colocalization of cGAS with DNA/8‐OH‐dG in fibrous cap VSMCs, frozen sections were stained for 8‐OH‐dG (Abcam), *α*‐SMA (Novus Biologicals), cGAS (Abcam), and DNA (Millipore) antibodies and hVSMCs were stained for 8‐OH‐dG (Abcam), cGAS (Santa Cruz Biotechnology) and DNA (Millipore) antibodies at 4 °C overnight. To avoid the interference of DNA staining in nuclei and better show the colocalization of cytoplasmic DNA with cGAS, the sections were permeabilized with 0.1% Triton X‐100 for 10 min. Then, the sections were stained with appropriate fluorescent dye conjugated secondary antibodies. Finally, the sections were counterstained with DAPI and photographed under a Zeiss LSM780 NLO confocal microscope (Carl Zeiss, Jena, Germany). The mean frequency or number of DNA/8‐OH‐dG and cGAS colocalization in fibrous cap per mouse or per cell, were calculated from 8 serial sections of each mouse or 20 fields of independent experiment, respectively.

Double staining for *α*‐SMA and SA*β*G in fibrous cap VSMCs was conducted in frozen sections of aortic root. The sections were fixed in 1 × Fixative solution at room temperature for 10 min. After washing with 1 × PBS three times, the sections were stained with SA*β*G staining solution in a wet incubator without CO_2_ at 37 °C overnight. Subsequently, the sections were stained for *α*‐SMA using immunohistochemistry staining. After antigen retrieval and goat serum blockade, the sections were stained for *α*‐SMA antibody (Abcam) at 4 °C overnight. Then, the sections were stained with horseradish peroxidase labeled goat anti‐rabbit IgG antibody (Santa Cruz Biotechnology) at 37 °C for 30 min. Finally, diaminobenzidine tetrahydrochloride (Santa Cruz Biotechnology) was used to show *α*‐SMA positive area. The sections were photographed under a microscope (Olympus Optical). The mean number of SA*β*G^+^ VSMCs in fibrous cap per mouse was calculated from 8 serial sections of each mouse. To further confirm VSMC senescence in fibrous cap, double staining for CD31 (Abcam), and SA*β*G was conducted in frozen sections of aortic root as described above to exclude the confounding by ECs.

Apoptosis was detected by TUNEL staining in frozen sections of aortic root with an in situ cell death detection kit (Roche). First, the sections were fixed with 4% paraformaldehyde at room temperature for 10 min. After antigen retrieval, the sections were treated with 20 µg mL^−1^ proteinase K (Santa Cruz Biotechnology) for 10 min at 37 °C and stained with TUNEL reaction mixture for 60 min at 37 °C. Finally, the sections were counterstained with DAPI and photographed under a Zeiss LSM780 NLO confocal microscope. The mean number of TUNEL^+^ nuclei in fibrous cap per mouse was calculated from 8 serial sections of each mouse.

##### Transmission Electron Microscopy

Mouse aortas and hVSMCs were collected and fixed in 2.5% glutaraldehyde. Samples were sent to the Biomedical Analysis Center (Army Medical University) for standard transmission electron microscopy ultrastructural analysis. Sections were viewed and photographed using a JEM‐1400 transmission electron microscope (JEOL, Tokyo, Japan). Fibrous cap VSMCs are identified as elongated spindle‐shaped cells or irregularly shaped ramified cells that reside in the subendothelial layer as described previously,^[^ ^17^, ^48^ ^]^ whereas endothelial cells are elongated and adjacent to the lumen and macrophages are highly vacuolated circular cells.

##### Statistical Analysis

Statistical data was analyzed by using Prism 8.0 (GraphPad Software, La Jolla, CA, USA). All results are expressed as mean ± SD. *n* represents mouse numbers or numbers of independent experiments in each experiment, as detailed in figure legends. Comparisons between two groups were determined by two‐tailed Student's *t*‐test, and three or more groups were compared by one‐way ANOVA followed by Tukey‐Kramer post hoc analysis. Serum IFN*β* levels were compared by Mann–Whitney *U* test. Comparison of survival curves was determined by Log‐rank (Mantel‐Cox) test. *p* < 0.05 was considered statistically significant.

## Conflict of Interest

The authors declare no conflict of interest.

## Supporting information

Supporting Information.
