# Supplementary material for: Mitochondrial Damage‐Induced Innate Immune Activation in Vascular Smooth Muscle Cells Promotes Chronic Kidney Disease‐Associated Plaque Vulnerability
Source: Adv Sci (Weinh). 2021 Jan 6;8(5):2002738. doi: 10.1002/advs.202002738 (PMC7927614; doi:10.1002/advs.202002738)
Supplement: Supplementary file 1 — Supporting Information. [file ADVS-8-2002738-s001.pdf]

© 2020 Wiley-VCH GmbH

ADVANCED SCIENCE
Open Access

Supporting Information

for *Adv. Sci.,* DOI: 10.1002/advs.202002738

Mitochondrial damage-induced innate immune activation in vascular smooth muscle cells promotes CKD-associated plaque vulnerability

Xianjin Bi, Changhong Du, Xinmiao Wang, Xue-Yue Wang, Wenhao Han, Yue Wang, Yu Qiao, Yingguo Zhu, Li Ran, Yong Liu, Jiachuan Xiong, Yinghui Huang, Mingying Liu, Chi Liu, Chunyu Zeng, Junping Wang, Ke Yang and Jinghong Zhao**

Supplementary Information

Mitochondrial damage-induced innate immune activation in vascular smooth muscle cells promotes CKD-associated plaque vulnerability

Xianjin Bi, Changhong Du, Xinmiao Wang, Xue-Yue Wang, Wenhao Han, Yue Wang, Yu Qiao, Yingguo Zhu, Li Ran, Yong Liu, Jiachuan Xiong, Yinghui Huang, Mingying Liu, Chi Liu, Chunyu Zeng, Junping Wang, Ke Yang and Jinghong Zhao**

This file includes:

Supplementary Figure S1 to S13

Table S1 to S7

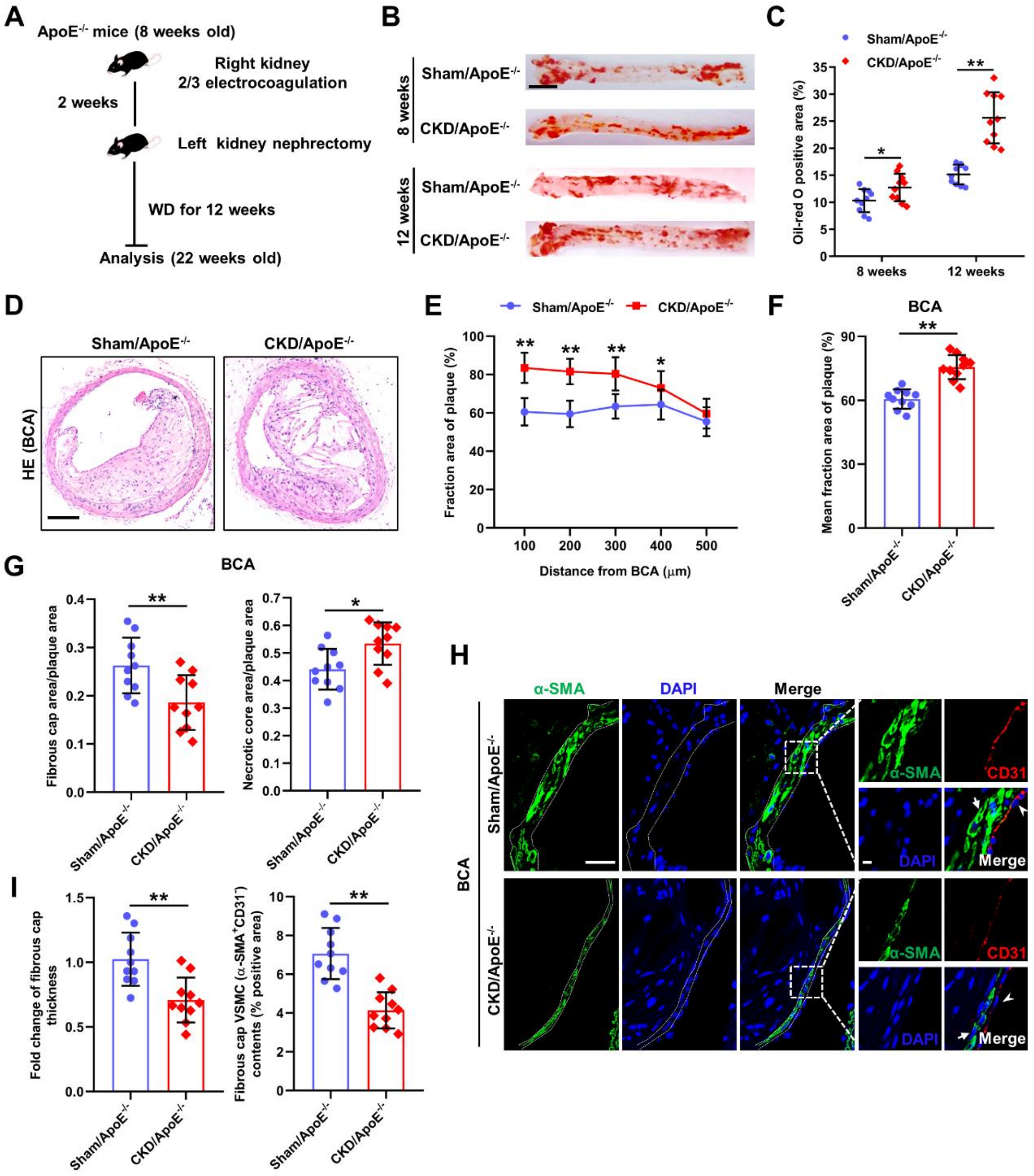

Figure S1. CKD/ApoE$^{-/-}$ mouse is a useful model for investigating the pathogenesis of plaque vulnerability. A) Scheme for CKD mouse model creation. B,C) Representative images and quantification of oil-red O staining in aortas of Sham/ApoE$^{-/-}$ and CKD/ApoE$^{-/-}$ mice after 8 and 12 weeks of WD (*n* = 10). Scale bar, 5 mm. D,E) Representative HE staining and the fraction area of multiple sections in BCA plaques of Sham/ApoE$^{-/-}$ and CKD/ApoE$^{-/-}$ mice (*n* = 10). Scale bar, 100 μm. F) Mean fraction area of BCA plaques in Sham/ApoE$^{-/-}$ and CKD/ApoE$^{-/-}$ mice (*n* = 10). G) Relative areas of fibrous cap and necrotic core in BCA plaques of Sham/ApoE$^{-/-}$ and CKD/ApoE$^{-/-}$ mice (*n* = 10). H,I) Representative images of α-SMA and CD31 staining in BCA plaques and the fold change of fibrous cap thickness and fibrous cap VSMC (α-SMA^{+}CD31^{-}) contents in BCA plaques of Sham/ApoE$^{-/-}$ and CKD/ApoE$^{-/-}$ mice (n = 10). The dashed lines delineate the fibrous cap area. The box indicates the region magnified in the right panels. The arrowhead indicates endothelial cells (α-SMA^{-}CD31^{+}). Scale bar (α-SMA staining), 40 μm. Scale bar (α-SMA and CD31 double staining), 10 μm. Data represent mean ± SD. **P* <0.05, ***P* <0.01, two-tailed Student's *t*-test.

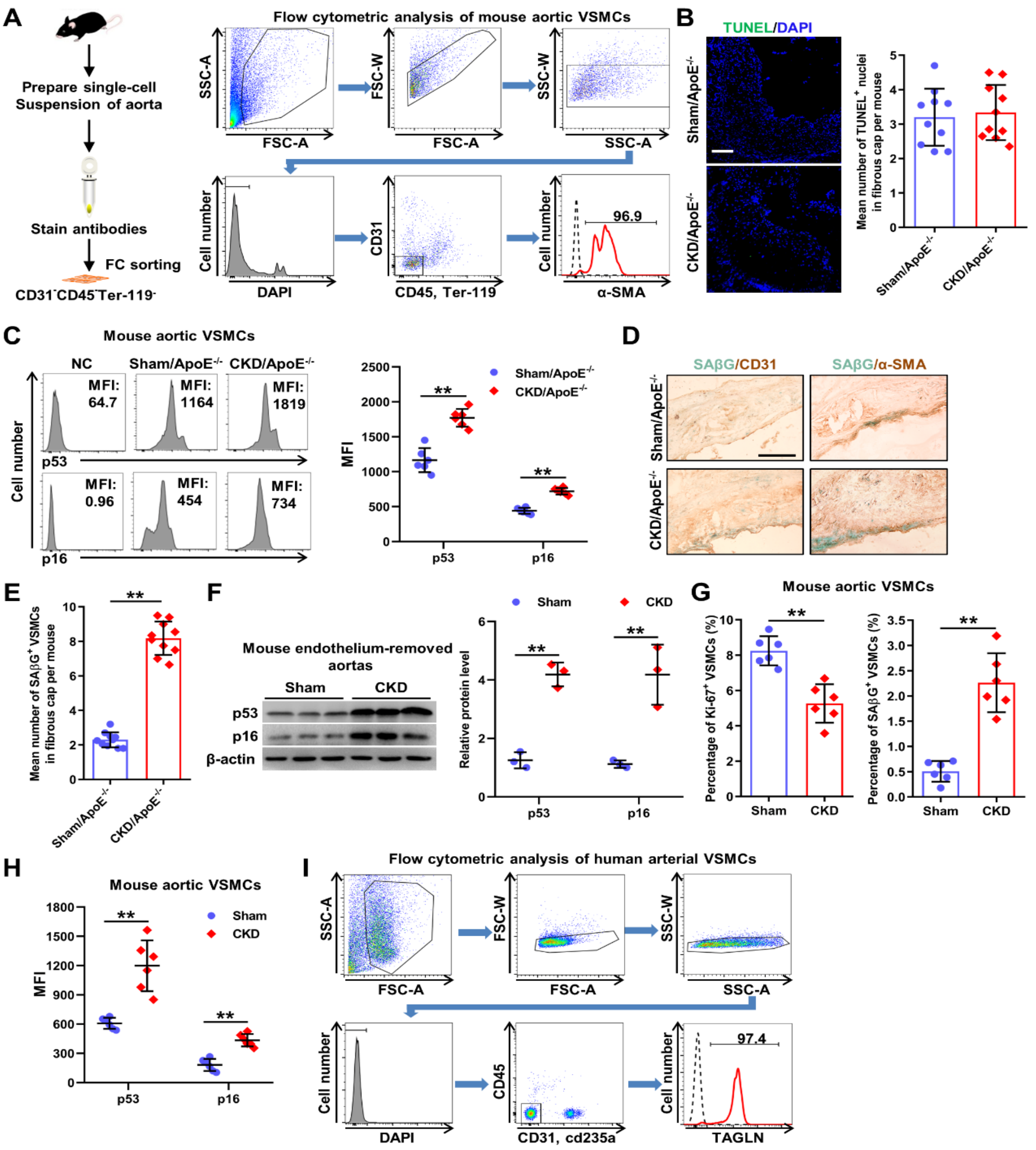

Figure S2. CKD promotes VSMC premature senescence in vivo. A) Scheme for the preparation of single-cell suspension of aorta (left panel) and representative FC isolation and identification of mouse aortic VSMCs (right panels). B) Representative images and quantification of terminal deoxyribonucleotidyl transferase-mediated dUTP-digoxigenin nick end labeling (TUNEL) staining in aortic root plaques of Sham/ApoE$^{-/-}$ and CKD/ApoE$^{-/-}$ mice (n = 10 mice). Scale bar, 200 µm. C) Representative FC analysis, as well as the MFIs of p53 and p16 in aortic VSMCs of Sham/ApoE$^{-/-}$ and CKD/ApoE$^{-/-}$ mice (n = 6). D) Representative images of CD31/α-SMA and SAβG double staining in aortic root plaques of Sham/ApoE$^{-/-}$ and CKD/ApoE$^{-/-}$ mice. These panels show that most of SAβG^{+} cells are beneath the endothelial lining. Scale bar, 100 µm. E) Mean number of SAβG^{+} VSMCs in the fibrous cap of aortic root plaques in Sham/ApoE$^{-/-}$ and CKD/ApoE$^{-/-}$ mice (n = 10 mice). F) WB analysis of p53 and p16 expression levels in endothelium-removed aortas of Sham and CKD mice (n = 3). G,H) The percentages of Ki-67^{+} and SAβG^{+} VSMCs, as well as the MFIs of p53 and p16 in aortic VSMCs of Sham and CKD mice (n = 6). I) Representative FC isolation and identification of human arterial VSMCs. Data represent mean ± SD. **P <0.01, two-tailed Student's t-test.

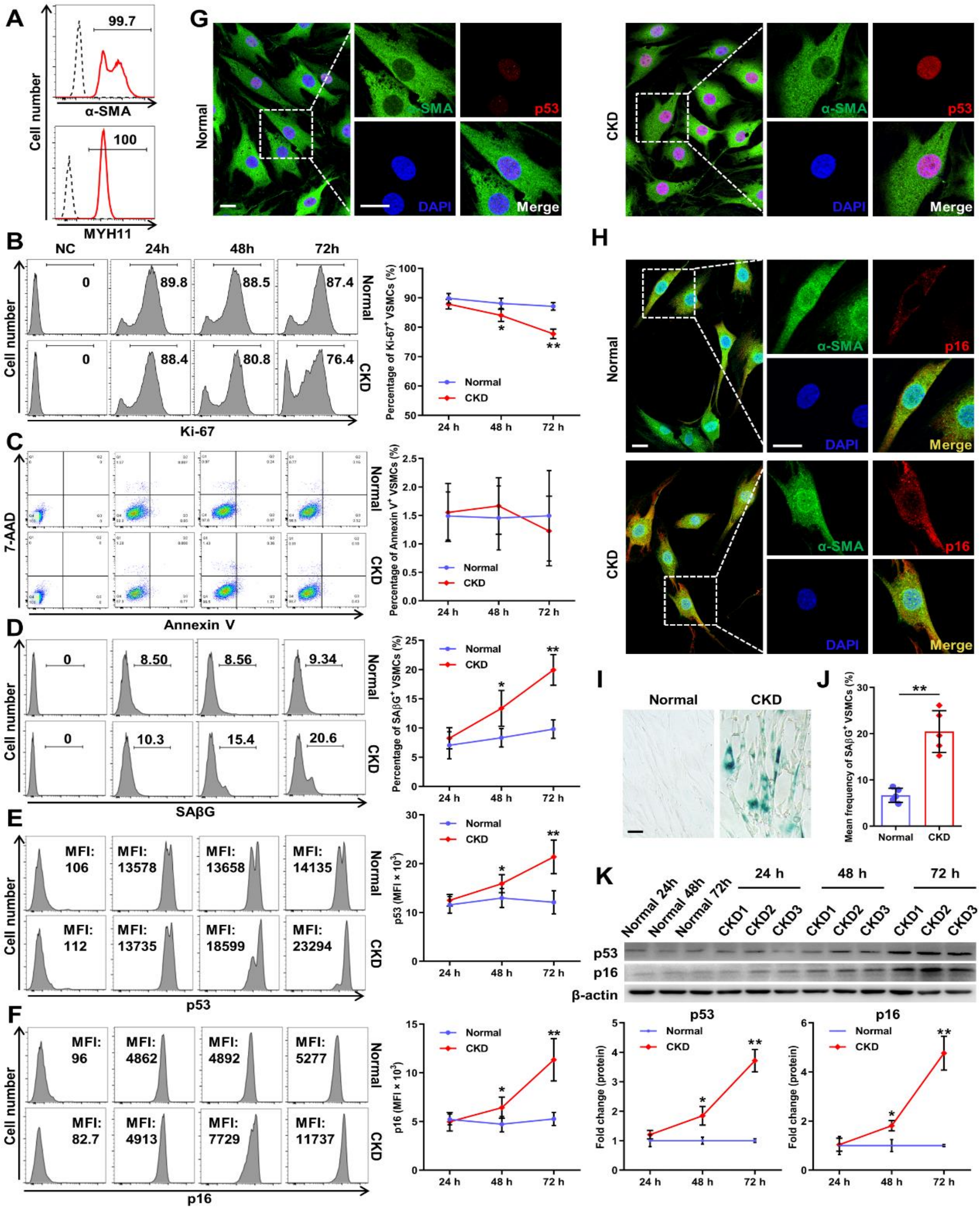

Figure S3. CKD promotes VSMC premature senescence in vitro. A) Representative FC analysis shows that the cultured human VSMCs we used are maturely differentiated VSMCs (α-SMA^{+}, MYH11^{+}). B-F) Representative FC analysis and the percentages of Ki-67^{-}, Annexin V^{+} and SAβG^{+} hVSMCs, as well as the MFIs of p53 and p16 in hVSMCs incubated with normal or CKD serum (*n* = 5). G,H) Representative images of p53 and p16 IF staining in hVSMCs incubated with normal or CKD serum. The box indicates the region magnified in the right panels. Scale bars, 20 μm. I) Representative images of SAβG staining in hVSMCs incubated with normal or CKD serum. Scale bar, 40 μm. J) Mean frequency of SAβG^{+} hVSMCs after incubation with normal or CKD serum (*n* = 5 independent experiments). K) WB analysis of p53 and p16 expression levels in hVSMCs incubated with normal or CKD serum at indicated time (*n* = 3). Data represent mean ± SD. **P* <0.05, ***P* <0.01, two-tailed Student's *t*-test. 7-AAD, 7-amino-actinomycin D.

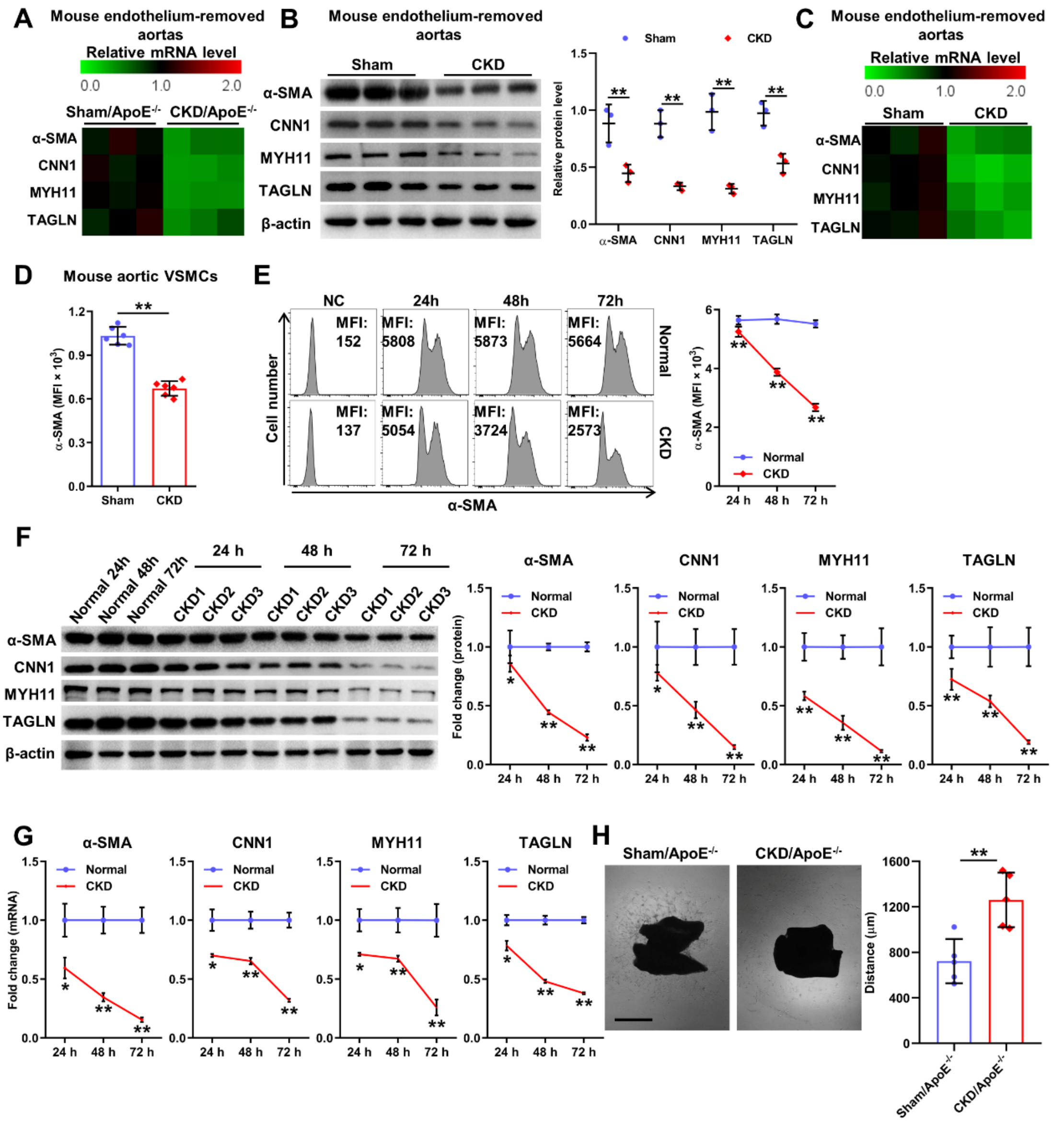

Figure S4. CKD promotes VSMC phenotypic switching both in vivo and in vitro. A) Relative mRNA expression levels of α-SMA, CNN1, MYH11 and TAGLN in endothelium-removed aortas of Sham/ApoE$^{-/-}$ and CKD/ApoE$^{-/-}$ mice. B) WB analysis of α-SMA, CNN1, MYH11 and TAGLN expression levels in endothelium-removed aortas of Sham and CKD mice (*n* = 3). C) Relative mRNA expression levels of α-SMA, CNN1, MYH11 and TAGLN in endothelium-removed aortas of Sham and CKD mice. D) The MFI of α-SMA in aortic VSMCs of Sham and CKD mice (*n* = 6). E) Representative FC analysis and the MFI of α-SMA in hVSMCs incubated with normal or CKD serum (n = 5). F) WB analysis of α-SMA, MYH11, CNN1 and TAGLN expression levels in hVSMCs incubated with normal or CKD serum (n = 3). G) Relative mRNA expression levels of α-SMA, CNN1, MYH11 and TAGLN in hVSMCs incubated with normal or CKD serum (n = 3). H) Representative images and quantification of VSMC migration from the media fragments of Sham/ApoE$^{-/-}$ and CKD/ApoE$^{-/-}$ mice. Data represent mean ± SD. **P* <0.05, ***P* <0.01, two-tailed Student's *t*-test.

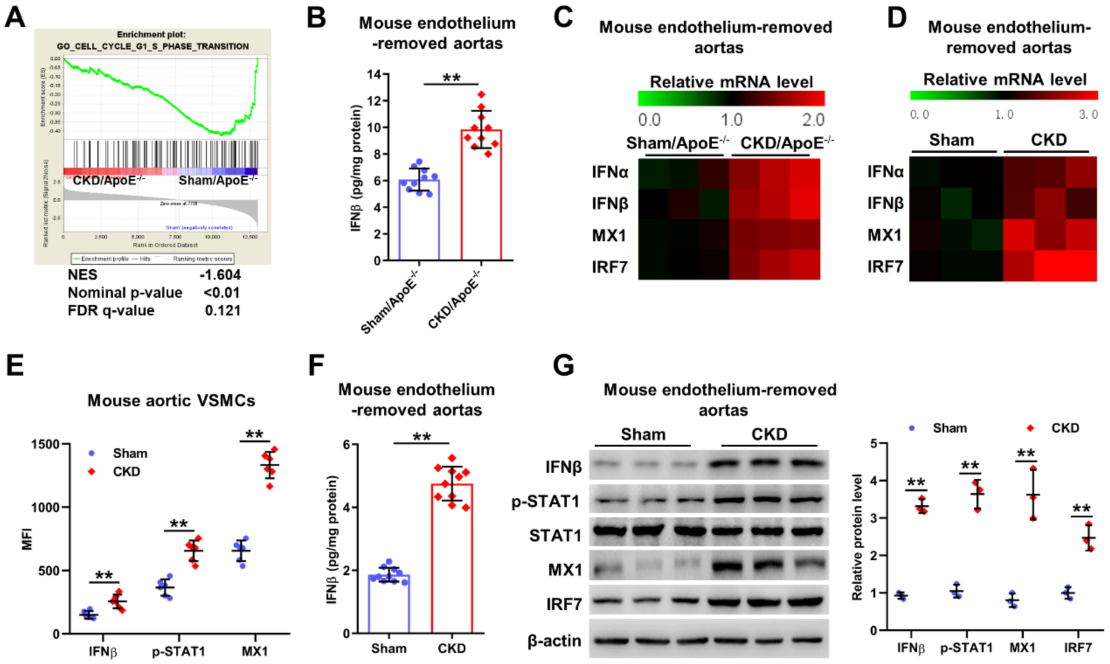

Figure S5. CKD promotes IFN-I signaling activation in VSMCs in vivo. A) GSEA analysis of G1-S transition gene set in aortic VSMCs of Sham/ApoE$^{-/-}$ and CKD/ApoE$^{-/-}$ mice. B) Quantification of IFNβ protein levels by ELISA in endothelium-removed aortas of Sham/ApoE$^{-/-}$ and CKD/ApoE$^{-/-}$ mice (n = 10). C) Relative mRNA expression levels of IFNα, IFNβ, MX1 and IRF7 in endothelium-removed aortas of Sham/ApoE$^{-/-}$ and CKD/ApoE$^{-/-}$ mice. D) Relative mRNA expression levels of IFNα, IFNβ, MX1 and IRF7 in endothelium-removed aortas of Sham and CKD mice. E) The MFIs of IFNβ, p-STAT1 and MX1 in aortic VSMCs of Sham and CKD mice (n = 6). F) Quantification of IFNβ protein levels by ELISA in endothelium-removed aortas of Sham and CKD mice (n = 10). G) WB analysis of IFNβ, p-STAT1, MX1 and IRF7 expression levels in endothelium-removed aortas of Sham and CKD mice (n = 3). Data represent mean ± SD. **P <0.01, two-tailed Student's t-test.

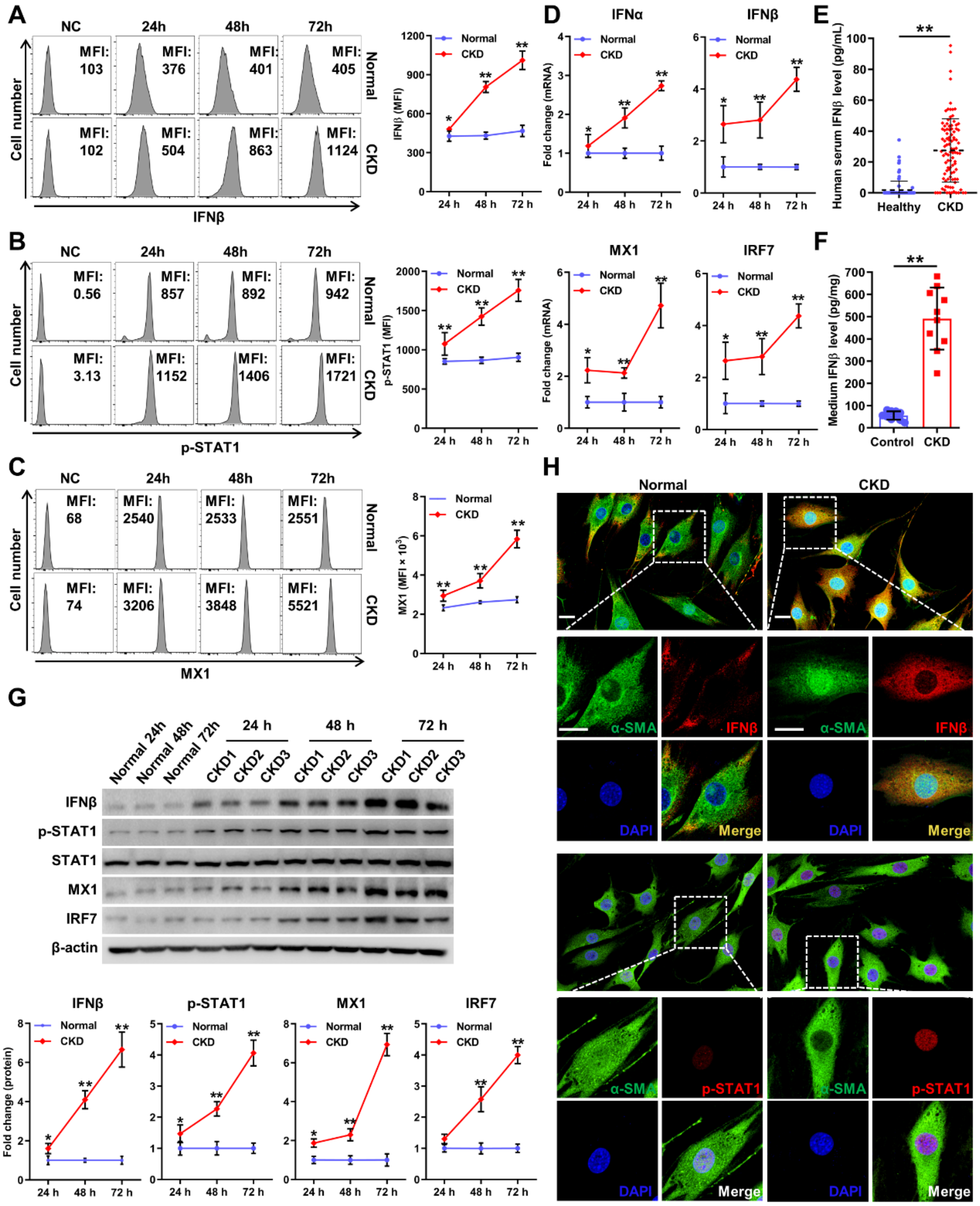

Figure S6. CKD promotes IFN-I signaling activation in VSMCs in vitro in an autocrine/paracrine manner. A-C) The MFIs of IFNβ, p-STAT1 and MX1 in hVSMCs incubated with normal or CKD serum (*n* = 5). D) Relative mRNA expression levels of IFNα, IFNβ, MX1 and IRF7 in hVSMCs incubated with normal or CKD serum (*n* = 3). E) Quantification of the serum IFNβ levels in healthy people (*n* = 92) and CKD patients (*n* = 98) by ELISA. Conversion factor, 1 U = 5 pg. F) Quantification of the medium IFNβ levels of hVSMC culture by ELISA after incubation with normal or CKD serum (*n* = 10). G) WB analysis of IFNβ, p-STAT1, MX1 and IRF7 expression levels in hVSMCs incubated with normal or CKD serum at indicated time (*n* = 3). H) Representative images of IFNβ and p-STAT1 IF staining in hVSMCs incubated with normal or CKD serum. The box indicates the region magnified in the down panels. Scale bars, 20 μm. Data represent mean ± SD. **P* <0.05, ***P* <0.01, two-tailed Student's *t*-test was applied to A-D,F,G; Mann–Whitney U test was applied to E.

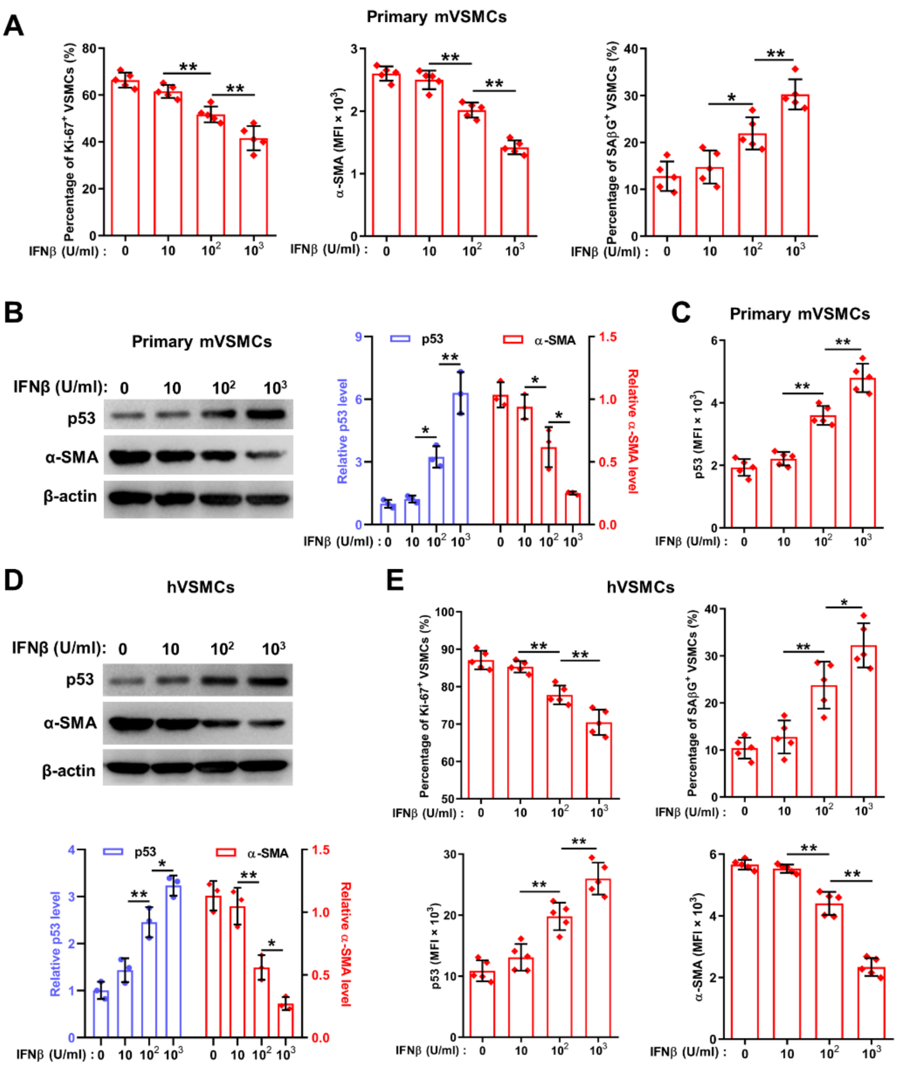

Figure S7. IFN-I promotes VSMC premature senescence and phenotypic switching in both human and mouse VSMCs. A) The percentages of SAβG$^+$ and Ki-67$^+$ cells, as well as the MFI of α-SMA in primary mouse VSMCs (mVSMCs) after treatment with indicated dose of IFNβ(*n* = 5 independent experiments). B) WB analysis of p53 and α-SMA expression levels in primary mouse VSMCs (mVSMCs) after treatment with indicated dose of IFNβ (*n* = 3). C) The MFI of p53 in primary mVSMCs after treatment with indicated dose of IFNβ (*n* = 5 independent experiments). D) WB analysis of p53 and α-SMA expression levels in hVSMCs treated with indicated dose of IFNβ (*n* = 3). E) The percentages of Ki-67$^+$ and SAβG$^+$ cells, as well as the MFIs of p53 and α-SMA in hVSMCs treated with indicated dose of IFNβ (*n* = 5). Data represent mean ± SD. **P* <0.05, ***P* <0.01, one-way ANOVA.

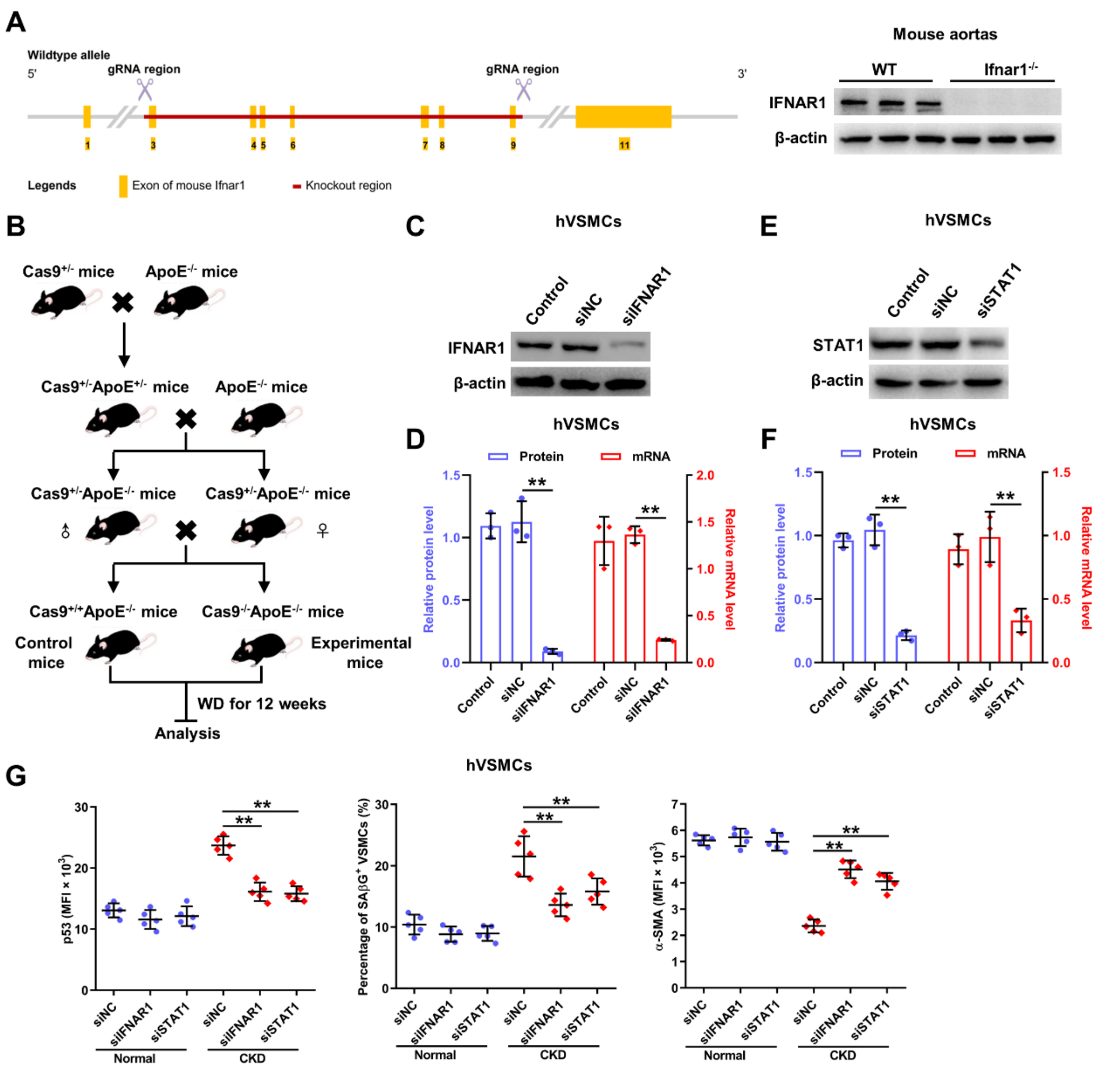

Figure S8. Defect in the IFN-I signaling mitigates CKD-induced VSMC premature senescence and phenotypic switching. A) Scheme for Ifnar1$^{-/-}$ mice creation using CRISPR/Cas9 technique (left panel). IFNAR1 knockout in aortas was verified by WB (right panel). B) Scheme for CRISPR/Cas9 mice and ApoE$^{-/-}$ mice crossing. C-F) Representative WB analysis, as well as the relative protein and mRNA expression levels of IFNAR1 and STAT1 after RNA interference as indicated (n = 3). G) The percentage of SAβG^{+} hVSMCs, as well as the MFIs of p53 and α-SMA in siIFNAR1 and siSTAT1-transfected hVSMCs incubated with normal or CKD serum (n = 5). Data represent mean ± SD. **P <0.01, one-way ANOVA.

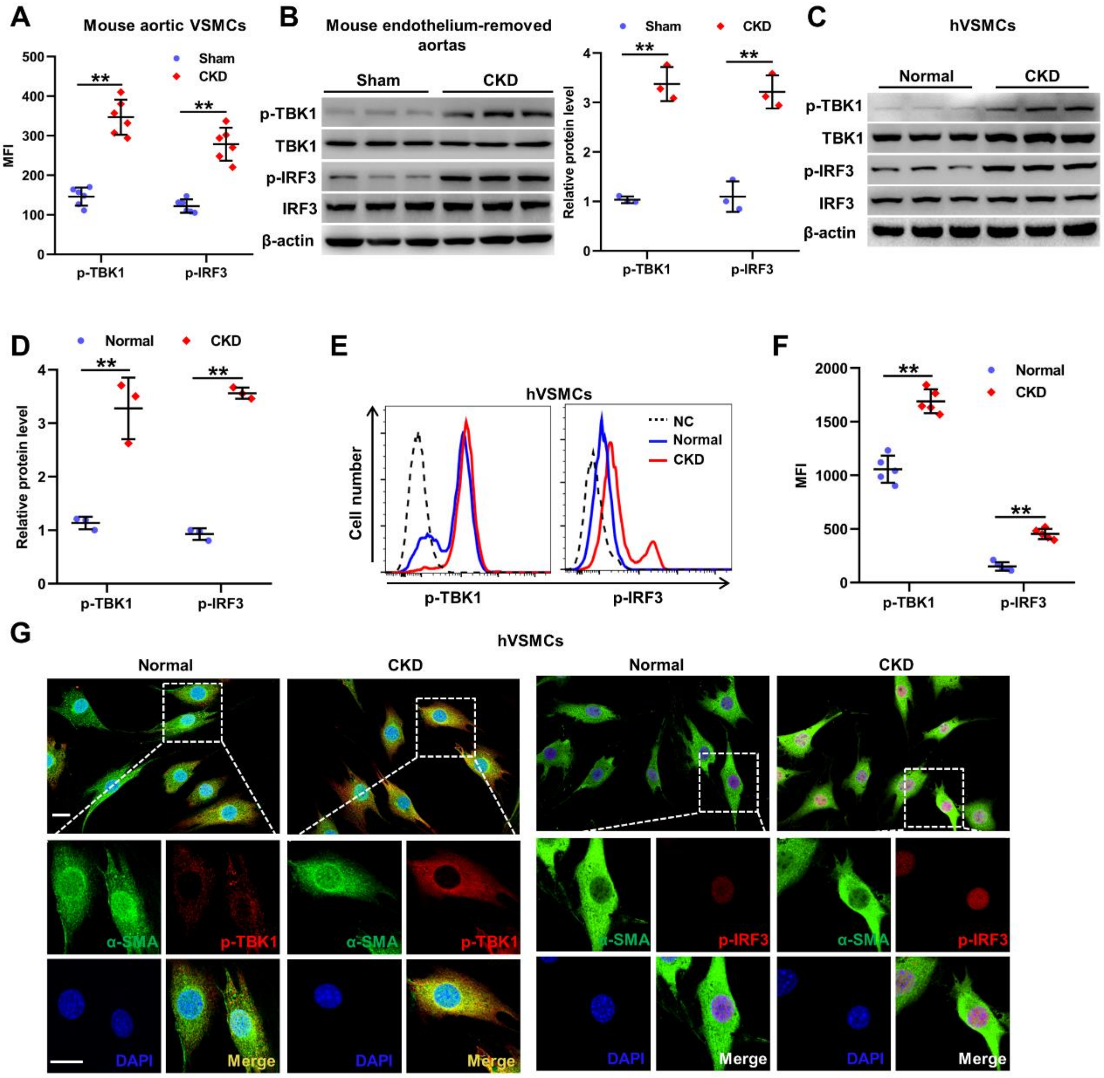

Figure S9. CKD promotes cGAS-STING pathway activation in VSMCs both in vivo and in vitro. A) The MFIs of p-TBK1 and p-IRF3 in aortic VSMCs from Sham and CKD mice (*n* = 6). B) WB analysis of p-TBK1 and p-IRF3 expressions in endothelium-removed aortas from Sham and CKD mice (*n* = 3). C,D) WB analysis of p-TBK1 and p-IRF3 expression levels in hVSMCs incubated with normal or CKD serum (*n* = 3). E,F) The MFIs of p-TBK1 and p-IRF3 in hVSMCs incubated with normal or CKD serum by FC (*n* = 5). G) Representative images of p-TBK1 and p-IRF3 IF staining in hVSMCs incubated with normal or CKD serum. The box indicates the region magnified in the down panels. Scale bars, 20 µm. Data represent mean ± SD. $^{**}P < 0.01$, two-tailed Student's *t*-test.

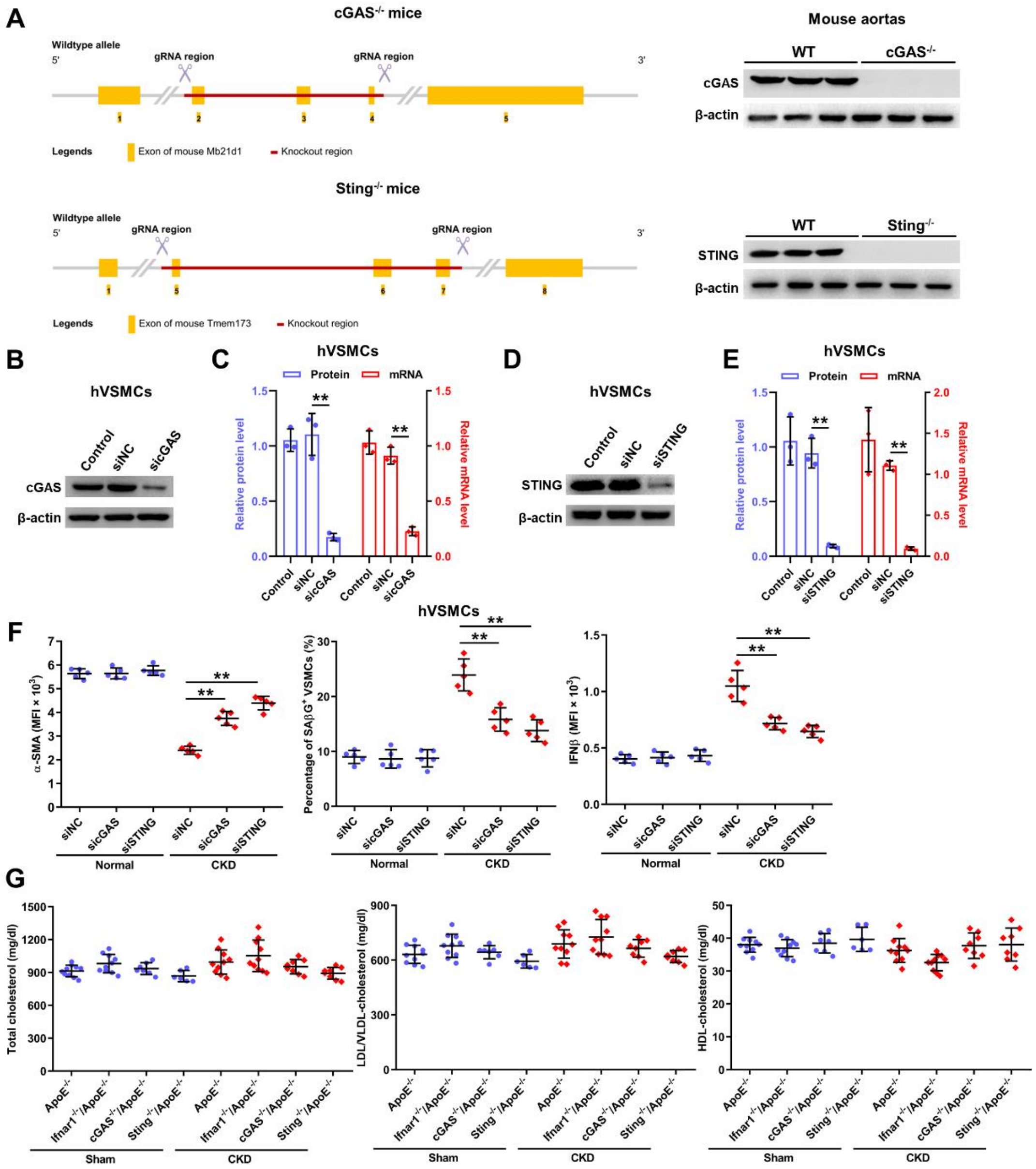

Figure S10. Defect in the cGAS-STING pathway mitigates CKD-induced IFN-I response, premature senescence and phenotypic switching in VSMCs without affecting serum cholesterol levels. A) Scheme for cGAS$^{-/-}$ and Sting$^{-/-}$ mice creation using CRISPR/Cas9 technique (left panels). Gene knockout in aortas for each mouse was verified by WB (right panels). B-E) Representative WB analysis, as well as the relative protein and mRNA expression levels of cGAS and STING in hVSMCs after RNA interference as indicated (*n* = 3). F) The percentage of SAβG^{+} hVSMCs, as well as the MFIs of IFNβ and α-SMA in hVSMCs after RNA interference as indicated (*n* = 5). G) Serum levels of total cholesterol, LDL/VLDL-cholesterol and HDL-cholesterol in Sham/ApoE$^{-/-}$ (*n* = 10), Sham/Ifnar1$^{-/-}$/ ApoE$^{-/-}$ (*n* = 10), Sham/cGAS$^{-/-}$/ ApoE$^{-/-}$ (*n* = 7), Sham/Sting$^{-/-}$/ ApoE$^{-/-}$ (*n* = 6), CKD/ApoE$^{-/-}$ (*n* = 10), CKD/Ifnar1$^{-/-}$/ ApoE$^{-/-}$ (*n* = 10), CKD/cGAS$^{-/-}$/ ApoE$^{-/-}$ (*n* = 8) and CKD/Sting$^{-/-}$/ ApoE$^{-/-}$ (*n* = 8) mice. Data represent mean ± SD. ***P* <0.01, one-way ANOVA.

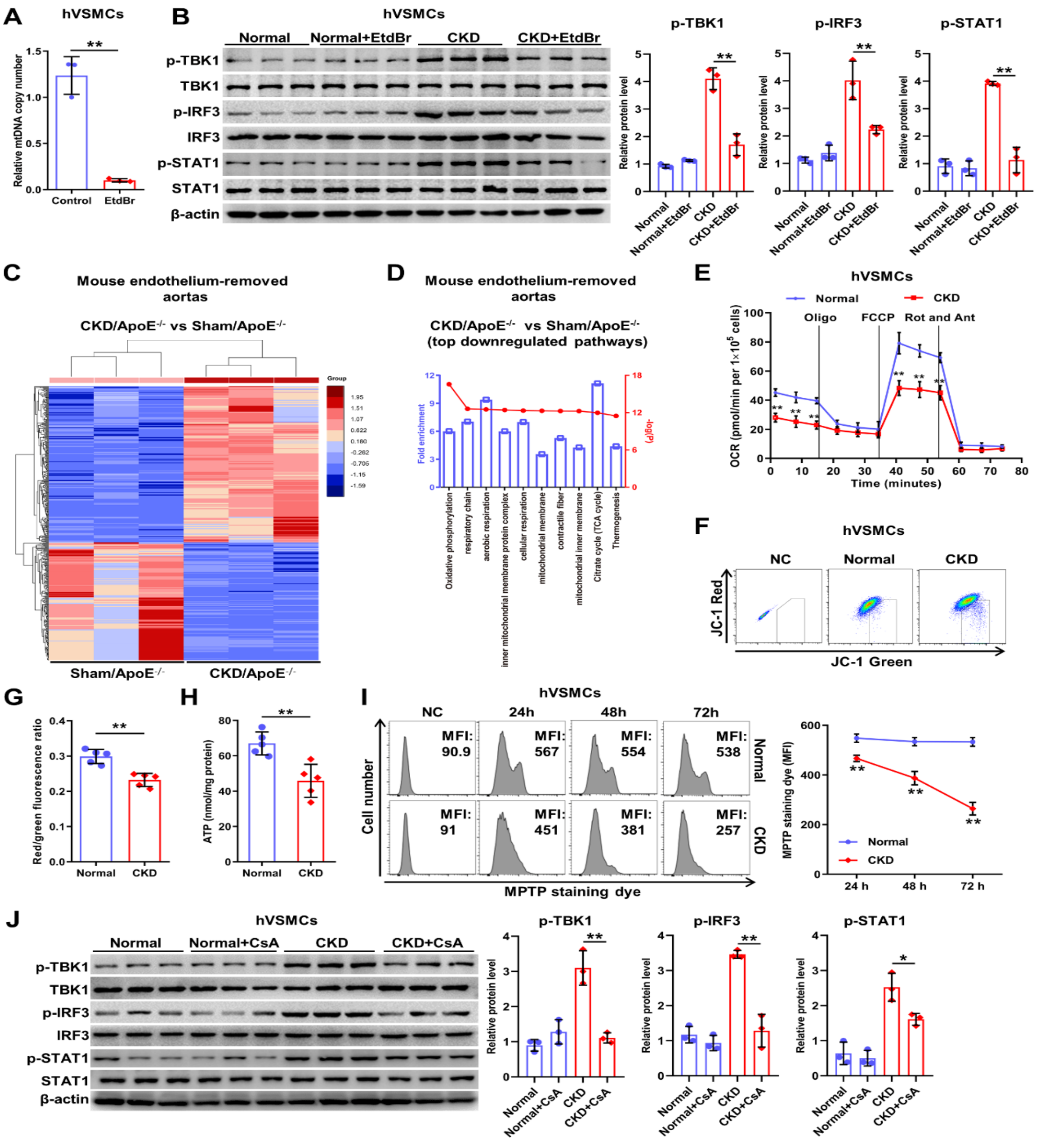

Figure S11. Endogenous mtDNA is responsible for CKD-induced cGAS-STING activation and subsequent IFN-I response in VSMCs. A) Quantitative PCR (qPCR) analysis of mtDNA contents in hVSMCs pretreated with or without EtdBr (*n* = 3). B) WB analysis of p-TBK1, p-IRF3 and p-STAT1 expression levels in hVSMCs incubated with normal or CKD serum after EtdBr pretreatment (*n* = 3). C) The heat map shows the differently expressed genes in endothelium-removed aortas between the two groups as indicated based on the proteomics data. D) KEGG and GO enrichment analysis of top downregulated pathways in endothelium-removed aortas of CKD/ApoE$^{-/-}$ mice based on the proteomics data. E) Seahorse analysis of oxygen consumption rate (OCR) in hVSMCs after incubation with normal or CKD serum (*n* = 3). F,G) Representative FC analysis and quantification of mitochondrial membrane potential in hVSMCs incubated with normal or CKD serum (*n* = 5). H) Relative ATP levels in hVSMCs incubated with normal or CKD serum (*n* = 5). I) The MPTP permeability in hVSMCs incubated with normal or CKD serum (*n* = 5). J) WB analysis of p-TBK1, p-IRF3 and p-STAT1 expression levels in hVSMCs incubated with normal or CKD serum after CsA pretreatment (*n* = 3). Data represent mean ± SD. $^{**}P < 0.01$, two-tailed Student's *t*-test was applied to E,G,H,I; one-way ANOVA was applied to B,J.

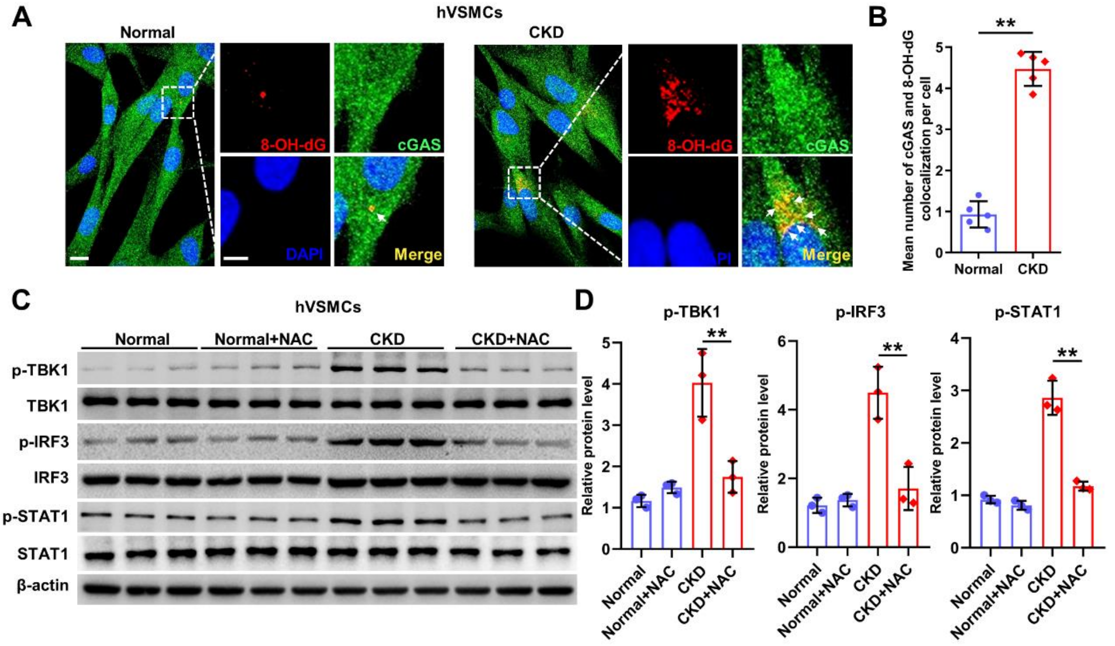

Figure S12. Oxidative stress plays a key role in CKD-induced mitochondrial damage and IFN-I response in hVSMCs. A,B) Representative images of cGAS and 8-OH-dG colocalization in hVSMCs incubated with normal or CKD serum. The box indicates the region magnified in the right panels. The arrow indicates cGAS and 8-OH-dG colocalization. Scale bar (lower magnification), 10 μm. Scale bar (higher magnification), 5 μm. C,D) The mean number of cGAS and 8-OH-dG colocalization per hVSMC after incubation with normal or CKD serum (*n* = 5 independent experiments). C) WB analysis of p-TBK1, p-IRF3 and p-STAT1 expression levels in hVSMCs incubated with normal or CKD serum after NAC pretreatment (*n* = 3). Data represent mean ± SD. ***P* < 0.01, two-tailed Student's *t*-test was applied to B; one-way ANOVA was applied to D.

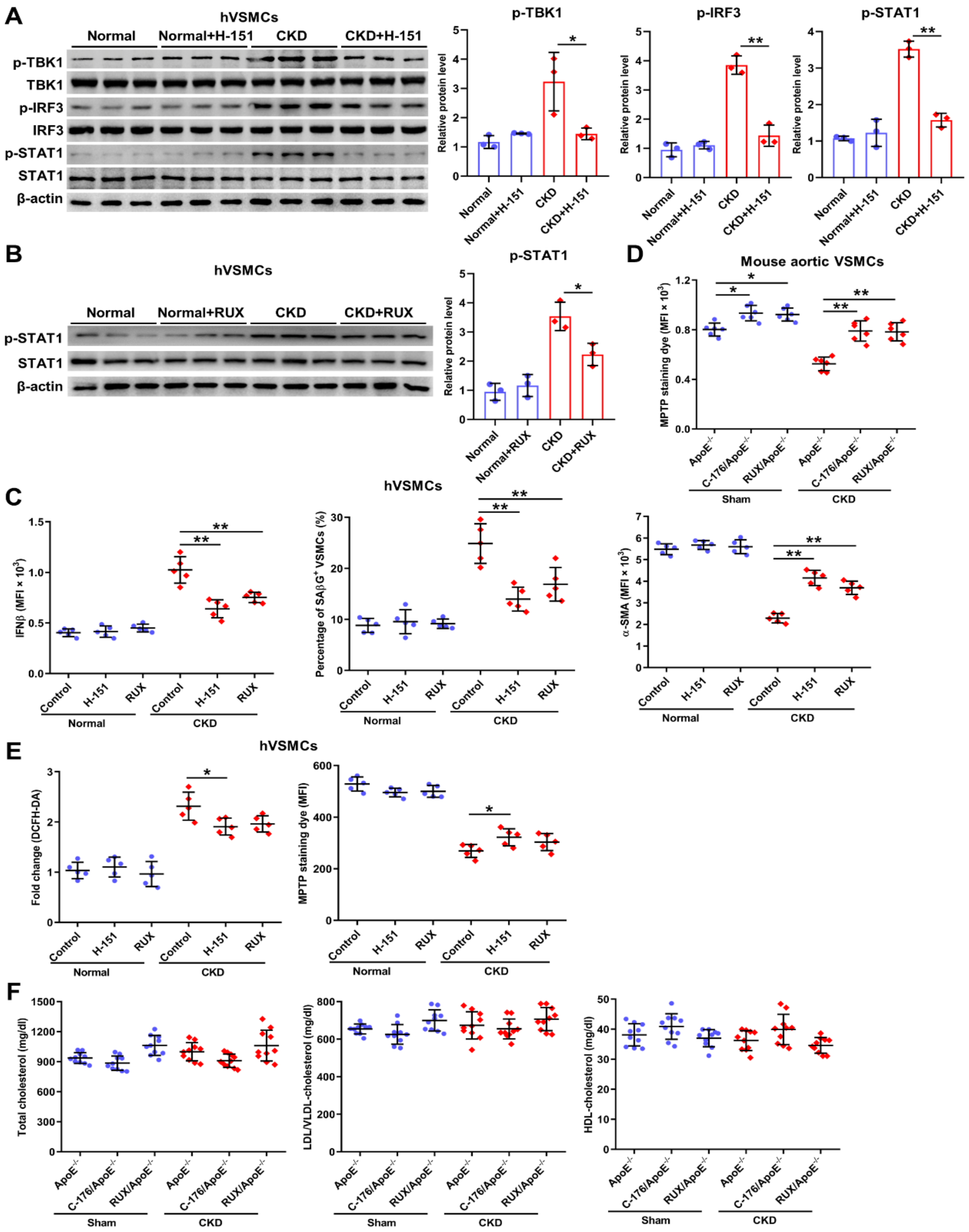

Figure S13. Pharmacological inhibition of IFN-I response mitigates oxidative stress and mitochondrial damage in VSMCs without affecting serum cholesterol levels. A,B) WB analysis of p-TBK1, p-IRF3 and p-STAT1 expression levels in hVSMCs incubated with normal or CKD serum after H-151 or RUX pretreatment (*n* = 3). C) The percentage of SAβG^{+} hVSMCs, as well as the MFIs of IFNβ and α-SMA in hVSMCs incubated with normal or CKD serum after H-151 or RUX pretreatment (*n* =5). D) The MPTP permeability in aortic VSMCs of Sham/ApoE$^{-/-}$ and CKD/ApoE$^{-/-}$ mice after C-176 or RUX treatment (*n* = 6). E) The fold change of DCFH-DA and MPTP permeability in hVSMCs incubated with normal or CKD serum after H-151 or RUX pretreatment (*n* =5). F) Serum levels of total cholesterol, LDL/VLDL-cholesterol and HDL-cholesterol in Sham/ApoE$^{-/-}$ and CKD/ApoE$^{-/-}$ mice after C-176 or RUX treatment (*n* = 10). Data represent mean ± SD. *P <0.05, **P <0.01, one-way ANOVA.

Table S1

	WD For 12 weeks		
**Parameter**	**Sham/ApoE$^{-/-}$**	**CKD/ApoE$^{-/-}$**	***P* Value (CKD/ApoE$^{-/-}$ vs Sham/ApoE$^{-/-}$)**
Number	10	10	——
Age (weeks)	22	22	——
Final weight (g)	31.05 ± 2.58	34.61 ± 3.33	NS
Creatinine (mg/dl)	0.63 ± 0.07	0.76 ± 0.09	$P < 0.01$
BUN (mg/dl)	41.11 ± 3.68	77.08 ± 5.86	$P < 0.01$

Data represent mean ± SD. One-way ANOVA. NS, not significant.

Table S2

Sham/ApoE$^{-/-}$			CKD/ApoE$^{-/-}$		
	Myocardial infarction			Myocardial infarction	
Days after WD	Anatomy	HE	Days after WD	Anatomy	HE
111			81		
147			107		
178			119		
192			133	×	✓
246	×	✓	150	×	✓
246			150		
263			169	✓	✓
277			189	×	✓
			189		
			202	✓	✓

The time and detection for myocardial infarction. "✓" represents "detected"; "×" represents "no obvious lesion observed".

Table S3

Antibody	Origin	Catalog number	Assay
p53 antibody	Cell Signaling Technology	Cat# 2524	WB, IF, FC
p16 antibody	Abcam	Cat# ab189034	WB, IF, FC
γ-H2AX	Cell Signaling Technology	Cat# 7631	IF
α-SMA antibody	Abcam	Cat# ab5694	WB, IF, IHC
CD31 antibody	Abcam	Cat# ab24590	IF
MYH11 antibody	Abcam	Cat# ab53219	WB
CNN1 antibody	Abcam	Cat# ab46794	WB
TAGLN antibody	Abcam	Cat# ab14106	WB
IFNβ antibody	Abcam	Cat# ab85803	WB, IF, FC
IFNAR1 antibody	Novus Biologicals	Cat# NBP2-67339	WB
cGAS antibody	Santa Cruz Biotechnology	Cat# sc-515777	WB, IF
STING antibody	Cell Signaling Technology	Cat# 13647	WB
p-TBK1 (Ser172) antibody	Cell Signaling Technology	Cat# 5483	WB, IF
TBK1 antibody	Abcam	Cat# ab40676	WB
p-IRF3 (Ser396) antibody	Cell Signaling Technology	Cat# 29047	WB, IF
IRF3 antibody	Cell Signaling Technology	Cat# 4302	WB

p-STAT1 (Tyr701) antibody	Cell Signaling Technology	Cat# 9167	WB, IF
STAT1 Antibody	Cell Signaling Technology	Cat# 14994	WB
IRF7 antibody	Novus Biologicals	Cat# NBP2-67634	WB
MX1 antibody	Abcam	Cat# ab222856	WB, FC
β-actin antibody	Abcam	Cat# ab8226	WB
cGAS antibody	Abcam	Cat# ab224144	IF
α-SMA antibody	Novus Biologicals	Cat# NB300-978	IF
HRP labeled goat anti-Rabbit IgG (H+L)	Abcam	Cat# 205718	WB, IHC
HRP labeled goat anti-Mouse IgG (H+L)	Abcam	Cat# 205719	WB
Ki-67 Monoclonal Antibody (SolA15), eFluor 660	eBioscience	Cat# 50-5698-80	FC
CD31 (PECAM-1) Monoclonal Antibody (390), eFluor 450	eBioscience	Cat# 48-0311-82	FC
CD45 Monoclonal Antibody (30-F11), eFluor 450	eBioscience	Cat# 48-0451-82	FC
TER-119 Monoclonal antibody (TER-119), eFluor 450	eBioscience	Cat# 48-5921-80	FC
CD31 (PECAM-1) Monoclonal Antibody (390), PE	eBioscience	Cat# 12-0311-82	FC
CD45 Monoclonal Antibody (30-F11), FITC	eBioscience	Cat# 11-0451-82	FC

TER-119 Monoclonal Antibody (TER-119), FITC	eBioscience	Cat# 11-5921-82	FC
CD31 (PECAM-1) Monoclonal Antibody (WM-59 (WM59)), FITC	eBioscience	Cat# 11-0319-42	FC
CD45 Monoclonal Antibody (HI30), FITC	eBioscience	Cat# 11-0459-42	FC
CD235a (Glycophorin A) Monoclonal Antibody (HIR2 (GA-R2)), FITC	eBioscience	Cat# 11-9987-82	FC
CD45 Monoclonal Antibody (HI30), PE	eBioscience	Cat# 12-0459-42	FC
CD31 (PECAM-1) Monoclonal Antibody (WM-59 (WM59)), eFluor 450	eBioscience	Cat# 48-0319-42	FC
CD45 Monoclonal Antibody (HI30), eFluor 450	eBioscience	Cat# 48-0459-42	FC
CD235a (Glycophorin A) Monoclonal Antibody (HIR2 (GA-R2)), eFluor 450	eBioscience	Cat# 48-9987-42	FC
CD11b Monoclonal Antibody (M1/70), APC	eBioscience	Cat# 17-0112-82	FC
CD68 Monoclonal Antibody (FA-11), PE	eBioscience	Cat# 12-0681-82	FC
α-SMA Monoclonal antibody (1A4), eFluor 660	eBioscience	Cat# 50-9760-82	FC
Anti-TAGLN/Transgelin antibody	Abcam	Cat# ab10135	FC
Anti-smooth muscle Myosin heavy chain 11 antibody [EPR5336**b**] (Alexa Fluor® 647)	Abcam	Cat# ab196982	FC
p-STAT1 (Ser727) antibody (Alexa Fluor 647 Conjugate)	Biolegend	Cat# 686412	FC

p-TBK1 (Ser172) antibody (Alexa Fluor 647 Conjugate)	Cell Signaling Technology	Cat# 14590	FC
p-IRF3 (Ser396) antibody (Alexa Fluor 647 Conjugate)	Cell Signaling Technology	Cat# 10327	FC
Goat anti-Rabbit IgG (H+L) Cross-Adsorbed Secondary antibody, Alexa Fluor 647	Thermo Fisher Scientific	Cat# A-21244	FC
Goat anti-Mouse IgG (H+L) Cross-Adsorbed Secondary Antibody, Alexa Fluor 647	Thermo Fisher Scientific	Cat# A-21235	FC
8-OH-dG antibody	Abcam	Cat# ab48508	IF
DNA antibody	Millipore	Cat# CBL186	IF
Goat anti-Mouse IgG (H+L) Cross-Adsorbed Secondary antibody, Alexa Fluor 488	Thermo Fisher Scientific	Cat# A-11001	IF
Goat anti-Mouse IgG (H+L) Cross-Adsorbed Secondary antibody, Alexa Fluor 546	Thermo Fisher Scientific	Cat# A-11003	IF
Goat anti-Rabbit IgG (H+L) Cross-Adsorbed Secondary antibody, Alexa Fluor 488	Thermo Fisher Scientific	Cat# A-11008	IF
Goat anti-Rabbit IgG (H+L) Cross-Adsorbed Secondary antibody, Alexa Fluor 546	Thermo Fisher Scientific	Cat# A-11010	IF
Donkey anti-Goat IgG (H+L) Cross-Adsorbed Secondary antibody, Alexa Fluor 647	Abcam	Cat# ab150131	IF
Monoclonal ANTI-Flag® M2 antibody	Sigma-Aldrich	Cat# F1804	WB, ChIP

Antibodies used in WB, IF, FC, IHC and ChIP analysis.

Table S4

Gene name	Regulated Type	CKD/ApoE$^{-/-}$/Sham/ApoE$^{-/-}$ P value	Sham/ApoE-/-1	Sham/ApoE-/-2	Sham/ApoE-/-3	CKD/ApoE$^{-/-}$1	CKD/ApoE$^{-/-}$2	CKD/ApoE$^{-/-}$3
Ttn	Down	0.0236	1.653	1.132	2.943	0.302	0.293	0.302
Myh4	Down	0.00872	1.905	1.281	2.613	0.208	0.208	0.202
Myh1	Down	0.035	2.61	0.865	2.083	0.285	0.283	0.301
Myh8	Down	0.00496	2.69	1.224	1.112	0.2	0.333	0.268
Acta1	Down	0.00748	2.123	1.369	1.799	0.396	0.403	0.377
Fbn1	Up	0.00652	0.771	0.721	0.662	1.375	1.024	1.354
Atp2a1	Down	0.0207	1.579	1.075	3.132	0.215	0.199	0.198
Myh3	Down	0.000561	1.872	1.582	2.658	0.272	0.188	0.15
Alb	Up	0.00892	0.757	0.776	0.659	1.039	1.192	1.437
Myl1	Down	0.0196	1.744	1.117	2.948	0.251	0.251	0.242
Mybpc2	Down	0.023	1.557	1.141	3.113	0.249	0.275	0.241
Aco2	Down	0.0293	1.425	1.053	1.329	0.889	0.881	0.653
Ckm	Down	0.0342	1.446	1.023	3.45	0.259	0.267	0.237
Fn1	Up	0.00526	0.639	0.813	0.604	1.454	1.252	1.147
Tf	Up	0.000461	0.456	0.5	0.44	1.199	1.469	1.696
Mylpf	Down	0.0225	1.558	1.108	3.065	0.251	0.242	0.242
Pygm	Down	0.00852	1.362	1.04	3.359	0.325	0.306	0.257
Flnc	Down	0.0136	1.109	1.217	1.784	0.761	0.704	0.691
Actn3	Down	0.0064	2.518	1.47	1.803	0.272	0.263	0.262
Myom1	Down	0.00314	1.646	1.093	3.23	0.262	0.214	0.226
Acly	Down	0.0389	1.721	1.152	1.094	0.654	0.75	0.916
Tagln2	Up	0.033	0.336	0.583	0.505	1.625	2.03	0.766
Ryr1	Down	0.0327	1.481	1.114	2.667	0.406	0.405	0.416
C3	Up	0.000358	0.605	0.634	0.573	1.208	1.336	1.513
Mdh2	Down	0.0269	1.388	0.998	1.411	0.886	0.828	0.729
Lmna	Down	0.0422	1.054	1.515	1.273	0.672	0.678	1.004
Msn	Up	0.0181	0.736	0.849	0.709	1.246	1.445	0.998
Eno3	Down	0.00532	1.506	1.034	3.552	0.258	0.227	0.21
Obscn	Down	0.0383	1.488	1.057	2.662	0.424	0.425	0.42
Atp1a2	Down	0.0178	1.237	1.053	1.712	0.779	0.739	0.716
Cp	Up	0.000517	0.547	0.585	0.539	1.214	1.328	1.618
Gpd2	Down	0.00832	1.443	1.101	1.392	0.841	0.808	0.677
Actn2	Down	0.00654	2.497	1.082	1.727	0.519	0.406	0.429
Fga	Up	0.0307	0.921	0.751	0.516	1.312	1.166	1.28
Cfh	Up	0.000201	0.492	0.621	0.544	1.343	1.426	1.346
Srl	Down	0.00814	1.492	1.004	3.167	0.355	0.31	0.281
Cs	Down	0.00878	1.495	1.106	1.353	0.864	0.811	0.717

Mrc1	Up	0.000222	0.633	0.609	0.582	1.281	1.271	1.52
Calr	Up	0.00188	0.788	0.828	0.732	1.247	1.292	1.102
Pgm1	Down	0.0493	1.273	0.923	2.322	0.592	0.691	0.67
Pvalb	Down	0.0341	1.531	0.946	3.355	0.26	0.236	0.218
Pdha1	Down	0.00736	1.473	1.093	1.469	0.844	0.748	0.745
Pls3	Up	0.0319	0.539	0.898	0.666	1.259	1.483	1.021
Sucla2	Down	0.0245	1.5	1.093	1.413	0.852	0.877	0.601
Hpx	Up	0.000856	0.454	0.469	0.405	1.199	1.361	1.821
Dlat	Down	0.0041	1.408	1.116	1.447	0.826	0.75	0.721
Pdhb	Down	0.00638	1.442	1.082	1.282	0.834	0.8	0.79
Idh3a	Down	0.0137	1.413	1.104	1.351	0.876	0.9	0.717
Pfkm	Down	0.0204	1.289	1.034	2.672	0.538	0.553	0.425
Anpep	Up	0.00116	0.53	0.71	0.604	1.247	1.301	1.429
Anxa1	Up	0.00152	0.604	0.783	0.649	1.316	1.357	1.212
Anxa5	Up	0.000865	0.675	0.769	0.668	1.216	1.37	1.177
Apob	Up	0.0000159	0.424	0.407	0.384	1.427	1.445	1.617
Gdi1	Up	0.0221	0.752	0.904	0.682	1.101	1.415	1.088
Ctsb	Up	0.00514	0.851	0.61	0.727	1.298	1.263	1.198
Slc25a4	Down	0.0115	1.345	1.126	2.375	0.602	0.53	0.543
Fh	Down	0.0273	1.445	1.015	1.314	0.873	0.867	0.712
Vdac1	Down	0.0143	1.415	1.067	1.774	0.782	0.744	0.646
Pzp	Up	0.00572	0.659	0.698	0.485	1.205	1.203	1.57
Ldb3	Down	0.0338	1.223	1.187	1.95	0.6	0.61	0.6
Sod3	Up	0.046	0.42	0.763	0.561	1.63	1.675	0.812
Sptb	Down	0.0045	1.362	1.291	1.239	0.913	0.701	0.707
Gstm2	Up	0.024	0.611	0.855	0.738	1.213	1.497	1.019
Hp	Up	0.000978	0.399	0.4	0.314	1.257	1.331	1.959
Aebp1	Up	0.0251	0.593	0.875	0.714	1.314	1.351	0.994
Tnnc2	Down	0.000401	2.458	1.346	2.45	0.227	0.178	0.188
Prelp	Up	0.00284	0.618	0.795	0.74	1.144	1.25	1.285
Ckmt2	Down	0.0199	1.623	0.918	2.836	0.551	0.377	0.276
F13a1	Up	0.0062	0.717	0.682	0.482	1.237	1.205	1.492
Ace	Up	0.0086	0.462	0.673	0.611	1.535	1.6	1.032
Efemp1	Up	0.0251	0.582	0.923	0.724	1.275	1.32	1.078
Apoa4	Up	0.000883	0.527	0.592	0.477	1.278	1.272	1.666
Serpina1b	Up	0.000445	0.754	0.726	0.657	1.105	1.169	1.169
Ncam1	Up	0.0146	0.718	0.886	0.768	1.264	1.302	1.017
Serpina3k	Up	0.00994	0.716	0.873	0.626	1.107	1.172	1.249
Tpm4	Up	0.00838	0.754	0.74	0.522	1.435	1.157	1.25
Pgam2	Down	0.0392	1.432	0.956	3.405	0.278	0.269	0.247

Ctsd	Up	0.0022	0.78	0.67	0.73	1.358	1.314	1.103
Itgav	Up	0.0144	0.6	0.86	0.678	1.285	1.446	1.06
Nnt	Down	0.00406	1.376	1.423	2.404	0.565	0.375	0.355
Suclg1	Down	0.0328	1.533	1.072	1.275	0.867	0.881	0.618
Cdh13	Up	0.0321	0.626	0.963	0.795	1.219	1.254	1.106
Tnc	Up	0.00458	0.574	0.677	0.566	1.557	1.44	1.037
Ighm	Up	0.00144	0.597	0.487	0.514	1.21	1.259	1.686
Gc	Up	0.000643	0.6	0.603	0.517	1.187	1.383	1.533
Cycs	Down	0.0392	1.56	0.967	1.544	0.845	0.831	0.639
Serpina 1e	Up	0.00186	0.424	0.5	0.323	1.213	1.383	1.803
Cd163	Up	0.000856	0.635	0.772	0.626	1.245	1.346	1.264
Hadh	Down	0.0395	1.658	1.074	1.168	0.851	0.893	0.787
Fgg	Up	0.0191	0.86	0.72	0.484	1.356	1.199	1.348
Fbln2	Up	0.0118	0.682	0.857	0.799	1.344	1.286	1.045
Anxa4	Up	0.000955	0.641	0.755	0.69	1.276	1.376	1.157
Kng1	Up	0.0013	0.509	0.558	0.489	1.16	1.353	1.733
C4b	Up	0.00154	0.831	0.737	0.687	1.174	1.219	1.317
Idh3g	Down	0.0101	1.337	1.082	1.394	0.851	0.867	0.727
Anxa7	Up	0.0125	0.687	0.888	0.726	1.209	1.38	1.068
Dlst	Down	0.0159	1.418	1.096	1.263	0.911	0.832	0.694
Pbxip1	Down	0.0284	0.963	1.593	1.827	0.752	0.74	0.547
Cpt1b	Down	0.0482	1.549	1.183	1.67	0.863	0.831	0.381
Itih4	Up	0.0000625	0.505	0.586	0.496	1.368	1.465	1.394
Igkc	Up	0.0018	0.704	0.731	0.668	1.12	1.211	1.439
Tnni2	Down	0.000203	2.466	1.441	2.237	0.204	0.191	0.232
Rbp1	Up	0.0328	0.519	0.819	0.66	1.251	1.712	0.977
Ppa1	Up	0.033	0.832	0.684	0.709	0.985	1.111	1.561
Plg	Up	0.00262	0.69	0.814	0.675	1.174	1.195	1.406
Itih1	Up	0.000761	0.586	0.636	0.726	1.461	1.342	1.235
Casq1	Down	0.00182	1.8	1.145	2.833	0.279	0.251	0.242
Ahsg	Up	0.00146	0.617	0.581	0.491	1.161	1.337	1.584
Cd36	Down	0.0368	1.55	1.15	1.111	0.727	0.661	0.97
Atp5h	Down	0.0468	1.254	0.968	1.571	0.819	0.718	0.884
Cox6b1	Down	0.0354	1.451	0.992	1.392	0.898	0.855	0.725
Clu	Up	0.0000162	0.512	0.576	0.529	1.394	1.367	1.406
Vcan	Up	0.0284	0.538	0.869	0.778	1.428	1.257	1.079
Vat1	Up	0.0026	0.765	0.851	0.679	1.214	1.259	1.161
Pdhx	Down	0.00514	1.401	1.097	1.295	0.859	0.823	0.785
Myoz1	Down	0.00538	2.777	1.42	1.193	0.409	0.348	0.424
Itga5	Up	0.0272	0.64	0.916	0.7	1.364	1.399	1.006
Rcn2	Up	0.042	0.654	0.892	0.752	1.371	1.34	0.932
Ddah2	Up	0.00968	0.647	0.903	0.719	1.17	1.293	1.177

Naalad 2	Up	0.000244	0.683	0.77	0.753	1.179	1.254	1.217
Inmt	Up	0.0107	0.597	0.665	0.526	1.05	1.166	1.766
Vdac2	Down	0.0339	1.173	1.147	1.491	0.824	0.799	0.794
Gstm7	Up	0.0349	0.411	0.732	0.611	1.27	2.002	0.957
F2	Up	0.0024	0.626	0.768	0.533	1.271	1.357	1.351
Cox4i1	Down	0.00856	1.563	1.097	1.671	0.787	0.68	0.606
Grn	Up	0.00142	0.686	0.556	0.662	1.336	1.168	1.476
Mybph	Down	0.0302	1.408	1.197	2.897	0.371	0.348	0.379
Ampd1	Down	0.0425	1.324	1.04	3.329	0.329	0.302	0.31
Uqcrfs 1	Down	0.022	1.412	1.041	1.428	0.932	0.8	0.778
Tnnt3	Down	0.000724	2.572	1.218	1.954	0.223	0.178	0.149
Gys1	Down	0.0183	1.428	1.003	1.86	0.631	0.656	0.749
Fbln1	Up	0.0071	0.578	0.825	0.701	1.205	1.198	1.45
Vdac3	Down	0.0054	1.322	1.132	1.658	0.767	0.681	0.699
Ndufa4	Down	0.0144	1.487	1.093	1.382	0.873	0.802	0.646
Aspn	Up	0.000521	0.541	0.669	0.597	1.436	1.423	1.225
Atp5f1	Down	0.0352	1.27	0.993	1.55	0.817	0.758	0.881
Ndufs3	Down	0.00306	1.261	1.114	1.356	0.872	0.812	0.802
Rnase4	Up	0.00152	0.524	0.63	0.543	1.404	1.595	1.134
Tubb1	Down	0.0356	1.361	1.629	0.939	0.811	0.742	0.739
Cfb	Up	0.00118	0.6	0.74	0.571	1.37	1.37	1.216
Capg	Up	0.00114	0.783	0.65	0.774	1.302	1.222	1.219
Vcam1	Up	0.0072	0.53	0.588	0.475	1.857	1.445	0.997
Serpinf 1	Up	0.00748	0.509	0.831	0.565	1.413	1.398	1.238
Apoh	Up	0.0146	0.779	0.903	0.636	1.291	1.12	1.2
Trim72	Down	0.0379	1.284	0.96	2.456	0.702	0.578	0.514
Crk	Up	0.0284	0.691	0.925	0.698	1.202	1.409	1.017
Uqcrb	Down	0.00994	1.506	1.081	1.387	0.799	0.823	0.673
Nipsna p2	Down	0.0384	1.285	0.982	2.142	0.713	0.632	0.72
Phka1	Down	0.0125	1.284	1.136	2.663	0.439	0.472	0.545
Hexb	Up	0.000696	0.757	0.713	0.759	1.313	1.262	1.116
Clec3b	Up	0.03	0.543	0.929	0.623	1.103	1.195	1.512
Smoc1	Up	0.0204	0.626	0.784	0.797	1.328	1.454	0.984
Slc25a 1	Down	0.0138	1.442	1.215	1.174	0.701	0.736	0.951
Gda	Up	0.00756	0.797	0.621	0.64	1.253	1.07	1.483
Icam1	Up	0.00514	0.765	0.713	0.647	1.357	1.327	1.034
Serpina 3n	Up	0.000824	0.497	0.531	0.603	1.465	1.598	1.185

Mpst	Down	0.00442	1.423	1.158	1.297	0.778	0.683	0.858
Uchl1	Up	0.00346	0.528	0.691	0.586	1.331	1.642	1.137
Htra1	Up	0.0172	0.448	0.829	0.53	1.579	1.341	1.107
Entpd2	Up	0.0109	0.588	0.862	0.663	1.121	1.431	1.259
Ctsh	Up	0.000536	0.719	0.713	0.662	1.406	1.347	1.169
Ecm1	Up	0.00356	0.712	0.802	0.74	1.458	1.299	1.108
C8a	Up	0.00194	0.749	0.755	0.596	1.396	1.262	1.237
Coq9	Down	0.0175	1.249	1.014	1.453	0.851	0.775	0.786
Emilin 2	Up	0.0035	0.647	0.7	0.7	1.349	1.047	1.437
Slc25a 11	Down	0.0172	1.34	1.021	1.566	0.824	0.767	0.751
Slc25a 10	Down	0.0048	1.462	1.169	1.304	0.683	0.718	0.868
Cd200	Up	0.014	0.577	0.579	0.653	1.55	1.763	0.938
Itgam	Up	0.0081	0.845	0.591	0.699	1.327	1.12	1.346
Nucb2	Up	0.0033	0.683	0.854	0.805	1.271	1.26	1.159
Smyd1	Down	0.0414	1.344	1.099	2.876	0.389	0.411	0.402
Coq8a	Down	0.0326	1.29	1.054	1.659	0.845	0.875	0.655
Cd34	Up	0.0215	0.671	0.89	0.803	1.194	1.356	1.034
Cd109	Up	0.0277	0.468	0.765	0.617	1.539	1.586	0.916
Stab1	Up	0.0000191	0.749	0.725	0.729	1.208	1.233	1.291
Scpep1	Up	0.00592	0.765	0.823	0.677	1.3	1.343	1.066
Csad	Up	0.0134	0.859	0.796	0.633	1.133	1.394	1.153
Nebl	Up	0.0167	0.704	0.743	0.796	1.403	1.521	0.989
Ctsz	Up	0.00772	0.822	0.558	0.732	1.321	1.286	1.177
Klhl41	Down	0.03	1.59	1.183	2.694	0.442	0.452	0.427
Scarb2	Up	0.0000842	0.527	0.573	0.603	1.382	1.438	1.265
Igh-3	Up	0.000942	0.759	0.752	0.73	1.156	1.179	1.381
Cpa3	Up	0.0203	0.851	0.751	0.609	1.086	1.15	1.51
Lgals3	Up	0.0011	0.822	0.672	0.711	1.254	1.21	1.31
Mfge8	Up	0.0161	0.748	0.825	0.575	1.535	1.134	1.162
Dpep1	Up	0.00284	0.613	0.818	0.667	1.189	1.338	1.284
Ctsc	Up	0.00478	0.824	0.594	0.727	1.257	1.182	1.256
Dab2	Up	0.0067	0.78	0.693	0.759	1.172	1.078	1.459
Ambp	Up	0.000257	0.433	0.465	0.434	1.255	1.421	1.734
Col28a 1	Up	0.00692	0.737	0.596	0.558	1.066	1.264	1.575
Itih3	Up	0.001	0.528	0.597	0.518	1.173	1.256	1.596
Uqcr10	Down	0.0248	1.435	1.024	1.487	0.848	0.888	0.766
Mylk2	Down	0.0206	1.315	0.991	2.894	0.507	0.48	0.392
Cisd1	Down	0.0119	1.266	1.069	1.506	0.847	0.811	0.775

Ppp1r3a	Down	0.00838	1.572	1.018	3.022	0.335	0.321	0.405
Ndufa2	Down	0.00954	1.425	1.069	1.38	0.862	0.787	0.725
Myot	Down	0.0192	1.903	1.124	2.66	0.31	0.294	0.291
Efemp2	Up	0.025	0.521	0.747	0.618	1.455	1.642	0.905
Cfd	Up	0.0023	0.711	0.725	0.74	1.061	1.185	1.358
C4bpa	Up	0.00116	0.447	0.404	0.392	1.67	1.638	1.073
Fth1	Up	0.0105	0.675	0.553	0.51	1.022	1.225	1.756
Cfp	Up	0.00172	0.442	0.388	0.352	2.039	1.596	1.13
Th	Up	0.0289	0.675	0.69	0.86	1.017	1.174	1.614
Marcks	Up	0.00966	0.864	0.696	0.755	1.146	1.093	1.386
Ftl1	Up	0.032	0.738	0.436	0.533	0.955	1.185	1.982
Clec10a	Up	0.00878	0.754	0.611	0.529	1.281	1.09	1.631
Tpm2	Down	0.00024	2.772	1.45	1.831	0.187	0.163	0.157
Rab6b	Up	0.000936	0.636	0.702	0.772	1.375	1.261	1.202
Itih2	Up	0.00396	0.669	0.812	0.569	1.254	1.229	1.34
Gstt1	Up	0.00328	0.683	0.829	0.674	1.118	1.288	1.33
Top2b	Down	0.0035	1.551	1.323	1.187	0.692	0.623	0.798
Vwa1	Up	0.00178	0.746	0.718	0.679	1.355	1.292	1.08
Basp1	Up	0.0246	0.984	0.681	0.633	1.197	1.183	1.344
Tecr	Down	0.011	1.495	1.095	1.485	0.651	0.614	0.858
Hrg	Up	0.000702	0.484	0.579	0.537	1.273	1.294	1.674
Dpp7	Up	0.000178	0.76	0.824	0.787	1.181	1.233	1.154
Ltbp2	Up	0.0185	0.611	0.789	0.728	1.573	1.251	1.004
Itgb5	Up	0.00684	0.481	0.765	0.556	1.378	1.548	1.153
Ifi204	Up	0.0242	0.856	0.546	0.647	1.196	1.066	1.625
Camk2a	Down	0.00416	1.568	1.141	3.054	0.289	0.293	0.346
Ctsa	Up	0.0239	0.986	0.691	0.739	1.284	1.257	1.104
Serpind1	Up	0.0105	0.474	0.676	0.603	1.058	1.242	1.752
Gaa	Up	0.000878	0.78	0.809	0.78	1.252	1.331	1.127
Serping1	Up	0.000856	0.645	0.729	0.667	1.147	1.274	1.41
Rbbp7	Down	0.0162	1.288	1.04	1.534	0.776	0.823	0.82
Acan	Up	0.0363	0.442	0.597	0.616	1.828	1.696	0.783
Speg	Down	0.0482	1.309	1.099	1.951	0.687	0.634	0.673
Steap3	Up	0.00756	0.729	0.866	0.768	1.262	1.063	1.356
Cbr3	Up	0.000281	0.655	0.753	0.752	1.308	1.255	1.262
Arl8a	Up	0.000935	0.688	0.816	0.672	1.221	1.299	1.286
Npnt	Up	0.0262	0.584	0.949	0.696	1.313	1.303	1.099
Ndufb9	Down	0.00908	1.384	1.051	1.303	0.841	0.84	0.806

Cd5l	Up	0.0000176	0.354	0.369	0.305	1.603	1.628	1.522
Mfap5	Up	0.0124	0.791	0.661	0.541	1.495	1.037	1.399
Reck	Up	0.0435	0.574	0.927	0.795	1.241	1.384	1.037
Tcof1	Down	0.01	1.356	1.037	1.337	0.826	0.793	0.841
Pdk4	Down	0.0152	1.767	1.217	1.287	0.827	0.763	0.552
Saa2	Up	0.0332	0.266	0.279	0.292	2.226	0.799	1.703
C9	Up	0.0000952	0.682	0.717	0.653	1.367	1.242	1.266
C5	Up	0.000242	0.52	0.62	0.489	1.422	1.352	1.315
Ctsl	Up	0.000437	0.693	0.735	0.609	1.356	1.281	1.249
Apcs	Up	0.00058	0.422	0.305	0.429	1.283	1.687	1.598
Cox7c	Down	0.00974	1.428	1.085	1.832	0.696	0.566	0.704
Akr1b8	Up	0.000363	0.776	0.839	0.75	1.183	1.249	1.18
Atp6v0d1	Up	0.00108	0.802	0.779	0.697	1.253	1.151	1.319
Cacna1s	Down	0.0316	1.572	1.124	2.894	0.362	0.398	0.358
Ugt1a7c	Up	0.00496	0.637	0.53	0.625	1.287	1.08	1.693
Asrgl1	Down	0.0032	1.25	1.153	1.476	0.784	0.774	0.831
Cacnb1	Down	0.0371	1.475	1.106	2.777	0.436	0.411	0.407
Ndufa6	Down	0.00242	1.26	1.137	1.413	0.824	0.793	0.831
Mpc2	Down	0.000776	1.429	1.238	1.583	0.711	0.67	0.729
Ggt5	Up	0.00622	0.788	0.69	0.821	1.137	1.149	1.425
Alyref	Down	0.00144	1.373	1.147	1.378	0.817	0.776	0.789
Anxa8	Up	0.0118	0.755	0.881	0.664	1.078	1.222	1.367
Lgmn	Up	0.00702	0.895	0.705	0.804	1.317	1.222	1.122
App	Up	0.045	0.457	0.726	0.549	1.587	1.809	0.787
Gba	Up	0.0000769	0.815	0.759	0.758	1.239	1.19	1.223
Rcn1	Up	0.00348	0.729	0.882	0.739	1.247	1.213	1.124
Fcgrt	Up	0.000505	0.764	0.823	0.764	1.311	1.186	1.172
Islr	Up	0.0431	0.672	0.929	0.796	1.307	1.324	0.968
Atl2	Down	0.0351	1.207	1.025	1.574	0.832	0.884	0.78
C8b	Up	0.000582	0.619	0.708	0.561	1.424	1.259	1.324
Abca9	Up	0.000204	0.713	0.738	0.654	1.22	1.241	1.338
Uap1	Up	0.0013	0.815	0.843	0.721	1.238	1.157	1.248
Jph1	Down	0.0304	1.618	1.091	2.966	0.333	0.359	0.319
Tmod4	Down	0.00066	1.954	1.302	1.845	0.491	0.444	0.492
Apobec2	Down	0.0271	1.411	1.207	3.236	0.291	0.259	0.294
Trdn	Down	0.0168	1.509	1.003	2.375	0.574	0.52	0.602
Rtn2	Down	0.0207	1.301	1.011	2.831	0.532	0.488	0.423
Emp2	Up	0.0383	0.548	0.885	0.85	1.267	1.229	1.119
Abcb1b	Up	0.0306	0.607	0.607	0.801	1.178	1.779	0.985

Tmod1	Down	0.0000556	1.906	1.534	1.604	0.494	0.477	0.47
Golga3	Up	0.00382	0.725	0.849	0.674	1.121	1.255	1.311
Mp68	Down	0.0157	1.349	1.035	1.498	0.781	0.743	0.862
Chl1	Up	0.0374	0.866	0.82	0.678	1.11	1.011	1.462
Nfix	Down	0.0389	1.068	1.163	2.088	0.56	0.645	0.822
Tmem38a	Down	0.00472	1.54	1.083	3.416	0.279	0.25	0.22
Fhod1	Down	0.00312	1.358	1.173	1.643	0.763	0.695	0.706
Mtch2	Down	0.000279	1.298	1.165	1.276	0.834	0.808	0.808
Naprt	Up	0.00296	0.749	0.822	0.802	1.067	1.288	1.241
Dhrs7c	Down	0.00644	1.298	1.139	3.036	0.355	0.326	0.286
Rab11fip5	Down	0.00656	1.456	1.119	1.454	0.812	0.811	0.672
Pofut2	Up	0.0304	0.616	0.875	0.807	1.326	1.373	1.002
Dap	Up	0.0102	0.652	0.779	0.596	1.265	1.589	1.057
Ppif	Down	0.0464	1.486	0.938	1.433	0.877	0.81	0.754
Ndufa3	Down	0.025	1.196	1.03	1.521	0.859	0.821	0.781
Sdhc	Down	0.0139	1.302	1.084	1.617	0.759	0.809	0.836
Nop56	Down	0.00264	1.453	1.178	1.528	0.744	0.619	0.75
Fetub	Up	0.000337	0.559	0.494	0.535	1.234	1.398	1.614
Endog	Down	0.0158	1.485	1.022	1.746	0.737	0.753	0.642
Saa1	Up	0.0000976	0.435	0.485	0.455	1.281	1.574	1.555
Mmp2	Up	0.0193	0.36	0.844	0.393	1.566	1.341	1.311
Mypn	Down	0.022	1.402	0.992	2.441	0.602	0.471	0.598
Gas7	Up	0.0127	0.771	0.697	0.459	1.349	1.156	1.439
Surf4	Down	0.00622	1.41	1.133	1.81	0.711	0.62	0.697
Gns	Up	0.00226	0.791	0.686	0.702	1.326	1.326	1.094
Ganc	Down	0.0239	1.433	1.119	1.304	0.795	0.821	0.8
Ufl1	Down	0.00316	1.239	1.15	1.451	0.841	0.803	0.784
Gfm2	Down	0.0139	1.189	1.158	1.409	0.849	0.706	0.922
Ces2c	Up	0.0018	0.728	0.792	0.736	1.289	1.336	1.093
Cisd2	Down	0.00706	1.367	1.091	1.538	0.816	0.768	0.734
Ndufb3	Down	0.0168	1.413	1.032	1.345	0.857	0.728	0.846
Arpin	Up	0.0263	0.741	0.885	0.694	1.154	1.403	0.997
Cul2	Down	0.0129	1.271	1.103	1.396	0.94	0.813	0.745
Osbpl3	Down	0.0147	1.078	1.277	1.723	0.671	0.763	0.778
Pdk2	Down	0.0143	1.269	1.067	1.586	0.819	0.806	0.752
Matn2	Up	0.0044	0.737	0.742	0.688	1.253	1.058	1.447
Rbbp9	Up	0.0167	0.757	0.867	0.767	1.149	1.434	1.044
Sypl2	Down	0.006	1.403	1.035	3.721	0.255	0.189	0.199
Mt2	Up	0.0000815	0.273	0.281	0.292	1.32	1.893	1.638
Orm2	Up	0.00408	0.423	0.347	0.545	1.122	1.388	1.923
Fcgr2	Up	0.00536	0.539	0.631	0.512	1.285	1.069	1.769

Apoe	Down	0.000161	1.984	1.553	1.646	0.498	0.397	0.437
F9	Up	0.025	0.67	0.667	0.729	1.358	1.543	0.895
Cyr61	Up	0.0146	0.718	0.844	0.779	1.376	1.278	1.002
Ang	Up	0.000238	0.473	0.599	0.545	1.367	1.51	1.351
Mmp3	Up	0.0186	0.548	0.617	0.706	1.011	1.152	1.798
Vtn	Up	0.00406	0.622	0.61	0.52	1.344	1.075	1.683
Saa4	Up	0.0024	0.609	0.638	0.451	1.235	1.302	1.563
Apoc1	Up	0.0222	0.712	0.828	0.488	1.414	1.277	1.08
Mbl1	Up	0.0144	0.538	0.599	0.755	1.643	1.427	0.988
Ephb2	Down	0.0169	1.268	1.058	1.513	0.887	0.767	0.789
Pltp	Up	0.000182	0.643	0.646	0.709	1.224	1.285	1.399
Cox7a1	Down	0.043	1.739	1.087	1.963	0.748	0.701	0.275
Ncstn	Up	0.00562	0.81	0.867	0.658	1.254	1.222	1.189
Rap2b	Up	0.000402	0.768	0.795	0.732	1.152	1.206	1.301
Rps15	Down	0.0202	1.571	1.011	1.455	0.841	0.765	0.698
Mpc1	Down	0.0000437	1.267	1.205	1.233	0.774	0.793	0.739
Nsun2	Down	0.0435	1.406	1.115	1.232	0.627	0.929	0.923
Jsrp1	Down	0.00436	1.717	1.075	3.015	0.278	0.285	0.342
Plcl1	Down	0.00718	2.005	1.16	3.505	0.198	0.028	0.032
Plch1	Down	0.0189	1.439	1.132	1.387	0.911	0.828	0.62
Gas6	Up	0.0185	0.54	0.763	0.582	1.338	1.332	1.322
Azgp1	Up	0.0094	0.586	0.87	0.553	1.261	1.366	1.256
Pcp4l1	Up	0.0305	0.486	0.929	0.557	1.268	1.525	1.109
Ankib1	Down	0.0458	1.681	0.898	3	0.353	0.319	0.353
Cmya5	Down	0.0272	1.227	1.007	2.736	0.49	0.584	0.493
Enoph1	Up	0.000799	0.775	0.756	0.826	1.115	1.245	1.259
Pgghg	Up	0.00106	0.697	0.74	0.689	1.257	1.404	1.125
Osbpl2	Up	0.00166	0.695	0.748	0.615	1.145	1.288	1.408
Aida	Up	0.0237	0.71	0.988	0.684	1.235	1.192	1.154
C1sa	Up	0.000204	0.738	0.704	0.647	1.314	1.211	1.316
Slc25a42	Down	0.0155	1.477	1.107	1.235	0.879	0.879	0.726
Srgap2	Down	0.0201	1.102	1.145	1.493	0.765	0.824	0.902
Tspan31	Up	0.00516	0.639	0.785	0.625	1.419	1.392	1.076
Mrpl11	Down	0.0369	1.255	0.976	2.3	0.692	0.628	0.597
Ndufa11	Down	0.00658	1.346	1.081	1.393	0.862	0.812	0.79
Slirp	Down	0.0104	1.566	1.151	1.251	0.681	0.873	0.77
Isca1	Up	0.0119	0.768	0.676	0.678	1.041	1.171	1.569
Slc25a19	Down	0.000876	1.289	1.24	1.344	0.786	0.87	0.735
Xrn2	Down	0.00188	1.352	1.132	1.313	0.825	0.785	0.847

Cend1	Up	2.13E-07	0.777	0.774	0.788	1.191	1.175	1.179
Xpnpep3	Down	0.00896	1.105	1.26	1.555	0.841	0.757	0.751
Ampd3	Up	0.0104	0.857	0.776	0.717	1.13	1.084	1.395
Numbl	Up	0.0176	0.615	0.93	0.752	1.252	1.254	1.151
Tgfbr3	Up	0.0119	0.857	0.845	0.636	1.193	1.152	1.278
Ppt1	Up	0.0002	0.591	0.48	0.533	1.552	1.367	1.346
Thy1	Up	0.0106	0.887	0.786	0.696	1.231	1.287	1.06
Ly6a	Up	0.00254	0.603	0.724	0.807	1.316	1.239	1.251
Apoa2	Up	0.0163	0.881	0.786	0.695	1.063	1.145	1.388
Svs4	Down	0.00414	1.846	1.117	2.161	0.531	0.488	0.426
Ighv3-6	Down	0.0373	1.168	1.554	1.006	0.761	0.83	0.855
Hsd3b1	Up	0.00778	0.672	0.587	0.85	1.323	1.396	1.141
Epha3	Up	0.00844	0.668	0.907	0.686	1.305	1.217	1.167
Scarf2	Up	0.0258	0.492	0.901	0.686	1.421	1.262	1.156
Tsc22d1	Up	0.00648	0.698	0.856	0.74	1.172	1.387	1.112
Ube2d2	Down	0.0053	1.149	1.391	1.772	0.629	0.742	0.651
Hfe	Up	0.000756	0.652	0.691	0.568	1.186	1.391	1.393
C1qa	Up	0.00222	0.582	0.665	0.431	1.492	1.352	1.334
Folr2	Up	0.00732	0.747	0.645	0.766	1.469	1.05	1.285
Eml4	Down	0.0393	1.305	0.972	1.493	0.788	0.884	0.831
Cox7a2l	Down	0.0185	1.418	1.027	2.098	0.708	0.632	0.566
Nop58	Down	0.0113	1.546	1.059	1.461	0.702	0.714	0.837
Sepsecs	Down	0.0157	1.454	1.024	1.936	0.713	0.666	0.624
Usmg5	Down	0.0092	1.428	1.148	1.669	0.628	0.616	0.848
Nucks1	Down	0.0114	2.221	1.351	1.431	0.689	0.517	0.321
Lyve1	Up	0.0248	0.771	0.723	0.56	1.193	0.999	1.636
Armcx3	Up	0.0154	0.58	0.818	0.481	1.346	1.585	1.085
Pdxk	Up	0.00602	0.86	0.772	0.696	1.077	1.268	1.291
Mustn1	Up	0.0253	0.569	0.893	0.531	1.337	1.537	1.044
Atg3	Down	0.00822	1.31	1.116	1.505	0.823	0.842	0.704
Cuta	Up	0.0253	0.659	0.831	0.662	1.213	1.513	0.962
Marc1	Down	0.009	1.149	1.421	2.246	0.559	0.628	0.448
Hhatl	Down	0.00906	1.454	1.097	2.689	0.483	0.39	0.47
Fam213b	Down	0.0193	1.237	1.094	1.88	0.723	0.642	0.772
Rai14	Up	0.0241	0.774	0.914	0.662	1.186	1.044	1.364
Dkc1	Down	0.0334	1.501	0.977	1.522	0.686	0.719	0.904
Cpb2	Up	0.0054	0.386	0.636	0.326	1.203	1.622	1.58
P2rx4	Up	0.000662	0.581	0.502	0.656	1.399	1.257	1.478

Hspb8	Down	0.0378	1.038	1.156	1.895	0.725	0.707	0.789
Add2	Down	0.00624	1.375	1.14	1.259	0.835	0.734	0.901
Ggps1	Up	0.00566	0.587	0.794	0.738	1.167	1.422	1.217
Cpq	Up	0.00306	0.848	0.818	0.686	1.222	1.243	1.164
Gfpt2	Up	0.0129	0.669	0.719	0.535	1.382	0.987	1.577
Asna1	Up	0.000725	0.686	0.814	0.733	1.186	1.28	1.246
Tcap	Down	0.00452	1.857	0.979	3.412	0.25	0.144	0.191
Igf2bp1	Up	0.00148	0.741	0.592	0.541	1.305	1.284	1.427
Lamtor3	Up	0.0014	0.772	0.73	0.77	1.265	1.346	1.102
Mtnd4	Down	0.00258	1.445	1.226	1.239	0.805	0.851	0.712
Sbk2	Down	0.00262	1.477	1.246	2.128	0.546	0.514	0.564
Agt	Up	0.000479	0.839	0.757	0.781	1.168	1.173	1.265
C1qb	Up	0.0000203	0.59	0.606	0.583	1.434	1.278	1.385
Acp2	Up	0.0142	0.884	0.709	0.612	1.298	1.395	1.082
Plaur	Up	0.00162	0.624	0.665	0.655	1.55	1.313	1.128
Foxk1	Down	0.01	1.518	1.051	1.553	0.71	0.709	0.795
Cox6a2	Down	0.0157	1.513	0.94	3.064	0.476	0.34	0.344
Atp5j2	Down	0.0113	1.285	1.102	1.573	0.785	0.698	0.848
Atp5f1e	Down	0.043	1.178	1.015	1.734	0.786	0.737	0.845
Npy	Up	0.0398	0.776	0.851	0.666	1.316	0.92	1.411
Pcmtd1	Up	0.0108	0.789	0.789	0.561	1.135	1.248	1.391
Selenop	Up	0.00122	0.505	0.593	0.412	1.263	1.392	1.624
Irf3	Up	0.00912	0.767	0.846	0.75	1.271	1.338	1.024
Fbp2	Down	0.0315	0.926	1.523	1.744	0.796	0.657	0.648
Mcm6	Down	0.0226	1.505	1.038	1.281	0.79	0.747	0.906
Eepd1	Down	0.00526	1.695	1.14	1.346	0.722	0.747	0.698
Fam92b	Down	0.0402	1.047	1.398	1.4	0.808	0.789	0.787
Pik3c2a	Up	0.000659	0.683	0.802	0.734	1.292	1.266	1.177
Cd55	Up	0.0238	0.784	0.464	0.392	1.642	0.968	1.606
Cbx5	Down	0.00876	1.271	1.336	1.219	0.673	0.775	0.94
Ltbp3	Up	0.0291	0.599	0.932	0.705	1.443	1.209	1.069
Parpbp	Up	0.0018	0.409	0.408	0.328	1.214	1.276	2.058
Adamtsl4	Up	0.0013	0.611	0.721	0.613	1.496	1.3	1.177
F12	Up	0.000756	0.449	0.593	0.419	1.329	1.412	1.584
Htatsf1	Down	0.0079	1.295	1.101	1.477	0.801	0.736	0.861
Slc36a2	Down	0.016	1.332	1.051	1.737	0.718	0.792	0.713

Marf1	Down	0.000596	1.315	1.359	1.481	0.76	0.758	0.641
Tmem214	Down	0.000404	1.992	1.382	1.797	0.41	0.448	0.498
Prr33	Down	0.0308	1.578	1.034	1.618	0.794	0.839	0.53
C1rb	Up	0.00994	0.879	0.747	0.659	1.34	1.282	1.084
Mamdc2	Up	0.000884	0.53	0.629	0.633	1.489	1.439	1.179
Btd	Up	0.0169	0.944	0.673	0.721	1.179	1.35	1.134
Spp2	Up	0.00014	0.437	0.475	0.355	1.486	1.573	1.457
Syngr3	Up	0.0205	0.813	0.723	0.652	1.052	1.108	1.564
Nudt4	Up	0.0205	0.678	0.898	0.739	1.108	1.441	1.098
Myoz3	Down	0.0295	1.103	1.151	2.185	0.681	0.714	0.57
Camk1	Up	0.0272	0.645	0.961	0.725	1.159	1.356	1.111
Slc41a3	Down	0.00962	1.302	1.071	1.445	0.842	0.764	0.845
Nop2	Up	0.0071	0.852	0.58	0.581	1.204	1.337	1.37
Rbm10	Down	0.0213	1.82	1.159	1.84	0.574	0.564	0.533
Mrps18b	Down	0.0439	1.302	0.973	1.518	0.902	0.755	0.832
Atp5l	Down	0.023	1.184	1.087	1.821	0.723	0.722	0.785
Uqcr11	Down	0.0326	1.132	1.019	3.212	0.569	0.414	0.293
Ptms	Up	0.0213	0.667	0.833	0.615	1.184	1.607	1.034
Tmem126b	Down	0.0065	1.456	1.125	1.229	0.756	0.84	0.85
Zfyve19	Down	0.0318	1.54	1.126	2.136	0.573	0.545	0.564
Hmgn3	Up	0.0122	0.66	0.864	0.556	1.188	1.446	1.197
Cd302	Up	0.000401	0.648	0.693	0.655	1.319	1.437	1.175
Ubxn8	Down	0.00654	1.524	1.292	1.403	0.815	0.77	0.546
Ctsf	Up	0.0021	0.723	0.774	0.644	1.271	1.401	1.135
Adam24	Down	0.00166	1.348	1.496	1.942	0.622	0.57	0.46
Ntn3	Up	0.0488	0.626	0.864	0.753	1.333	1.498	0.908
Nfkb2	Down	0.00332	1.382	1.238	1.308	0.832	0.854	0.672
Camta1	Up	0.000562	0.764	0.632	0.695	1.336	1.309	1.217
Ttll8	Up	0.00706	0.668	0.539	0.58	1.065	1.246	1.738
Armc4	Up	0.00862	0.461	0.376	0.26	1.03	1.262	2.269
C3ar1	Up	0.0319	0.625	0.659	0.875	1.264	1.297	1.236
Pkig	Up	0.0029	0.682	0.657	0.763	1.368	1.407	1.088
mt-Co3	Down	0.0398	1.06	1.91	1.089	0.626	0.769	0.741
Mt1	Up	0.000716	0.253	0.339	0.247	1.156	1.866	1.802
Mtnd4l	Down	0.0422	1.846	0.99	2.222	0.488	0.525	0.492
Mycn	Down	0.00582	3.002	1.101	1.555	0.34	0.384	0.314

Mdk	Up	0.0054	0.47	0.739	0.451	1.245	1.346	1.555
Crp	Up	0.00106	0.735	0.735	0.739	1.228	1.292	1.227
Myd88	Up	0.0355	0.799	0.81	0.756	1.01	1.06	1.504
Msr1	Up	0.0105	0.83	0.558	0.585	1.408	1.089	1.449
C5ar1	Up	0.00228	0.712	0.687	0.542	1.304	1.191	1.451
Evc	Up	0.00924	0.596	0.867	0.633	1.144	1.27	1.382
Tbx4	Down	0.0025	1.733	1.182	2.8	0.315	0.296	0.352
Gnrhr	Up	0.00368	0.697	0.814	0.609	1.154	1.359	1.284
C1qc	Up	0.000464	0.681	0.671	0.548	1.342	1.297	1.353
Dennd1b	Down	0.00564	2.172	1.277	3.316	0.119	0.01	0.017
Hgsnat	Up	0.00208	0.852	0.757	0.713	1.304	1.147	1.209
Jcad	Down	0.000375	1.374	1.433	1.411	0.668	0.633	0.78
Pcnx2	Down	0.0121	2.013	1.269	2.399	0.336	0.334	0.314
Vps41	Down	0.00156	1.528	1.264	1.527	0.78	0.604	0.66
Syp	Up	0.00258	0.717	0.808	0.666	1.121	1.25	1.361
Ccdc66	Down	0.00268	1.642	1.21	2.649	0.347	0.38	0.398
Tlr13	Down	0.043	1.486	0.994	1.613	0.917	0.774	0.584
Kirrel1	Up	0.00706	0.592	0.864	0.659	1.23	1.356	1.216
Fbll1	Down	0.0155	1.562	1.039	1.415	0.757	0.695	0.845
Chtf18	Up	0.00524	0.459	0.355	0.327	1.521	1.571	1.535
Dhx32	Down	0.0379	1.545	1.197	1.139	1.008	0.747	0.657
Chpt1	Down	0.00566	1.204	1.273	1.45	0.727	0.694	0.902
Zswim4	Down	0.000303	2.372	1.559	1.685	0.312	0.3	0.378
Phtf2	Up	0.00292	0.35	0.322	0.202	1.133	1.419	2.204
Smoc2	Up	0.0273	0.409	0.775	0.765	1.252	1.227	1.444
Ttc27	Down	0.00466	2.406	0.998	2.488	0.357	0.23	0.278
Cyp2d26	Up	0.000823	0.724	0.681	0.682	1.217	1.172	1.441
Tceal3	Up	0.031	0.442	0.79	0.642	1.443	1.642	0.954
Sumf1	Up	0.00658	0.814	0.668	0.882	1.222	1.245	1.173
Tspan8	Down	0.00406	1.102	1.375	1.366	0.799	0.746	0.837
Fitm1	Down	0.0363	1.428	1.052	3.402	0.28	0.263	0.306
Plpp7	Down	0.00214	1.415	1.166	1.444	0.826	0.724	0.734
Ddx27	Down	0.0317	1.527	1.023	1.224	0.826	0.764	0.899
Chchd5	Up	0.00424	0.831	0.6	0.672	1.271	1.192	1.375
Dbndd2	Up	0.0116	0.509	0.759	0.629	1.369	1.599	1.046
Sycp2	Up	0.0181	0.672	0.908	0.701	1.049	1.222	1.37
Rrp7a	Up	0.0202	0.497	0.852	0.523	1.622	1.335	1.071
Naa38	Down	0.00198	1.761	1.126	3.442	0.197	0.139	0.162
Etnk1	Up	0.0444	0.745	0.796	0.342	1.156	1.452	1.371

Slco2a1	Up	0.0342	0.687	0.781	0.792	1.172	0.95	1.541
Sfrp4	Up	0.00202	0.677	0.765	0.746	1.382	1.105	1.273

Proteomic analysis of differentially expressed proteins.

Table S5

Human		
**Gene**	**Forward primer**	**Reverse primer**
IFNα	GCCTCGCCCTTTGCTTTACT	CTGTGGGTCTCAGGGAGATCA
IFNβ	ATGACCAACAAGTGTCTCCTCC	GGAATCCAAGCAAGTTGTAGCTC
IRF7	GCTGGACGTGACCATCATGTA	GGGCCGTATAGGAACGTGC
MX1	AGCGGGATCGTGACCAGAT	TGACCTTGCCTCTCCACTTATC
cGAS	ACATGGCGGCTATCCTTCTCT	GGGTTCTGGGTACATACGTGAAA
STING	CCAGAGCACACTCTCCGGTA	CGCATTTGGGAGGGAGTAGTA
IRF3	AGAGGCTCGTGATGGTCAAG	AGGTCCACAGTATTCTCCAGG
IFNAR1	AACAGGAGCGATGAGTCTGTC	TGCGAAATGGTGTAAATGAGTCA
STAT1	CGGCTGAATTTCGGCACCT	CAGTAACGATGAGAGGACCCT
α-SMA	GTGTTGCCCCTGAAGAGCAT	GCTGGGACATTGAAAGTCTCA
CNN1	GTCAACCCAAAATTGGCACCA	ACCTTGTTTCCTTTCGTCTTCG
TAGLN	AGTGCAGTCCAAAATCGAGAAG	CTTGCTCAGAATCACGCCAT
MYH11	CATCTACTCGGAGAAGATCGTCG	CGCCTGTGCATAGAATGGACT
p53	GAGGTTGGCTCTGACTGTACC	TCCGTCCCAGTAGATTACCAC
p16	GATCCAGGTGGGTAGAAGGTC	CCCCTGCAAACTTCGTCCT
β-actin	CATGTACGTTGCTATCCAGGC	CTCCTTAATGTCACGCACGAT
**Mouse**		
**Gene**	**Forward primer**	**Reverse primer**
IFNα	ATGGCTAGGCTCTGTGCTTTCCT	AGGGCTCTCCAGAGTTCTGCTCTG
IFNβ	TGGGTGGAATGAGACTATTGTTG	CTCCCACGTCAATCTTTCCTC
IRF7	GAGACTGGCTATTGGGGGAG	GACCGAAATGCTTCCAGGG
MX1	GTTTCCGAAGTGGACATCGCA	CTGCACAGGTTGTTCTCAGC
α-SMA	GTCCCAGACATCAGGGAGTAA	TCGGATACTTCAGCGTCAGGA
CNN1	TCTGCACATTTTAACCGAGGTC	GCCAGCTTGTTCTTTACTTCAGC
TAGLN	CAACAAGGGTCCATCCTACGG	ATCTGGGCGGCCTACATCA
MYH11	AAGCTGCGGCTAGAGGTCA	CCCTCCCTTTGATGGCTGAG
β-actin	CGTTGACATCCGTAAAGACC	AACAGTCCGCCTAGAAGCAC

Primer sequences for mRNA expression analysis.

Table S6

mtDNA		
**Gene**	**Forward primer**	**Reverse primer**
ND1	CCCTAAAACCCGCCACATCT	GAGCGATGGTGAGAGCTAAGGT
ND2	ACCATCTTTGCAGGCACACT	GCTTCTGTGGAACGAGGGTT
ND4	TTCCTCCGACCCCCTAACAA	GATAAGTGGCGTTGGCTTGC
ND5	TTCATCCCTGTAGCATTGTTCG	GTTGGAATAGGTTGTTAGCGGTA
**nDNA**		
**Gene**	**Forward primer**	**Reverse primer**
B2M	CCAGCAGAGAATGGAAAGTCAA	TCTCTCTCCATTCTTCAGTAAGTCAACT
β-actin	GCACCCAGCACAATGAAGAT	CAATGATCTGAGGAGGGAAGGG
POLG	TCCTGGAACAGTTGTGCTTTC	CCATCTACTCAGGACGGAGTTC
GAPDH	CTGGGCTACACTGAGCACC	AAGTGGTCGTTGAGGGCAATG

Primer sequences for mtDNA and nDNA analysis.

Table S7

Human		
**Gene**	**Sense**	**Antisense**
IFNAR1	CCUACUUCCUCCAGUCUUUTT	AAAGACUGGAGGAAGUAGGTT
STAT1	GCGUAAUCUUCAGGAUAAUTT	AUUAUCCUGAAGAUUACGCTT
cGAS	CCAACACUCGUGCAUAUUATT	UAAUAUGCACGAGUGUUGGTT
STING	GCAUCAAGGAUCGGGUUUATT	UAAACCCGAUCCUUGAUGCTT
Negative control	UCCUCCGAACGUGUCACGUTT	ACGUGACACGUUCGGAGAATT

siRNA sequences used for RNA interference.
